# Design, synthesis, and biological evaluation of novel ciprofloxacin derivatives as potential anticancer agents targeting topoisomerase II enzyme

**DOI:** 10.1080/14756366.2022.2136172

**Published:** 2022-10-28

**Authors:** Hadeer K. Swedan, Asmaa E. Kassab, Ehab M. Gedawy, Salwa E. Elmeligie

**Affiliations:** a Central Administration of Research and Health Development, Ministry of Health, and Population (MoHP), Cairo, Egypt; b Faculty of Pharmacy, Department of Pharmaceutical Organic Chemistry, Cairo University, Cairo, Egypt; c Faculty of Pharmacy and Pharmaceutical Industries, Department of Pharmaceutical Chemistry, Badr University in Cairo (BUC), Badr City, Egypt

**Keywords:** Design, synthesis, ciprofloxacin, anti-proliferative activity, topoisomerase II

## Abstract

A series of novel ciprofloxacin (CP) derivatives substituted at the N-4 position with biologically active moieties were designed and synthesised. 14 compounds were 1.02- to 8.66-fold more potent than doxorubicin against T-24 cancer cells. Ten compounds were 1.2- to 7.1-fold more potent than doxorubicin against PC-3 cancer cells. The most potent compounds **6**, **7a**, **7b**, **8a**, **9a**, and **10c** showed significant Topo II inhibitory activity (83–90% at 100 μM concentration). Compounds **6**, **8a**, and **10c** were 1.01- to 2.32-fold more potent than doxorubicin. Compounds **6** and **8a** induced apoptosis in T-24 (16.8- and 20.1-fold, respectively compared to control). This evidence was supported by an increase in the level of apoptotic caspase-3 (5.23- and 7.6-fold, sequentially). Both compounds arrested the cell cycle in the S phase in T-24 cancer cells while in PC-3 cancer cells the two compounds arrested the cell cycle in the G1 phase. Molecular docking simulations of compounds **6** and **8a** into the Topo II active site rationalised their remarkable Topo II inhibitory activity.

## Graphical Abstract

**Figure:**
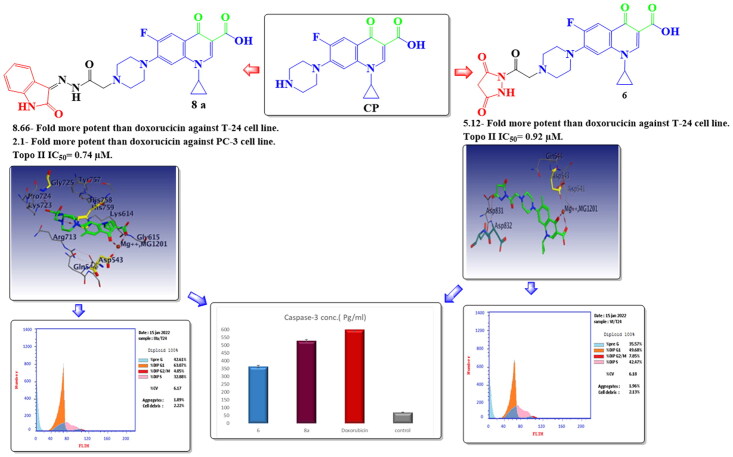

## Introduction

Cancer is a very dangerous and life-threatening disease, it is considered one of the most prevalent diseases in the world^1^. The defining characteristic of cancer is metastasis, the leading cause of death from cancer^1–3^. Many antitumor agents are commercially available, but the emergence of acquired drug resistance with severe side effects of these clinically used anticancer drugs poses serious barriers to effective chemotherapy^4^. Therefore, it is recommended to rationally develop new anticancer drugs with fewer side effects.

DNA topological problems arise from the intertwined nature of the DNA double helix structure, which causes tangles and supercoiling of the DNA duplex during the DNA replication and transcription. DNA supercoiling results in torsion that impair the function of DNA or RNA polymerases. Type II topoisomerase enzyme (Topo II) prevents and corrects these types of topological problems via transient double-stranded breaks, causing DNA metabolism to proceed, allowing the cell to efficiently replicate so enabling cellular division and vitality^5–7^. The role of divalent Mg^2+^ ions in Topo II-mediated reactions was recognised as an implication in enzyme-mediated DNA cleavage reactions. (2) participation in ATPase reactions and functions by providing the enzyme with magnesium–ATP substrate^8^. Topo II enzyme inhibition leads to apoptosis and cell death^9^^,^^10^, therefore, it is considered a valid strategy in cancer therapy. The presence of topoisomerase enzyme in both mammalian and bacterial cells makes it a pronounced target for antibacterial and anticancer drugs^10^^,^^11^. Recently, mammalian Topo II is considered a critical target for anticancer drug development^12–14^.

The use of fluoroquinolone derivatives as anti-proliferative agents is of great interest to researchers, as they are less toxic, lower tumour resistance is exerted towards them, and they have less chance of developing secondary tumours. Moreover, they exhibited excellent pharmacological and pharmacokinetic profiles^15–17^. Fluoroquinolones act by inhibiting the Topo II enzyme in both prokaryotic and eukaryotic cells, due to the similarities between the prokaryotic and eukaryotic topoisomerases^18^. A similar mechanism of action characterises several clinically important antitumor agents such as etoposide, doxorubicin, amsacrine, or mitoxantrone^19^^,^^20^. Recently, a great deal of work has been devoted to the antiproliferative activity of fluoroquinolones and several studies proved them as potent cytotoxic agents^19^^,^^21–23^. Ciprofloxacin (CP), a broad-spectrum fluoroquinolone antibiotic, showed anti-proliferative activity against strains of human cancer cells. CP has been reported to pile up in urine and prostate tissues, therefore it is a privileged candidate for the treatment of bladder, and prostate cancers^24^. CP, among fluoroquinolones, is distinguished by strong inhibition of Topo II^25^. Additionally, it can induce the intrinsic apoptotic pathway by creating a double-stranded break in DNA or cell cycle arrest in the S/G2 phase^26^^,^^27^. Thus, CP serves as a unique scaffold for the development of novel anticancer agents.

The SAR studies uncovered that fluorine atom, the 1-alkyl, and 1, 4-dihydro-4-oxo-quinoline-3-carboxylic acid skeleton are the fundamental pharmacophore lineaments for CP anticancer activity^19^^,^^28–31^.

Several studies have been conducted to determine the cytotoxic structural features of CP on eukaryotic cells. These studies changed the activity of fluoroquinolones from antibacterial to antitumor activity^32^. Topo II inhibitory activity and pharmacokinetic features of CP are greatly affected by the modification at the piperazinyl N-4 position of the condensed CP^15^. Hence, new fluoroquinolone analogues can be developed by structural modifications at the C-7 position of CP which diminished the zwitterion effect and greatly influenced the hydrophilicity nature leading to the improvement of antiproliferative activity of CP^33–38^.

**Figure 1.**
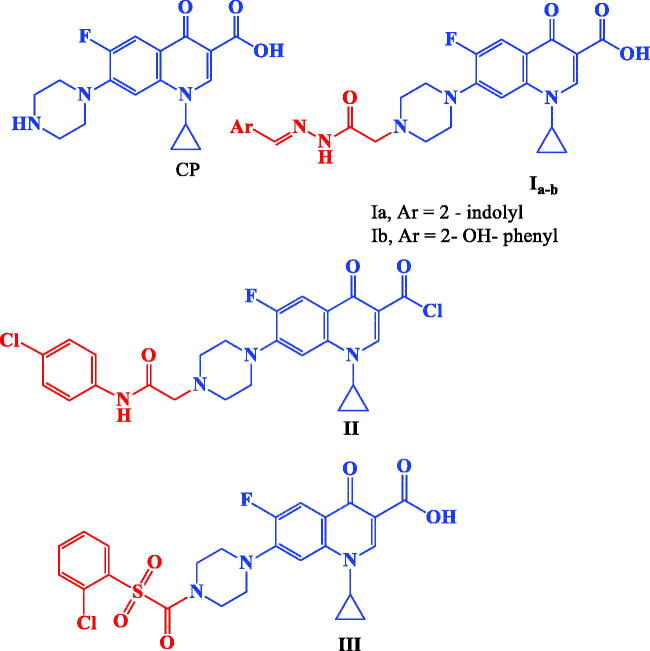
Structures of CP and potent anticancer CP derivatives **I–III**.

CP hybrids **Ia**,**b**^39^, **II**^21^, and **III**^21^ (Figure 1) incorporating N-acylarylhydrazone, arylacetamide, and aryl sulphonyl moieties, respectively at the N-4 position of piperazine revealed significant *in vitro* anti-proliferative activity.

**Figure 2.**
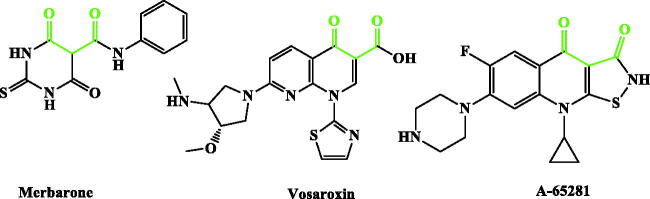
Potent Topo II inhibitors.

**Figure 3.**
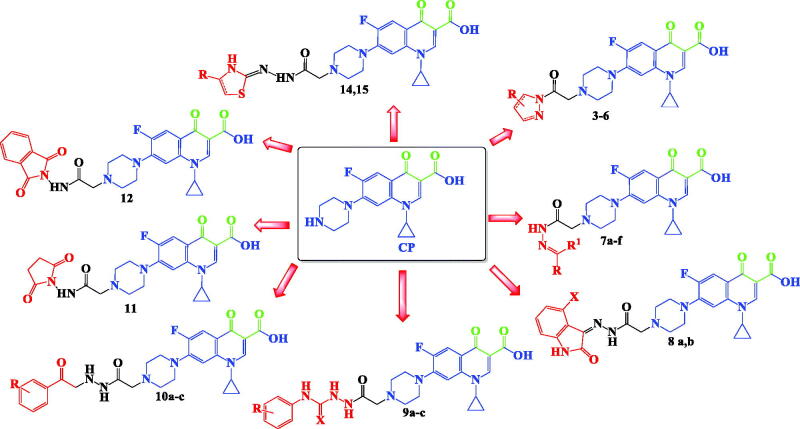
Design strategy for the synthesised CP hybrids.

Inspired by all these findings, we have designed a series of novel CP derivatives with essential pharmacophoric features for Topo II inhibition. Our design strategy kept the two coplanar carbonyl groups at positions 3 and 4 of the CP scaffold which is a common structural motif of potent Topo II inhibitors such as merbarone^40^, vosaroxin^41^, and A-65281^42^ (Figure 2). Moreover, this pharmacophore may guarantee the coordination with the Mg^2+^ ion that plays an essential role in promoting the DNA cleavage–rejoining activity of Topo II enzyme^8^. The newly synthesised CP derivatives are featuring various biologically active moieties at the N-4 position of piperazine such as monocyclic heteroaryl scaffolds (pyrazole, pyrazolidine, pyrrolidine, or thiazolidine ring), benzo fused heteroaryl rings (indoline or isoindoline), N-acyl(alkyl or aryl) hydrazone, semicarbazide, thiosemicarbazide or arylacetamide. These moieties are well acknowledged for anti-proliferative activity via different mechanisms such as apoptosis induction, caspase activation, and DNA inter-chelation^21^^,^^43–61^. All these moieties are linked to the piperazine ring of CP via 2, 3, or 4 atoms spacers (Figure 3). We have aimed to explore the impact of such variation at the N-4 position of piperazine of CP core with diverse lipophilic and electronic environments on the anticancer activity and to identify potent anti-proliferative agents. The synthesised CP hybrids were screened for their anti-proliferative activity *in vitro* against bladder T-24 and prostate PC-3 cancer cell lines. All derivatives were subjected to the determination of their half-maximal inhibitory concentration (IC_50_) values. The conversion of supercoiled plasmid DNA to relaxed DNA by recombinant Topo II was examined in the presence of each of the most potent compounds **6**, **7a**, **7b**, **8a**, **9a**, and **10c**. The most prominent Topo II inhibitors **6** and **8a** were further investigated regarding their effects on cell cycle progression, induction of apoptosis, and level of active caspase-3 in the T-24 cell line.

## Materials and methods

### Chemistry

#### General

Melting points were determined on a Griffin apparatus and were uncorrected. Shimadzu IR 435 spectrophotometer (Shimadzu Corp., Kyoto, Japan), Faculty of Pharmacy, Cairo University, Cairo, Egypt, was used to record IR spectra, values were represented in cm^−1^. ^1^H NMR (400 MHz) and ^13^C NMR (100 MHz) spectra were recorded in ppm on the *δ* scale and coupling constants (*J*) were given in Hz on Bruker 400 MHz (Bruker Corp., Billerica, MA, USA) spectrophotometer, Faculty of Pharmacy, Cairo University, Cairo, Egypt. Tetra-methylsilane (TMS) was used as an internal standard. Progress of the reactions was monitored by TLC using pre-coated aluminium sheet silica gel MERCK 60F 254 and was visualised by a UV lamp.

##### Procedure for the preparation of 1-cyclopropyl-7-(4-(2-ethoxy-2-oxoethyl)piperazin-1-yl)-6-fluoro-4-oxo-1,4-dihydroquinoline-3-carboxylic acid (1)

A mixture of ciprofloxacin (1.56 g, 0.005 mol), ethyl chloroacetate (0.61 g, 0.005 mol) and trimethylamine (10 g, 0.1 mol) in dimethylformamide (50 ml) was heated under reflux for 6 h. The reaction mixture was cooled, and the separated solid was filtered, dried, and crystallised from ethanol to give compound **1**. MP 190–192 °C; yield 86%^39^.

##### Procedure for the preparation of 1-cyclopropyl-6-fluoro-7-(4-(2-hydrazinyl-2-oxoethyl) piperazin-1-yl)-4-oxo-1,4-dihydroquinoline -3-carboxylic acid (2)

A mixture of ester derivative **1** (2.08 g, 0.005 mol) and hydrazine hydrate 99% (1.26 g, 0.025 mol) in absolute ethanol (5 ml) was heated under reflux for 6 h. The reaction mixture was cooled, and the separated solid was filtered, dried, and crystallised from ethanol to give compound **2**. MP 226–228 °C (as reported); yield: 75%^39^.

##### Procedure for the preparation of 1-cyclopropyl-6-fluoro-7-(4-(2-(3-methyl-5-oxo-4,5-dihydro-1H-pyrazol-1-yl)-2-oxoethyl)piperazin-1-yl)-4-oxo-1,4-dihydroquinoline-3-carboxylic acid (3)

A mixture of the hydrazinyl derivative **2** (0.50 g, 0.001 mol) and ethyl acetoacetate (0.13 g, 0.001 mol) in absolute ethanol (5 ml) was heated under reflux for 6 h. The reaction mixture was cooled, and the separated solid was filtered, dried, and crystallised from ethanol to give compound **3**. MP 188–190 °C yield; 60%; IR (KBr) *v*_max_: 3549 (OH), 3093 (C–H arom.), 2978 (C–H aliph.), 1730, 1720 (C = O) cm^−1^; ^1^H NMR (DMSO-d_6_): *δ* 1.18–1.20 (m, 2H, CH_2_ cyclopropyl), 1.30–1.35 (m, 2H, CH_2_ cyclopropyl), 2.47 (s, 3H, CH_3_C = N), 2.70–2.75 (m, 4H, 2CH_2_ piperazine), 3.30–3.40 (m, 4H, 2CH_2_ piperazine), 3.40–3.50 (m, 1H, CH cyclopropyl), 3.80 (s, 2H, CH_2_ pyrazolone), 4.30 (s, 2H, N-CH_2_CO), 7.54 (d, 1H, *J* = 8 Hz, ArH), 7.85 (d, 1H, *J* = 13.6 Hz, ArH), 8.60 (s, 1H, C2-H), 15.17 (s, 1H, COOH, D_2_O exchangeable) ppm; ^13^C NMR (DMSO-d_6_): *δ* 8.0, 14.6, 36.3, 49.8, 52.0, 58.6, 60.3, 107.1, 111.4, 119.0, 139.6, 145.5, 145.6, 148.4, 152.2, 154.7, 166.3, 170.3, 176.7–176.8 ppm. Anal. Calcd. for C_23_H_24_FN_5_O_5_ (469.47): C, 58.84; H, 5.15; N, 14.92. Found: C, 58.93; H, 5.19; N, 15.22.

##### Procedure for the preparation of 1-cyclopropyl-6-fluoro-7-(4-(2-(5-imino-3-oxopyrazolidin-1-yl)-2-oxoethyl)piperazin-1-yl)-4-oxo-1,4-dihydroquinoline-3-carboxylic acid (4)

A mixture of hydrazinyl derivative **2** (0.50 g, 0.001 mol) and ethyl cyanoacetate (0.11 g, 0.001 mol) in glacial acetic acid (10 ml) was heated under reflux for 5 h. The reaction mixture was filtered while hot, the filtrate was left to cool, and the separated solid was filtered, washed with water (15 ml) dried and recrystallised from ethanol to give compound **4**. MP 181–183 °C; yield 37%; IR (KBr) *v*_max:_ 3541 (OH), 3437, 3417 (2NH), 3059 (C–H arom.), 2870 (C–H aliph.), 1728, 1720 (C = O) cm^−1^; ^1^H NMR (DMSO-d_6_): *δ* 1.25–1.40 (m, 2H, CH_2_ cyclopropyl), 1.60–1.65 (m, 2H, CH_2_ cyclopropyl), 2.80–2.95 (m, 4H, 2CH_2_ piperazine), 3.36–3.41 (m, 4H, 2CH_2_ piperazine), 3.44 (s, 2H, NCH_2_O), 3.80–3.90 (m, 1H, CH cyclopropyl), 3.86 (s, 2H, CH_2_ pyrazalone) 7.57 (d, 1H, ArH), 7.90 (d, 1H, *J* = 8 Hz, ArH), 8.66 (s, 1H, C2-H), 9.68 (s, 1H, NH, D_2_O exchangeable), 9.75 (s, 1H, NH, D_2_O exchangeable), 15.19 (s, 1H, COOH D_2_O exchangeable) ppm; ^13^C NMR (DMSO-d_6_): *δ* 5.9, 12.5, 24.0, 34.2, 43.7, 47.5, 104.6, 109.3, 115.3, 116.8, 122.5, 137.5, 143.4, 146.2, 163.6, 164.2, 168.2, 169.5, 174.6 ppm. Anal. Calcd. for C_22_H_23_FN_6_O_5_ (470.45): C, 56.17; H, 4.93; N, 17.86. Found: C, 56.40; H, 4.81; N, 18.05.

##### Procedure for the preparation of 1-cyclopropyl-7-(4-(2-(3,5-dimethyl-1H-pyrazol-1-yl)-2-oxoethyl)piperazin-1-yl)-6-fluoro-4-oxo-1,4-dihydro-quinoline-3-carboxylic acid (5)

A mixture hydrazinyl derivative **2** (0.50 g, 0.001 mol) and acetylacetone (0.1 g, 0.001 mol) in absolute ethanol (5 ml) was heated under reflux for 6 h. The reaction mixture was cooled, and the separated solid was filtered, dried, and crystallised from ethanol to give compound **5**. MP: 185–187 °C; yield: 60%; IR (KBr) *v*_max:_ 3444 (OH), 3055 (C–H arom.), 2912 (C–H aliph.), 1730, 1728, 1720 (C = O) cm^−1^; ^1^H NMR (DMSO-d_6_): *δ* 1.15–1.20 (m, 2H, CH_2_ cyclopropyl), 1.30–1.35 (m, 2H, CH_2_ cyclopropyl), 2.75–2.80 (m, 7H, 2CH_2_, CH_3_), 2.90 (s, 3H, CH_3_), 3.30–3.35 (m, 4H, 2CH_2_ piperazine), 3.40–3.50 (m, 1H, CH cyclopropyl), 3.80 (s, 2H, CH_2_CO), 7.55 (d, 1H, ArH), 7.88 (d, 1H, ArH), 7.90 (s, 1H, CH pyrazole), 8.63 (s, 1H, C2-H), 15.16 (s, 1H, COOH, D_2_O exchangeable) ppm; ^13^C NMR (DMSO-d_6_): *δ* 8.0, 14.6, 19.0, 36.3, 49.8, 52.0, 58.6, 106.8, 107.1, 111.2, 111.4, 118.9, 139.6, 145.6, 148.3, 152.2, 154.6, 166.3, 170.3, 176.7 ppm. Calcd. for C_24_H_26_FN_5_O_4_ (467.49): C, 61.66; H, 5.61; N, 14.98. Found: C, 61.92; H, 5.72; N, 15.17.

##### Procedure for the preparation of 1-cyclopropyl-7-(4-(2-(3,5-dioxopyrazolidin-1-yl)-2-oxoethyl)piperazin-1-yl)-6-fluoro-4-oxo-1,4-dihydroquinoline-3-carboxylic acid (6)

A mixture of hydrazinyl derivative **2** (0.20 g, 0.0005 mol) in sodium ethoxide (0.034 g atomic weight of sodium in 17 ml absolute ethanol), diethyl malonate (0.1 g, 0.0008 mol) was heated under reflux for 7 h. The reaction mixture was cooled, and the separated solid was filtered, washed with water (5 ml) dried, and crystallised from ethanol to give compound **6**. MP: 140–142 °C; yield: 35%; IR IR (KBr) *v*_max_: 3583 (OH), 3441 (NH), 3008 (C–H arom.), 2958 (C–H aliph.), 1680, 1660 (C = O) cm^−1^; ^1^H NMR (DMSO-d_6_): *δ* 1.15–1.30 (m, 4H, 2CH_2_ cyclopropyl), 2.70–2.80 (m, 4H, 2CH_2_ piperazine), 2.90 (s, 2H, CH_2_ pyrazolone), 3.20–3.25 (m, 4H, 2CH_2_ piperazine), 3.35 (s, 2H, CH_2_CO), 3.65 (s, 1H, NH, D_2_O exchangeable), 3.95–4.00 (m, 1H, CH cyclopropyl), 7.54 (d, 1H, ArH), 7.85 (d, 1H, ArH), 8.59 (s, 1H, C2-H) ppm; ^13^C NMR (DMSO-d_6_): *δ* 8.0, 14.6, 15.3, 36.3, 49.8, 60.3, 106.8, 117.6, 119.0, 130.1, 133.2, 148.3, 152.2, 154.7, 160.0, 166.3, 168.5, 170.3, 176.8 ppm. Anal. Calcd. for C_22_H_22_FN_5_O_6_ (471.44): C, 56.90; H, 4.70; N, 14.86. Found: C, 56.11; H, 4.68; N, 14.84.

#### General procedure for the preparation of 7-(4-(-1-(substituted)ethylidene)hydrazine carbonyl)methyl)piperazin-1-yl)-1-cyclopropyl-6-fluoro-4-oxo-1,4-dihydroquinoline-3-carboxylic acid (7a–f)

A mixture of hydrazinyl derivative **2** (0.50 g, 0.001 mol), the appropriate ketone (0.001 mol), and glacial acetic acid (1 ml) in absolute ethanol (5 ml) was heated under reflux for 6 h. The reaction mixture was cooled, and the separated solid was filtered, dried, and recrystallised from ethanol to give compounds **7a–f**.

##### 7-(4-(2-(2-Cyclohexylidenehydrazinyl)-2-oxoethyl)piperazin-1-yl)-1-cyclopropyl-6-fluoro-4-oxo-1,4-dihydroquinoline-3-carboxylic acid (7a)

M.P.: 256–258 °C; yield: 65%; IR (KBr) *v*_max:_ 3497 (OH), 3417 (NH), 3043 (C–H arom.), 2981, 2962 (C–H aliph.) cm^−1^; ^1^H NMR (DMSO-d_6_): *δ* 1.18–1.25 (m, 2H, CH_2_ cyclopropyl), 1.31–1.33 (m, 2H, CH_2_ cyclopropyl), 2.91–2.93 (m, 2H, CH_2_ cyclohexylidene) 3.24–3.25 (m, 4H, 2CH_2_ cyclohexylidene), 3.40–3.50 (m, 1H, CH cyclopropyl), 3.60–3.70 (m, 4H, 2CH_2_ cyclohexylidene), 3.82 (s, 2H, CH_2_CO) 7.55 (d, 1H, *J* = 8 Hz, ArH), 7.87 (d, 1H, *J* = 12 Hz, ArH), 8.13 (s, 1H, NH, D_2_O exchangeable), 8.65 (s, 1H, C2-H) ppm; ^13^C NMR (DMSO-d_6_): *δ* 8.0, 19.0, 34.2, 36.2, 45.8, 51.0, 51.1, 57.0, 77.0, 106.4, 107.2, 111.3, 118.7, 139.6, 146.1, 148.2, 166.4, 152.1, 154.6, 176.7 ppm. Anal. Calcd. for C_25_H_30_FN_5_O_4_ (483.54): C, 62.10; H, 6.25; N, 14.48. Found: C, 62.12; H, 6.28; N, 14.46.

##### 7-(4-(2-(2-(1-(4-Chlorophenyl)ethylidene)hydrazinyl)-2-oxoethyl)-piperazin-1-yl)-1-cyclopropyl-6-fluoro-4-oxo-1,4-dihydroquinoline-3-carboxylic acid (7b)

M.P.: 178–180 °C; yield 75%; IR (KBr) *v*_max:_ 3444 (OH), 3298 (NH), 3078 (C–H arom.), 2924, 2827 (C–H aliph.), 1705 (C = O) cm^−1^; ^1^H NMR (DMSO-d_6_): *δ* 1.19–1.23 (m, 2H, CH_2_ cyclopropyl), 1.33–1.40 (m, 2H, CH_2_ cyclopropyl), 2.18 (s, 3H, CH_3_), 2.25–2.30 (m, 4H, 2CH_2_ piperazine), 2.70–2.90 (m, 4H, 2CH_2_ piperazine), 3.05 (s, 2H, CH_2_CO), 3.78–3.80 (m, 1H, CH cyclopropyl), 7.50–7.52 (m, 3H, ArH), 7.82–8.00 (m, 3H, ArH), 8.6 (s, 1H, C2-H), 10.42, 10.66 (2 s, 1H, NH, D_2_O exchangeable), 15.22 (s, 1H, COOH, D_2_O exchangeable) ppm. Anal. Calcd. for C_27_H_27_ClFN_5_O_4_ (539.99): C, 60.06; H, 5.04; N, 12.97. Found: C, 60.13; H, 5.18; N, 12.92.

##### 7-(4-(2-(2-(1-(4-Aminophenyl)ethylidene)hydrazinyl)-2-oxoethyl)-piperazin-1-yl)-1-cyclopropyl -6-fluoro-4-oxo-1,4-dihydroquinoline-3-carboxylic acid (7c)

M.P.: 173–175; yield 70%; IR (KBr) *v*_max:_ 3421 (OH), 3356, 3232 (NH, NH_2_), 3051 (C–H arom.), 2974 (C–H aliph.), 1730, 1728 (C = O) cm^−1^; ^1^HNMR (DMSO-d_6_): *δ* 1.19–1.22 (m, 2H, CH_2_ cyclopropyl), 1.26–1.33 (m, 2H, CH_2_ cyclopropyl), 1.84–1.87 (m, 1H, CH cyclopropyl), 2.80–2.93 (m, 4H, 2CH_2_ piperazine), 3.46–3.92 (m, 7H, 2CH_2_ piperazine, CH_3_), 3.87 (s, 2H, CH_2_CO), 4.20 (s, 2H, NH_2_, D_2_O exchangeable), 7.11 (s, 1H, NH, D_2_O exchangeable) 7.54 (d, 3H, ArH), 7.94 (d, 3H, ArH), 8.68 (s, 1H, C2-H), 11.23 (s, 1H, OH exchangeable by D_2_O) ppm. Anal. Calcd. for C_27_H_29_FN_6_O_4_ (520.56): C, 62.30; H, 5.62; N, 16.14. Found: C, 62.28; H, 5.57; N, 16.16.

##### 1-Cyclopropyl-6-fluoro-7-(4-(2-(2-(1-(2-hydroxyphenyl)ethylidene)hydrazinyl)-2-oxoethyl)piperazin-1-yl)-4-oxo-1,4-dihydroquinoline-3-carboxylic acid (7d)

M.P.: 166–168; yield 80%; IR (KBr) *v*_max;_ 3545 (OH), 3417 (NH), 3059 (C–H arom.), 2912 (C–H aliph.), 1728, 1705 (C = O) cm^−1^; ^1^H NMR (DMSO-d_6_): *δ* 1.18–1.22 (m, 2H, CH_2_ cyclopropyl), 1.32–1.33 (m, 2H, CH_2_ cyclopropyl), 2.53 (s, 3H, CH_3_), 2.75–2.80 (m, 4H, CH_2_ piperazine), 3.33–3.45 (m, 6H, 2CH_2_ piperazine, CH_2_CO), 3.81–3.90 (m, 1H, CH cyclopropyl), 6.95 (d, 2H, ArH), 7.40 (t, 1H, ArH), 7.55 (d, 1H, ArH), 7.76 (d, 1H, ArH), 7.89 (d, 1H, ArH), 8.64 (s, 1H, C2-H), 12.93 (s, 1H, NH, D_2_O exchangeable), 13.12 (s, 1H, OH, D_2_O exchangeable) 15.20 (s, 1H, COOH, D_2_O exchangeable) ppm. ^13^C NMR (DMSO-d_6_): *δ* 8.0, 15.3, 36.2, 49.8, 52.0, 60.3, 106.8, 107.1, 111.4, 117.6, 118.9, 119.5, 130.1, 133.2, 139.6, 145.5, 148.3, 152.1, 154.6, 160.0, 166.3, 168.6, 170.3, 176.7 ppm. Anal. Calcd. for C_27_H_28_FN_5_O_5_ (521.56): C, 62.18; H, 5.41; N, 13.43. Found: C, 61.96; H, 5.70; N, 13.62.

##### 1-Cyclopropyl-6-fluoro-4-oxo-7-(4-(2-oxo-2-(2-(1-(thiophen-2-yl)ethylidene)hydrazinyl)ethyl)piperazin-1-yl)-1,4-dihydroquinoline-3-carboxylic acid (7e)

M.P.: 245–247; yield 85%; IR (KBr) *v*_max:_ 3441 (OH), 3271 (NH), 3082 (C–H arom.), 2947 (C–H aliph.), 1730, 1728 (C = O) cm^−1^; ^1^H NMR (DMSO-d_6_): *δ* 1.15–1.20 (m, 2H, CH_2_ cyclopropyl), 1.25–1.30 (m, 2H, CH_2_ cyclopropyl), 2.40 (s, 3H, CH_3_), 2.60–2.65 (m, 4H, 2CH_2_ piperazine), 2.70–2.80 (m, 4H, 2CH_2_ piperazine), 3.27 (s, 2H, CH_2_ CO), 3.85–3.90 (m, 1H, CH cyclopropyl), 7.05–7.15 (m, 2H, ArH), 7.50–7.70 (m, 2H, ArH), 7.95 (d, 1H, ArH), 8.65 (s, 1H, C2-H), 8.99, 9.67 (2 s, 1H, NH, D_2_O exchangeable), 15.19 (s, 1H, COOH, D_2_O exchangeable) ppm. ^13^C NMR (DMSO-d_6_): *δ* 8.0, 14.6, 36.3, 49.7, 49.9, 52.6, 106.8, 107.7, 111.2, 118.9, 119.0, 127.9, 128.5, 139.6, 143.5, 144.0, 145.6, 148.3, 154.6, 168.5, 171.5, 176.7 ppm. Anal. Calcd. for C_25_H_26_FN_5_O_4_S (511.57): C, 58.70; H, 5.12; N, 13.69. Found: C, 58.63; H, 5.15; N, 13.72.

##### 7-(4-(2-(2-(1-(5-Chlorothiophen-2-yl)ethylidene)hydrazinyl)-2-oxoethyl)piperazin-1-yl)-1-cyclopropyl-6-fluoro-4-oxo-1,4-dihydro-quinoline-3-carboxylic acid (7f)

M.P.: 286–288; yield 85%; IR (KBr) *v*_max:_ 3456 (OH), 3275 (NH), 3059 (C–H arom.), 2924 (C–H aliph.), 1730, 1728 (C = O) cm^−1^; ^1^H NMR (DMSO-d_6_): *δ* 1.10–1.20 (m, 2H, CH_2_ cyclopropyl), 1.27–1.33 (m, 2H, CH_2_ cyclopropyl), 2.38 (s, 3H, CH_3_), 2.70–2.74 (m, 4H, 2CH_2_ piperazine), 3.30–3.42 (m, 6H, 2CH_2_ piperazine, CH_2_CO), 3.70–3.90 (m, 1H, CH cyclopropyl), 7.18–7.20 (m, 2H, ArH), 7.56 (d, 1H, ArH), 7.75 (d, 1H, ArH), 8.65 (s, 1H, C2-H), 10.30, 10.55 (2 s, NH, D_2_O exchangeable), 15.19 (s, 1H, COOH, D_2_O exchangeable) ppm; ^13^C NMR (DMSO-d_6_): *δ* 8.0, 14.6, 19.0, 36.3, 49.8, 56.4, 106.8, 107.1, 111.2, 111.4, 117.6, 119.0, 139.6, 145.5, 148.3, 152.2, 154.7, 166.3, 168.5, 170.3, 176.7 ppm. Anal. Calcd. for C_25_H_25_ClFN_5_O_4_S (546): C, 54.99; H, 4.61; N,12.83. Found: C, 54.92; H, 4.63; N,12.80.

#### General procedure for the preparation of 7-(4-((N'-(4-substituted-2-oxo-2,3-dihydro-1H-indol-3-ylidene)hydrazine carbonyl)methyl)piperazin-1-yl)1-cyclopropyl-6-fluoro-4-oxo-1,4-dihydroquinoline-3-carboxylic acid (8a–b)

A mixture of hydrazinyl derivative **2** (0.50 g, 0.001 mol), substituted isatin (0.001 mol) and glacial acetic acid (1 ml) in absolute ethanol (5 ml) was heated under reflux for 7 h. The reaction mixture was cooled, and the separated solid was filtered, dried, and recrystallised from ethanol to give compounds **8a–b**.

##### 1-Cyclopropyl-6-fluoro-4-oxo-7-(4-(2-oxo-2-(2-(2-oxoindolin-3-ylidene)hydrazinyl)ethyl) piperazin-1-yl)-1,4-dihydroquinoline-3-carboxylic acid (8a)

M.P.: 186–188; yield 85%; IR (KBr) *v*_max_: 3441 (OH), 3240 (NH), 3051 (C–H arom.), 2974 (C–H aliph.), 1730, 1720, 1712 (C = O) cm^−1^. ^1^H NMR (DMSO-d_6_): *δ* 1.02–1.05 (m, 2H, CH_2_ cyclopropyl), 1.17–1.20 (m, 2H, CH_2_ cyclopropyl), 2.72–2.74 (m, 4H, 2CH_2_ piperazine), 3.29–3.31 (m, 4H, 2CH_2_ piperazine), 3.45 (s, 2H, CH_2_CO), 3.75–3.85 (m, 1H, CH cyclopropyl), 6.85–6.88 (m, 1H, ArH), 7.03–7.05 (m, 1H, ArH), 7.36–7.45 (m, 2H, ArH), 7.50 (d, 1H, ArH), 7.90 (d, 1H, ArH), 8.65 (s, 1H, C2-H), 10.96, 11.10 (2 s, 1H, NH, D_2_O exchangeable), 13.84 (s, 1H, NH, D_2_O exchangeable), 15.91 (s, 1H, COOH, D_2_O exchangeable) ppm. ^13^C NMR (DMSO-d_6_): *δ* 8.0, 14.6, 36.3, 49.8, 58.6, 60.3, 106.8, 111.5, 112.6, 118.2, 118.9, 123.2, 125.1, 128.6, 134.8, 138.8, 139.5, 145.6, 151.1, 152.1, 154.6, 166.3, 170.3, 176.7, 184.8 ppm. Anal. Calcd. for C_27_H_25_FN_6_O_5_ (532.52): C, 60.90; H, 4.73; N, 15.78. Found: C, 60.88; H, 4.71; N, 15.76.

##### 7-(4-(2-(2-(4-Bromo-2-oxoindolin-3-ylidene)hydrazinyl)-2-oxoethyl)piperazin-1-yl)-1-cyclopropyl-6-fluoro-4-oxo-1,4-dihydro-quinoline-3-carboxylic acid (8b)

M.P.: 263–265; yield 70%; IR (KBr) *v*_max:_ 3498 (OH), 3200, 3174 (NH), 3078 (C–H arom.), 2916 (C–H aliph.), 1720, 1700, 1697 (C = O) cm^−1^; ^1^H NMR (DMSO-d_6_): *δ* 1.20–1.23 (m, 2H, CH_2_ cyclopropyl), 1.30–1.35 (m, 2H, CH_2_ cyclopropyl), 2.74–2.77 (m, 4H, 2CH_2_ piperazine), 3.34–3.35 (m, 4H, 2CH_2_ piperazine), 3.45 (s, 2H, CH_2_CO), 3.80–3.90 (m, 1H, CH cyclopropyl), 6.85–6.90 (m, 1H, ArH), 7.57–7.65 (m, 2H, ArH), 7.72 (d, 1H, ArH), 7.89 (d, 1H, *J* = 12 Hz, ArH), 8.60 (s, 1H, C2-H), 11.14, 11.26 (2 s, 1H, NH, D_2_O exchangeable), 13.83 (s, 1H, NH, D_2_O exchangeable), 15.21 (s, 1H, COOH, D_2_O exchangeable) ppm. ^13^C NMR (DMSO-d_6_): *δ* 8.0, 14.6, 49.8, 52.0, 58.6, 60.3, 106.9, 107.2, 111.2, 111.5, 114.7, 119.0, 120.0, 127.3, 139.6, 140.4, 145.5, 145.6, 148.4, 150.0, 159.4, 166.3, 170.3, 176.8, 183.6 ppm. Anal. Calcd. for C_27_H_24_BrFN_6_O_5_ (611.42): C, 53.04; H, 3.96; N, 13.75. Found: C, 57.13; H, 4.15; N, 14.18.

#### General procedure for the preparation of 7-(4-(substituted)phenyl)carbamoyl)amino)carbamoyl)methyl)piperazin-1-yl)-1-cyclopropyl-6-fluoro-4-oxo-1,4-dihydroquinoline-3-carboxylic acid (9a-c)

A mixture of hydrazinyl derivative **2** (0.50 g, 0.001 mol), substituted phenyl isocyanate or phenyl isothiocyanate (0.001 mol), and glacial acetic acid (1 ml) in absolute ethanol (15 ml) was heated under reflux for 7 h. The reaction mixture was cooled, and the separated solid was filtered, dried, and recrystallised from ethanol to give compounds **9a–c**.

##### 7-(4-(2-(2-((4-Chloro-3-(trifluoromethyl)phenyl)carbamoyl)-hydrazinyl)-2-oxoethyl) piperazin-1-yl)-1-cyclopropyl-6-fluoro-4-oxo-1,4-dihydroquinoline-3-carboxylic acid (9a)

M.P.: 186–188; yield 65%; IR (KBr) *v*_max:_ 3437 (OH), 3414 (NH), 3055 (C–H arom.), 2908 (C–H aliph.), 1730, 1728 (C = O) cm^−1^; ^1^H NMR (DMSO-d_6_): *δ* 1.04–1.08 (m, 2H, CH_2_ cyclopropyl), 1.32–1.33 (m, 2H, CH_2_ cyclopropyl), 2.65 (s, 2H, CH_2_CO), 2.70–2.77 (m, 4H, 2CH_2_ piperazine), 3.30–3.45 (m, 4H, 2CH_2_ piperazine), 3.80–3.90 (m, 1H, CH cyclopropyl), 7.56–7.70 (m, 3H, ArH), 7.87–8.15 (m, 2H, ArH), 8.65 (s, 1H, C2-H), 9.30 (s, 1H, NH, D_2_O exchangeable), 9.50 (s, 1H, NH, D_2_O exchangeable), 10.10 (s, 1H, NH, D_2_O exchangeable), 15.07 (s, 1H, COOH, D_2_O exchangeable) ppm; ^13^C NMR (DMSO-d_6_): *δ* 8.3, 14.6, 49.8, 52.0, 60.3, 106.8, 106.9, 107.1, 111.2, 111.4, 118.9, 119.0, 139.6, 145.5, 145.6, 148.3, 152.2, 154.7, 166.3, 170.3, 176.7, 176.8 ppm. Anal. Calcd. for C_27_H_25_ClF_4_N_6_O_5_ (624.97): C, 51.89; H, 4.03; N, 13.45. Found: C, 52.17; H, 4.19; N, 13.71.

##### 7-(4-(2-(2-((2-Chloro-6-methylphenyl)carbamoyl)hydrazinyl)-2-oxoethyl)piperazin-1-yl)-1-cyclopropyl-6-fluoro-4-oxo-1,4-dihydro-quinoline-3-carboxylic acid (9b)

M.P.: 285–287; yield 83%; IR (KBr) *v*_max_: 3572 (OH), 3441 (NH), 3051 (C–H arom.), 2904 (C–H aliph.), 1730, 1728 (C = O) cm^−1^. ^1^H NMR (DMSO-d_6_): *δ* 1.15–1.25 (m, 4H, 2CH_2_ cyclopropyl), 2.20–2.25 (m, 11H, 4CH_2_ piperazine, CH_3_), 3.34 (s, 2H, CH_2_CO), 4.80–4.90 (m, 1H, CH cyclopropyl), 7.17–7.24 (m, 3H, ArH), 7.33–7.35 (m, 3H, ArH), 8.94 (brs, 3H, 3NH, D_2_O exchangeable). ^13^C NMR (DMSO-d_6_): *δ* 8.0, 18.8, 36.3, 44.1, 49.8, 49.9, 106.9, 107.8, 111.4, 119.0, 127.7, 129.2, 133.1, 135.6, 139.3, 145.4, 145.5, 148.2, 152.1, 154.4, 155.6, 166.3, 176.6, 176.7 ppm. Anal. Calcd. for C_27_H_28_ClFN_6_O_5_ (571): C, 56.79; H, 4.94; N, 14.72. Found: C, 57.05; H, 5.12; N, 14.98.

##### 1-Cyclopropyl-6-fluoro-7-(4-(2-(2-((4-methoxyphenyl)carbamo-thioyl)hydrazinyl)-2-oxoethyl)piperazin-1-yl)-4-oxo-1,4-dihydroquinoline-3-carboxylic acid (9c)

M.P.: 238–240; yield: 62%; IR (KBr) *v*_max:_ 3500 (OH), 3379 (NH), 3008 (C–H arom.), 2912 (C–H aliph.), 1712 (C = O) cm^−1^; ^1^H NMR (DMSO-d_6_): *δ* 1.17–1.20 (m, 2H, CH_2_ cyclopropyl), 1.33–1.35 (m, 2H, CH_2_ cyclopropyl), 3.40 (s, 2H, CH_2_CO), 3.49–3.50 (m, 4H, 2CH_2_ piperazine), 3.75 (s, 3H, CH_3_O), 3.83–3.84 (m, 1H, CH cyclopropyl), 4.14–4.15 (m, 4H, 2CH_2_ piperazine), 6.82 (d, 2H, *J* = 8.0 Hz, ArH), 7.19 (d, 2H, *J* = 8.0 Hz, ArH), 7.59 (d, 1H, *J* = 8.0 Hz, ArH), 7.90 (d, 1H, *J* = 13.20 Hz, ArH), 8.67 (s, 1H, C2-H), 9.34 (s, 2H, 2NH, D_2_O exchangeable) and 15.16 (s, 1H, COOH, D_2_O exchangeable); ^13^C NMR (DMSO-d_6_): *δ* 8.0, 36.3, 40.4, 47.7, 49.1, 55.6, 106.5, 107.1, 111.3, 111.5, 113.7, 118.8, 118.9, 127.8, 134.1, 145.1, 148.4, 151.9, 157.0, 166.5, 176.7, 182.2 ppm. Anal. Calcd. for C_27_H_29_FN_6_O_5_S (568.62): C, 57.03; H, 5.14; N, 14.78. Found: C, 57.21; H, 5.38; N, 15.02.

#### General procedure for the preparation of 1-cyclopropyl-6-fluoro-7-(4-((N'-(2-(substituted phenyl)-2-oxoethyl)hydrazine carbonyl)methyl)piperazin-1-yl)-4-oxo-1,4-dihydroquinoline-3-carboxylic acid (10a–c)

A mixture of hydrazinyl derivative **2** (0.50 g, 0.001 mol), anhydrous potassium carbonate (0.41 g, 0.003 mol) and substituted phenacyl bromide (0.001 mol) in dry benzene (8 ml) was heated under reflux for 24 h. The reaction mixture was filtered while hot. The residue was washed twice with water (20 ml), dried, and recrystallised from ethanol to give compound **10a–c**.

##### 1-Cyclopropyl-6-fluoro-4-oxo-7-(4-(2-oxo-2-(2-(2-oxo-2-phenyl-ethyl)hydrazinyl)ethyl) piperazin-1-yl)-1,4-dihydroquinoline-3-carboxylic acid (10a)

M.P.: 183–185 °C; yield: 45%; (KBr) *v*_max_: 3500 (OH), 3402 (NH), 3008 (C–H arom.), 2916 (C–H aliph.), 1732, 1700, 1697 (C = O) cm^−1^; ^1^H NMR (DMSO-d_6_): *δ* 1.11–1.18 (m, 2H, CH_2_ cyclopropyl), 1.30–1.32 (m, 2H, CH_2_ cyclopropyl), 2.75–2.79 (m, 2H, CH_2_ piperazine), 2.90–2.95 (m, 2H, CH_2_ piperazine), 3.40 (s, 2H,CH_2_CO), 3.45–3.50 (m, 2H, CH_2_ piperazine), 3.60–3.70 (m, 2H, CH_2_ piperazine), 3.80–3.90 (m, 1H, CH cyclopropyl), 4.00 (s, 2H, CH_2_CO), 5.61 (s, 1H, NH, D_2_O exchangeable), 7.37–8.05 (m, 7H, ArH), 8.56 (s, 1H, C2-H), 8.66 (s, 1H, NH, D_2_O exchangeable), ppm; ^13^C NMR (DMSO-d_6_): *δ* 7.9, 17.0, 26.0, 36.5, 46.2, 50.0, 102.0, 111.0, 116.5, 126.0, 127.4, 128.5, 129.4, 143.0, 144.5, 150.5, 161.0, 166.6, 174.5 ppm. Anal. Calcd. for C_27_H_28_FN_5_O_5_ (521.54): C, 62.18; H, 5.41; N, 13.43. Found: C, 61.96; H, 4.70; N, 13.72.

##### 7-(4-(2-(2-(2-(3-Bromophenyl)-2-oxoethyl)hydrazinyl)-2-oxo-ethyl)piperazin-1-yl)-1-cyclopropyl-6-fluoro-4-oxo-1,4-dihydroquinoline-3-carboxylic acid (10b)

M.P.: 210–215 °C; yield: 62%; IR (KBr) *v*_max_: 3500 (OH), 3240 (NH), 3020 (C–H arom.), 2920 (C–H aliph.), 1725 (C = O) cm^−1^; ^1^H NMR (DMSO-d_6_): *δ* 1.16–1.18 (m, 2H, CH_2_ cyclopropyl), 1.29–1.32 (m, 2H, CH_2_ cyclopropyl), 2.90–3.00 (m, 4H, 2CH_2_ piperazine), 3.22–3.24 (m, 4H, 2CH_2_ piperazine), 3.30 (s, 2H, CH_2_CO), 3.40–3.70 (m, 3H, CH cyclopropyl, CH_2_CO), 7.35–7.40 (m, 1H, ArH), 7.40–7.48 (m, 1H, ArH), 7.50 (d, 1H, *J* = 8 Hz, ArH), 7.81 (m, 5H, 3ArH, 2NH), and 8.62 (s, 1H, C2-H) ppm; ^13^C NMR (DMSO-d_6_): *δ* 8.0, 27.0, 36.2, 45.40, 50.6, 53.0, 106.5, 107.1, 111.1, 111.4, 118.7, 123.7, 124.3, 128.7, 129.3, 135.7, 139.6, 146.0, 148.2, 152.1, 154.6, 166.4, 167.2, 176.7 ppm. Anal. Calcd. for C_27_H_27_BrFN_5_O_5_ (600.45): C, 54.01; H, 4.53; N, 11.66. Found: C, 55.21; H, 4.82; N, 12.03.

##### 1-Cyclopropyl-6-fluoro-7-(4-(2-(2-(2-(4-nitrophenyl)-2-oxoethyl)-hydrazinyl)-2-oxoethyl)piperazin-1-yl)-4-oxo-1,4-dihydroquinoline-3-carboxylic acid (10c)

M.P.: 221–223 °C; yield: 55%; IR (KBr) *v*_max_: 3540 (OH), 3402 (NH), 3008 (C–H arom.), 2950 (C–H aliph.), 1730 (C = O) cm^−1^; ^1^H NMR (DMSO-d_6_): *δ* 1.10–1.15 (m, 2H, CH_2_ cyclopropyl), 1.30–1.35 (m, 2H, CH_2_ cyclopropyl), 2.80–2.85 (m, 4H, 2CH_2_ piperazine), 3.20–3.25 (m, 4H, 2CH_2_ piperazine), 3.30 (s, 2H, CH_2_CO), 3.60 (s, 2H, CH_2_CO), 3.80–3.90 (m, 1H, CH cyclopropyl), 7.36 (s, 1H, NH, D_2_O exchangeable), 7.50–7.60 (m, 2H, ArH), 7.70 (d, 1H, ArH), 8.10–8.50 (m, 3H, ArH), 8.59 (s, 1H, NH, D_2_O exchangeable), and 8.66 (s, 1H, C2-H) ppm; ^13^C NMR (DMSO-d_6_): *δ* 8.0, 29.0, 31.5, 36.0, 45.5, 50.8, 106.5, 108.6, 111.2, 111.4, 119.3, 121.2, 128.2, 129.8, 131.4, 132.2, 139.5, 148.1, 152.1, 154.6, 166.6, 176.4 ppm. Anal. Calcd. for C_27_H_27_FN_6_O_7_ (566.55): C, 57.24; H, 4.80; N, 14.83. Found: C, 57.41; H, 4.98; N, 15.09.

##### Procedure for the preparation of 1-cyclopropyl-7-(4-(2-((2,5-dioxopyrrolidin-1-yl)amino)-2-oxoethyl)piperazin-1-yl)-6-fluoro-4-oxo-1,4-dihydroquinoline-3-carboxylic acid (11)

A mixture of hydrazinyl derivative **2** (0.50 g, 0.001 mol), succinic anhydride (0.10 g, 0.001 mol), and anhydrous sodium acetate (0.117 g, 0.0015 ml) in glacial acetic acid (10 ml) was heated under reflux for 5 h. The reaction mixture was concentrated to half its volume and allowed to cool, the separated solid was filtered, washed with cold ethanol, dried, and recrystallised from acetic acid to give compound **11**. M.P.: 184–186 °C; yield: 55%; IR (KBr) *v*_max_: 3441 (OH), 3224 (NH), 3016 (C–H arom.), 2947 (C–H aliph.), 1720, 1712, 1700 (C = O) cm^−1^; ^1^H NMR (DMSO-d_6_): *δ* 1.18–1.20 (m, 2H, CH_2_ cyclopropyl), 1.31–1.33 (m, 2H, CH_2_ cyclopropyl), 2.24 (s, 4H, 2CH_2_ pyrrolidine), 2.70–2.80 (m, 4H, 2CH_2_ piperazine), 3.10 (s, 2H, CH_2_CO), 3.30–3.40 (m, 4H, 2CH_2_ piperazine), 3.83–3.84 (m, 1H, CH cyclopropyl), 7.56 (d, 1H, *J* = 8 Hz, ArH), 7.90 (d, 1H, *J* = 13.6 Hz, ArH), 8.66 (s, 1H, C2-H), and 9.68 (s, 1H, NH, D_2_O exchangeable) ppm. ^13^C NMR (DMSO-d_6_): *δ* 8.0, 19.5, 20.8, 26.6, 28.7, 29.8, 106.6, 107.3, 111.2, 111.4, 119.0, 139.5, 145.5, 148.2, 152.1, 154.6, 168.4, 176.6 ppm. C_23_H_24_FN_5_O_6_ (485.46): C, 56.90; H, 4.98; N, 14.43. Found: C, 56.87; H, 4.95; N, 14.41.

##### Procedure for the preparation of 1-cyclopropyl-7-(4-(2-((1,3-dioxoisoindolin-2-yl)amino)-2-oxoethyl)piperazin-1-yl)-6-fluoro-4-oxo-1,4-dihydroquinoline-3-carboxylic acid (12)

A mixture of compound 2 (0.50 g, 0.001 mol), phthalic anhydride (0.15 g, 0.001 mol), and anhydrous sodium acetate (0.117 g, 0.0015 ml) in glacial acetic acid (10 ml) was heated under reflux for 5 h. The reaction mixture was concentrated to half its volume and allowed to cool, the separated solid was filtered, washed with cold ethanol, dried, and recrystallised from acetic acid to give compound **12**. M.P: 190–192 °C; yield: 50%; IR (KBr) *v*_max_: 3441 (OH), 3224 (NH), 3012 (C–H arom.), 2897 (C–H aliph.), 1720, 1700 (C = O) cm^−1^; ^1^H NMR (DMSO-d_6_): *δ* 1.10–1.15 (m, 2H, CH_2_ cyclopropyl), 1.20–1.30 (m, 2H, CH_2_ cyclopropyl), 2.65–2.75 (m, 4H, 2CH_2_ piperazine), 3.11 (s, 2H, CH_2_CO), 3.25–3.35 (m, 4H, 2CH_2_ piperazine), 3.75–3.80 (m, 1H, CH cyclopropyl), 7.36–8.15 (m, 6H, ArH), 8.63 (s, 1H, C2-H), and 9.74 (s, 1H, NH, D_2_O exchangeable) ppm. Anal. Calcd. for C_27_H_24_FN_5_O_6_ (533.51): C, 60.78; H, 4.53; N, 13.13. Found: C, 60.92; H, 4.58; N, 13.11.

##### Procedure for the preparation of 7-(4-(2-(2-carbamothioylhydrazinyl)-2-oxoethyl) piperazin-1-yl)-1-cyclopropyl-6-fluoro-4-oxo-1,4-dihydroquinoline-3-carboxylic acid (13)

A mixture of hydrazinyl derivative **2** (0.50 g, 0.001 mol), potassium thiocyanate (0.194 g, 0.002 mol), and concentrated hydrochloric acid (1 ml) in absolute ethanol (15 ml) was heated under reflux for 6 h. The reaction mixture was cooled, and the separated solid was filtered, dried, and recrystallised from ethanol to give compound **13**. M.P.: 188–200 °C; yield: 65%; IR (KBr) *v*_max_: 3417 (OH), 3271, 3236 (NH, NH_2_), 3001 (C–H arom.), 2947 (C–H aliph.), 1747, 1728 (C = O) cm^−1^; ^1^H NMR (DMSO-d_6_): *δ* 1.19–1.21 (m, 2H, CH_2_ cyclopropyl), 1.34–1.35 (m, 2H, CH_2_ cyclopropyl), 3.30–3.50 (m, 8H, 4CH_2_ piperazine), 3.67 (s, 2H, CH_2_CO), 3.87–3.89 (m, 1H, CH cyclopropyl), 7.20 (s, 1H, NH, D_2_O exchangeable), 7.30 (s, 2H, NH_2_, D_2_O exchangeable), 7.40 (s, 1H, NH, D_2_O exchangeable), 7.63 (d, 1H, *J* = 8 Hz, ArH), 7.96 (d, 1H, *J* = 13 Hz, ArH), 8.68 (s, 1H, C2-H), and 15.20 (s, 1H, COOH, D_2_O exchangeable) ppm; ^13^C NMR (DMSO-d_6_): *δ* 8.0, 14.5, 17.0, 51.8, 56.1, 107.2, 130.0, 144.3, 148.4, 148.5, 152.0, 152.1, 154.5, 166.2, 166.3, 168.5, 176.7 ppm. Anal. Calcd. for C_20_H_23_FN_6_O_4_S (462.5): C, 51.94; H, 5.01; N, 18.17 Found: C, 51.91; H, 5.12; N, 18.22

##### Procedure for the preparation of 1-cyclopropyl-6-fluoro-4-oxo-7-(4-(2-oxo-2-(2-(4-oxothiazolidin-2-ylidene)hydrazinyl)ethyl)piperazin-1-yl)-1,4-dihydroquinoline-3-carboxylic acid (14)

A mixture of compound **13** (0.23 g, 0.0005 mol), ethyl chloroacetate (0.60 g, 0.005 mol) in absolute ethanol (15 ml) was heated under reflux for 6 h. The obtained precipitate was filtered, washed with water, dried, and recrystallised from ethanol to form compound **14**. MP: 240–242 °C; yield: 70%; IR (KBr) *v*_max_: 3417 (OH), 3271 (NH), 3020 (C–H arom.) 2947(C–H aliph.), 1720 (C = O) cm^−1^; ^1^H NMR (DMSO-d_6_): *δ* 1.18–1.20 (m, 2H, CH_2_ cyclopropyl), 1.34–1.36 (m, 2H, CH_2_ cyclopropyl), 2.60–2.80 (m, 4H, 2CH_2_ piperazine), 3.28 (s, 4H, 2CH_2_CO), 3.30–3.35 (m, 4H, 2CH_2_ piperazine), 3.51 (brs, 2H, 2NH, D_2_O exchangeable), 3.80–3.90 (m, 1H, CH cyclopropyl), 7.58 (d, 1H, *J* = 8.0 Hz, ArH), 7.90 (d, 1H, *J* = 13 Hz, ArH), 8.66 (s, 1H, C2-H), and 15.21 (s, 1H, COOH, D_2_O exchangeable) ppm. ^13^C NMR (DMSO-d_6_): *δ* 6.2, 21.4, 29.3, 44.6, 52.6, 60.5, 106.4, 111.2, 114.5, 121.0, 144.5, 144.6, 156.0, 165.5, 170.2, 178.1, 188.2 ppm. Anal. Calcd. for C_22_H_23_FN_6_O_5_S (502.52): C, 52.58; H, 4.61; N, 16.72. Found: C, 52.79; H, 4.75; N, 16.88.

##### Procedure for the preparation of 1-cyclopropyl-6-fluoro-4-oxo-7-(4-(2-oxo-2-(2-(4-phenylthiazol-2(3H)-ylidene)hydrazinyl)ethyl)piperazin-1-yl)-1,4-dihydroquinoline-3-carboxylic acid (15)

A mixture of compound **13** (0.18 g, 0.0004 mol), phenacyl bromide (0.08 g, 0.0004 mol), and anhydrous sodium acetate (0.03 g, 0.0004 mol) in absolute ethanol (15 ml) was heated under reflux for 6 h. The obtained precipitate was filtered, washed with water, dried, and recrystallised from ethanol to form compound **15**. M.P.: 256–258 °C; yield: 25%; IR (KBr) *v*_max_: 3549 (OH), 3317 (NH), 3089 (C–H arom.) 2912 (C–H aliph.), 1728 (C = O) cm^−1^; ^1^H NMR (DMSO-d_6_): *δ* 1.19–1.20 (m, 2H, CH_2_ cyclopropyl), 1.33–1.35 (m, 2H, CH_2_ cyclopropyl), 2.21–2.23 (m, 4H, 2CH_2_ piperazine), 3.36–3.38 (m, 4H, CH_2_CO and 2NH), 3.70–3.75 (m, 4H, 2CH_2_ piperazine), 3.80–3.90 (m, 1H, CH cyclopropyl), 7.15–7.20 (m, 3H, ArH), 7.35 (d, 2H, ArH), 7.55 (d, 1H, *J* = 8.0 Hz, ArH), 7.85 (d, 1H, *J* = 13.0 Hz, ArH), 8.31 (s, 1H, thiazole-H), 8.62 (s, 1H, C2-H), and 14.80 (s, 1H, COOH, D_2_O exchangeable) ppm. ^13^C NMR (DMSO-d_6_): *δ* 8.0, 18.8, 36.3, 44.2, 49.9, 106.9, 107.1, 111.5, 119.1, 119.2, 127.2, 127.7, 129.3, 133.2, 135.6, 139.3, 145.5, 148.3, 152.1, 154.6, 155.7, 166.3, 176.7 ppm. Anal. Calcd. for C_28_H_27_FN_6_O_4_S (562.18): C, 59.78; H, 4.84; N, 14.94. Found: C, 59.50; H, 5.22; N, 14.84.

### Biological activity

#### Cell culture protocol

Human bladder cancer (T-24) and human prostate carcinoma (PC-3) cell lines were obtained from American Type Culture Collection (ATCC; Manassas, VA USA). Cells were maintained in Dulbecco’s modified Eagle medium with 10% foetal bovine serum at 37 °C and 5% CO_2_. All the operations were carried out under strict aseptic conditions. The culture medium was removed to a centrifuge tube containing 9.0 ml complete culture medium and spanned at approximately 125 × *g* for 5 to 7 min. The cell layer was rinsed with 0.25% (w/v) trypsin- 0.53 µM EDTA solution by which all traces of serum-containing inhibitor were removed. Trypsin EDTA solution 2.0 to 3.0 ml was added to a flask and cells were monitored under an inverted microscope until the cell layer was dispersed (usually within 5–15 min). A complete growth medium was added (6.0–8.0 ml), and cells were aspirated by gentle pipetting. The cell pellet was suspended with the recommended complete medium and dispensed into a 75 cm^2^ culture flask. The culture vessel containing the complete growth medium was placed in the incubator for 24 h to at 37 °C. The cells were treated with different concentrations (0.39, 1.60, 6.25, 25, and 100 μg/ml) of each of the test compounds or doxorubicin, followed by incubation for 48 h at 37 °C, then the plates were examined under the inverted microscope and finally the MTT assay was carried out.

#### Cell viability assay

Anti-cancer activity of the newly synthesised compounds was evaluated *in vitro* against both T-24 and PC-3 cell lines according to the MTT method^62^. Cells were seeded into 96-well plates (flat bottom) at a density of 10 000 cells/well for 24 h. The vial of MTT to be used was reconstituted with 3 ml of medium or balanced salt solution without phenol red and serum. then, a reconstituted MTT vial was added in an amount equal to 10% of the culture medium volume. Cultures were incubated for 2–4 h depending on the cell type and maximum cell density and removed from the incubator and the formazan crystals were dissolved via the addition of an amount of MTT solubilisation solution (M-8910) equal to the original culture medium volume. ROBONIK P2000 spectrophotometer at a wavelength of 570 nm was used to measure the colour intensity spectrophotometrically. The survival curve of both T-24 and PC-3 cells was obtained by plotting the percentage of surviving cells against the drug after each compound. The IC_50_ value for each test compound and the reference drug doxorubicin was calculated.

#### In vitro DNA topo II-mediated relaxation assay

DNA Topo II inhibitory activities of the targeted compounds were measured as follows. A mixture of 100 ng of supercoiled pBR322 plasmid DNA (Fermentas, USA) and 2 units of human DNA Topo II (USB Corp., USA) was incubated with and without the prepared compounds in the assay buffer (10 µM Tris-HCl pH 7.9) containing 50 µM NaCl, 4 µM MgCl_2_, 1 µM EDTA, 1 µM ATP, and 15 mg/ml bovine serum albumin for 30 min at 37 °C. The reaction in a final volume of 10 µl was terminated by the addition of 4 µl of 6 µM EDTA. DNA samples were then electrophoresed on 1% agarose gel containing 0.5 µg/ml ethidium bromide in gel and buffer at high voltage (100–250 v) until the dye front has migrated about 4–6 cm down the gel. with a running buffer of TAE Tris-acetate– EDTA (TAE). Gels were stained for 15 min in water. DNA bands were visualised by transillumination with UV light and quantitated using AlphaI-maker TM (Alpha Innotech Corporation).

#### Cell cycle analysis of compounds 6 and 8a

The cell cycle was done using a propidium iodide flow cytometry kit (Abcam, ab139418) according to the manufacturer’s instructions. T-24 and PC-3 cells were treated with IC_50_ of compounds **6** and **8a** for 24 h. After treatment, the cells were washed twice with ice-cold phosphate buffer saline, then centrifuged and fixed using ice-cold 66% (v/v) ethanol, washed with phosphate buffer saline re-suspended with 0.10 mg/ml, and stained with 200 µl propidium iodide. Cells were analysed by flow cytometry. The cell cycle distributions were calculated using cell–quest software (Bectopn Dickinson).

#### Apoptosis determination

Apoptosis was evaluated using Annexin V fluorescein isothiocyanate (FITC) and propidium iodide (PI) using the Annexin V-FITC/PI apoptosis detection kit (Biovision, Mountain View, CA # K101-25). According to the manufacturer’s instructions after staining the cells with annexin V fluorescein (FITC) isothiocyanate. Briefly, 1–5 × 10^5^ cells were exposed to compounds **6** and **8a** at their IC_50_ concentrations for 24 h. Cells were centrifuged and washed once with serum-containing media followed by resuspension in 500 µl of binding buffer. Then 5 µl Annexin V-FITC and 5 µl of (PI 50 mg/ml) were added and incubated for 5 min at room temperature in the dark. Analyses were performed using a flow cytometer (EX = 488 nm: Em = 530 nm) using a FITC signal detector and PI staining by a phycoerythrin emission signal detector.

#### Measurement of the effect of compounds 6 and 8a on the level of caspase-3 protein (a marker of apoptosis)

The level of the apoptotic marker caspase-3 was measured using The Invitrogen Caspase-3 (active) Human ELISA Kit. The procedure of the used kit was done according to the manufacturer’s instructions.

#### Molecular docking study

Molecular docking studies were performed using the Molecular Operating Environment (MOE, 2014.0901) software. All minimisations were performed using MOE with MMFF94x force field, and the partial and formal charges were calculated automatically. The X-ray crystallographic structures of topoisomerase IIα co-crystallised with DNA^63^ were downloaded from the protein data bank^64^. To prepare the enzyme for the docking study, first DNA chains and water molecules were removed, and then the protonate 3D protocol in MOE with default options was used. For the docking protocol, the triangle matcher placement method and London dG scoring function were used. Validation of the docking results was carried out by docking the reference topoisomerase inhibitor merbarone and comparing the results with a previously reported study. The MOE-validated setup was used to predict the binding interactions and affinity of the synthesised compounds at the active site.

## Results and discussion

### Chemistry

**Scheme 1.**
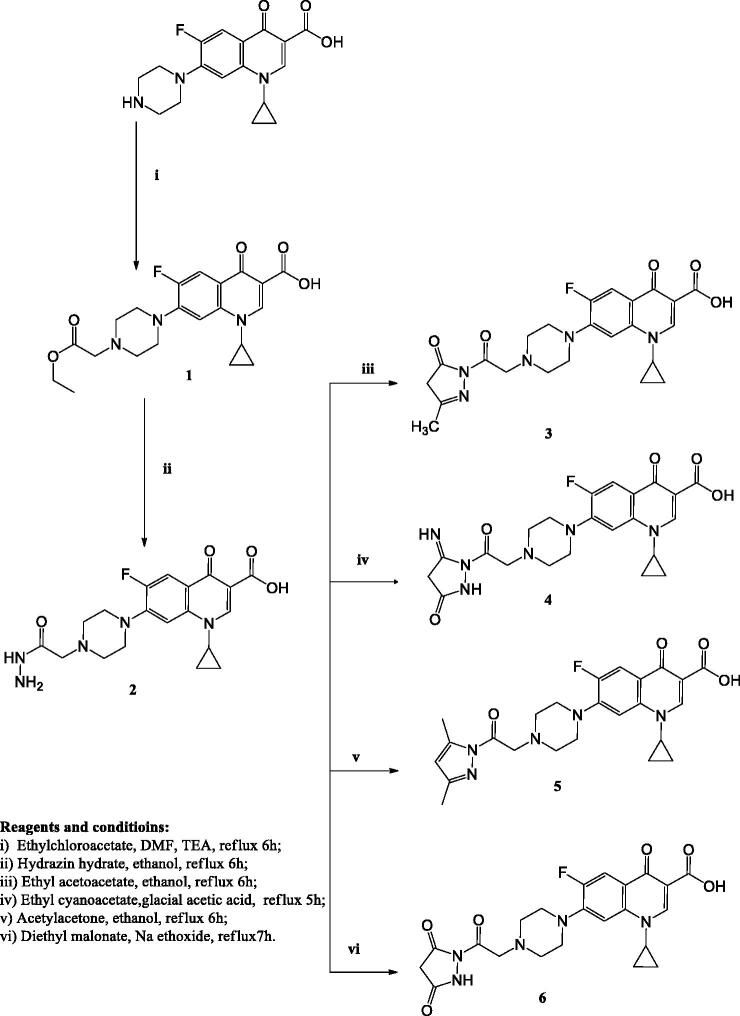
The synthetic path and reagents for the preparation of the target compounds **1**–**6**.

**Scheme 2.**
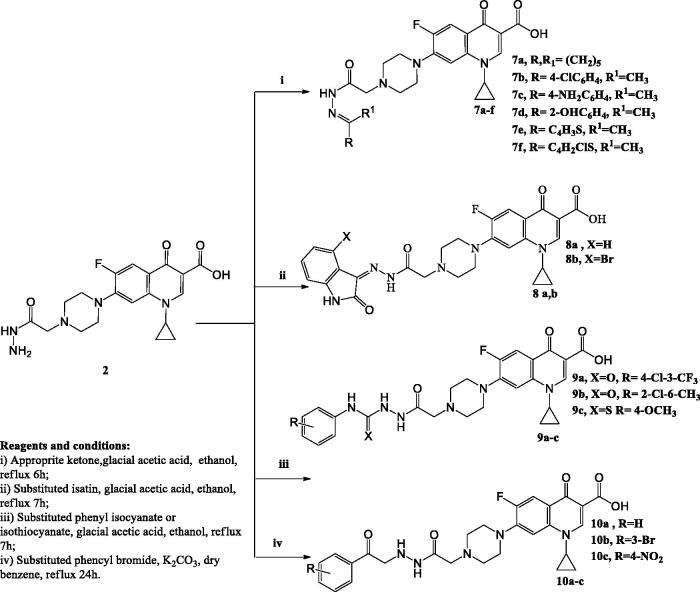
The synthetic path and reagents for the preparation of the target compounds **7–10**.

**Scheme 3.**
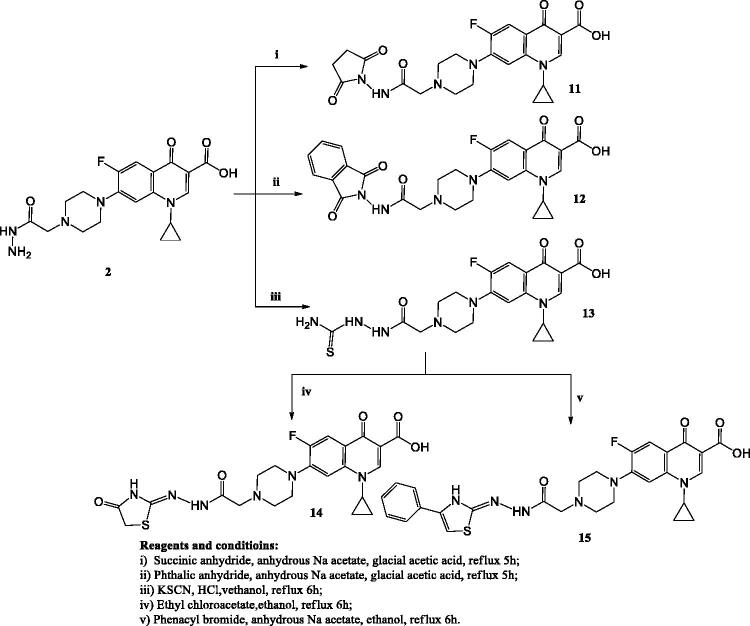
The synthetic path and reagents for the preparation of the target compounds **11–15**.

The synthetic approaches designed in this study for the synthesis of the target compounds are elucidated in Schemes 1–3. The primary starting compound **1**, was prepared by reacting CP with ethyl chloroacetate in dimethylformamide in the presence of trimethylamine^39^. Compound **1** was reacted with hydrazine hydrate to afford compound **2**^39^.

Compound **3** was prepared by reacting hydrazinyl derivative **2** with ethyl acetoacetate in absolute ethanol. The ^1^H NMR spectrum of this compound displayed the disappearance of the signal corresponding to NH_2_ of the parent compound **2** and the appearance of two singlet signals at *δ* 2.47 and 3.80 ppm corresponding to CH_3_C=N and CH_2_ pyrazolone protons, respectively.

CP derivative **4** was prepared by reacting compound **2** with ethyl cyanoacetate in glacial acetic acid, the ^1^H NMR spectrum displayed a singlet signal at *δ* 3.86 ppm expressing the CH_2_ pyrazolone protons. Compound **5** was obtained via refluxing CP hydrazinyl derivative **2** with acetylacetone in ethanol. The ^1^H NMR spectrum displayed the disappearance of the signal corresponding to NH_2_ of the parent compound **2** in addition to the presence of one singlet signal at *δ* 7.90 ppm corresponding to CH of pyrazole.

Compound **6** was obtained by reacting compound **2** with diethyl malonate in the presence of sodium ethoxide. The ^1^H NMR spectrum of this compound revealed the appearance of a singlet signal at *δ* 2.90 ppm due to the CH_2_ of pyrazolone.

In Scheme 2, the hydrazones **7a–f** were obtained by reacting compound **2** with the appropriate ketone in ethanol in the presence of glacial acetic acid. The ^1^H NMR and ^13^C NMR spectra of these compounds displayed the presence of signals corresponding to different alkyl or aryl groups which were not present in compound **2**.

Refluxing compound **2** with the suitable isatin in absolute ethanol in the presence of glacial acetic acid afforded compounds **8a–b**. The ^1^H NMR and ^13^C NMR spectra of these compounds revealed the presence of different signals of indoline moieties.

Compounds **9a–c** were prepared via reacting compound **2** with the appropriate phenyl isocyanates or phenyl isothiocyanate in absolute ethanol in the presence of glacial acetic acid. The ^1^H NMR and ^13^C NMR spectra of these derivatives displayed the characteristic signals corresponding to different aryl moieties. Further structural evidence stemmed from the ^1^H NMR spectra that showed the exchangeable singlet signals corresponding to NH protons.

CP derivatives **10a–c** were prepared via reacting compound **2** with the suitable phenacyl bromide in dry benzene in the presence of potassium carbonate. The ^1^H NMR and ^13^C NMR spectra of these derivatives showed the appearance of new signals corresponding to the added phenyl ring in addition to singlet signals that appeared at *δ* 3.30–3.40 ppm due to CH_2_CO protons.

In Scheme 3, the reaction of compound **2** with succinic anhydride or phthalic anhydride in glacial acetic acid in the presence of anhydrous sodium acetate yielded compounds **11** and **12**, respectively. The ^1^H NMR and ^13^C NMR spectra of these compounds showed the appearance of new signals corresponding to pyrrolidine and isoindoline moieties.

Reacting compound **2** with potassium thiocyanate and concentrated hydrochloric acid in absolute ethanol afforded compound **13**. ^13^C NMR spectrum revealed the appearance of (C = S) carbon at *δ* 176.7 ppm. Compound **14** was prepared via reacting derivative **13** with ethyl chloroacetate in ethanol. The ^1^H NMR spectrum of compound **14** revealed the disappearance of the exchangeable signal of NH_2_ protons.

Finally, compound **15** was prepared by refluxing compound **13** with phenacyl bromide in absolute ethanol in the presence of anhydrous sodium acetate. The ^1^H NMR and ^13^C NMR spectra showed the expected signals corresponding to the phenyl group, which were not present in the starting compound **14**. Additionally, the ^1^H NMR spectrum revealed the appearance of a single signal corresponding to CH of the thiazole ring at *δ* 8.31 ppm.

### Biological activity

#### Growth inhibition against human tumour cell lines

In this study, all the newly synthesised CP derivatives were subjected to anticancer activity evaluation against bladder (T-24) and prostate cancer (PC-3) cell lines. The compounds were evaluated for their activity using 5 doses determinations (100 μg/ml, 25 μg/ml, 6.25 μg/ml, 1.60 μg/ml, and 0.39 μg/ml). Their half-maximal inhibitory concentration (IC_50_) values were measured. Doxorubicin was chosen as a reference anticancer drug^65^.

The test compounds showed anticancer activity against the T-24 cell line with IC_50_ values ranging from 3.36–366 μM. They exhibited IC_50_ values against the PC-3 cell line in the range of 3.25–159.24 μM (Table 1).

**Table 1.** The half-maximal inhibitory concentration (IC_50_) of CP derivatives and doxorubicin after treatment for 24 h on both the prostate cancer cell line (PC-3) and bladder cancer cell line (T-24).

Compound	IC_50_ (μM* ± SD)	
	T-24	PC-3
**3**	37.32 ± 2.02	98.20 ± 5.00
**4**	**25.46 ± 1.38**	29.89 ± 1.53
**5**	**25.90 ± 1.39**	24.94 ± 1.26
**6**	**5.68 ± 0.3**	92.16 ± 4.69
**7a**	**3.88 ± 0.21**	**9.35 ± 0.48**
**7b**	**11.50 ± 0.63**	**19.04 ± 0.96**
**7c**	**25.59 ± 0.71**	32.77 ± 0.86
**7d**	88.02 ± 4.75	**8.81 ± 0.44**
**7e**	**25.00 ± 0.2**	50.49 ± 10.36
**7f**	124.27 ± 6.7	136.26 ± 6.94
**8a**	**3.36 ± 0.19**	**10.95 ± 0.56**
**8b**	336.10 ± 18.15	41.05 ± 2.09
**9a**	**4.35 ± 0.24**	67.17 ± 3.42
**9b**	**28.55 ± 1.54**	**19.33 ± 0.98**
**9c**	134.01 ± 7.23	**4.85 ± 0.27**
**10a**	45.44 ± 2.45	74.31 ± 3.78
**10b**	**17.23 ± 0.93**	**3.38 ± 0.18**
**10c**	**10.08 ± 0.55**	**3.25 ± 0.167**
**11**	68.47 ± 3.69	82.21 ± 4.18
**12**	47.00 ± 1.09	125.58 ± 6.39
**13**	34.51 ± 1.86	30.53 ± 1.56
**14**	**19.48 ± 0.58**	**6.49 ± 0.24**
**15**	**15.92 ± 0.87**	**7.54 ± 0.39**
**Doxorubicin**	29.11 ± 1.56	23.07 ± 1.18

*The results given are the means of three experiments.

Bold values indicate that these CP derivatives are more potent than Doxorubicin.

**Figure 4.**
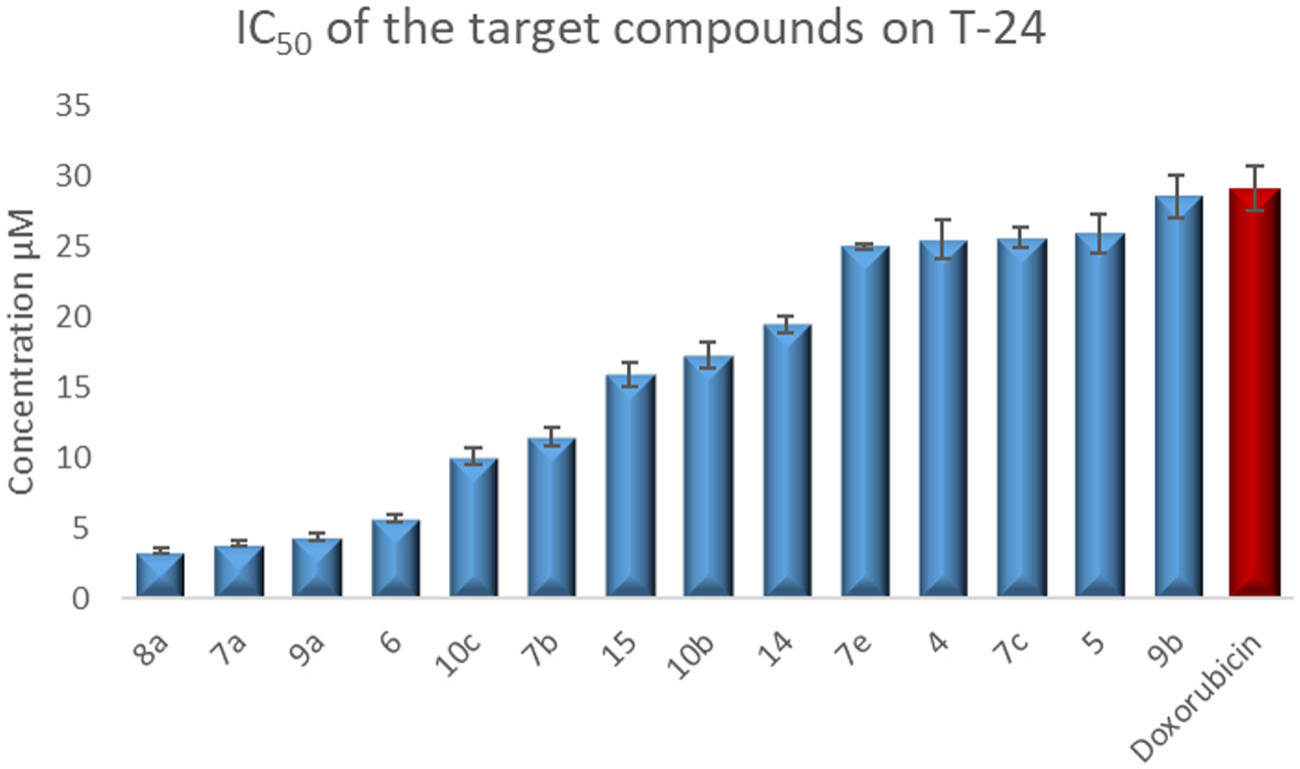
Graphical representation for the half-maximal inhibitory concentration (IC_50_) of CP derivatives and doxorubicin after treatment for 24 h on bladder cancer cell line.

Compounds **4–6**, **7a–c**, **7e**, **8a**, **9a–b**, **10b–c**, **14**, and **15** showed 1.02- to 8.66-fold more potent anti-proliferative activity than the reference standard doxorubicin against T-24 cell line (Figure 4).

**Figure 5.**
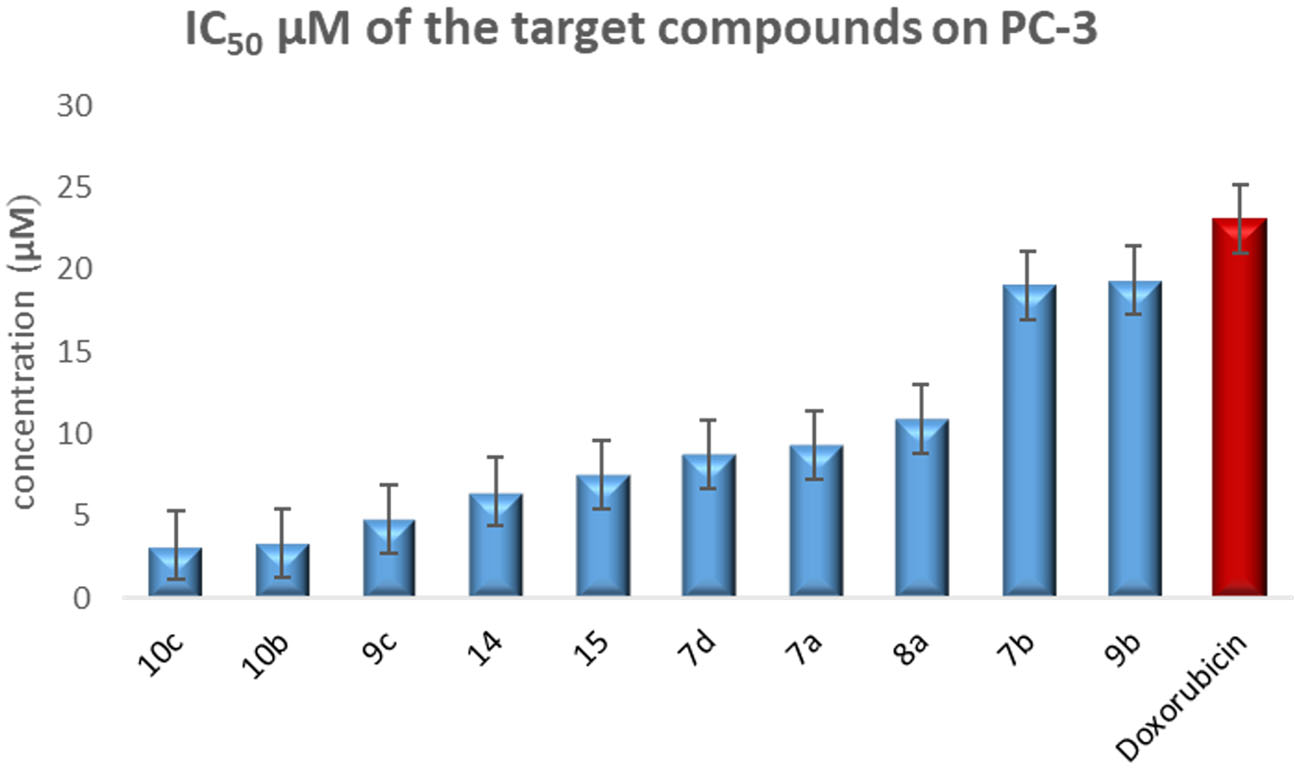
Graphical representation for half-maximal inhibitory concentration (IC_50_) of the target compounds and doxorubicin after treatment for 24 h on the prostate cancer cell line.

Compounds **7a–b**, **7d**, **8a**, **9b–c**, **10b–c**, **14**, and **15** exhibited 1.2- to 7.1-fold more potent anti-proliferative activity than doxorubicin against PC-3 cells line (Figure 5).

Regarding the activity towards bladder cancer, compounds **6**, **7a**, **7b**, **8a**, **9a**, and **10c** were the most potent among the synthesised CP derivatives.

It is worth mentioning that the T-24 bladder cancer cell line was more sensitive to the synthesised CP derivatives than the PC-3 prostate cancer cell line.

The Structure-activity correlation of the newly synthesised CP derivatives revealed that modification at the piperazinyl N-4 position of the CP scaffold resulted in variable potency. CP derivatives bearing pyrazole ring through acetyl spacer (**4–6**) showed potent anticancer activity. They showed more potency against bladder cancer cell line, compound **6** incorporating pyrazolidine-3,5-dione moiety was the most prominent among them. Regarding CP hydrazones **7a–e**, compound **7a** incorporating cyclohexylidene moiety showed marked potency against bladder and prostate cancer cell lines. Further analysis of these compounds revealed that hydrazones carrying substituted phenyl ring (**7b–d**) were more potent than their counterparts having thiophene ring (**7e**,**f**). An interesting phenomenon is that CP hydrazone **7d** featuring ortho hydroxyl group on the phenyl ring showed the most potent anti-proliferative activity among CP aryl hydrazones against prostate cancer. CP derivative **8a** having the acylhydrazone scaffold with a benzene ring carrying a nearby NH proved to marked anti-proliferative activity against both cell lines. It was clear that the introduction of a bromine atom on the indoline scaffold (**8b**) had a bad impact on the anticancer activity. CP semicarbazide and thiosemicarbazide derivatives **9a–c** possessed potent activity. It was noticed that compound **9a** featuring the 4-chloro-3-trifluoromethylphenyl moiety group was the most potent. Reviewing compounds **10a–c**, revealed that the substitution of phenyl moiety with electron withdrawing group in **10b** and **10c** significantly improved the anticancer activity against the two cell lines. The incorporation of monocyclic pyrrolidine or pyrrolidine fused with benzene in CP derivatives **11** and **12** resulted in lower anticancer activity. It is worth mentioning that, grafting thiazole moiety in derivatives **14** and **15** highly improved the antiproliferative activity.

#### Recombinant topoisomerase II inhibitory activities of compounds (6, 7a, 7b, 8a, 9a, and 10c)

**Figure 6.**
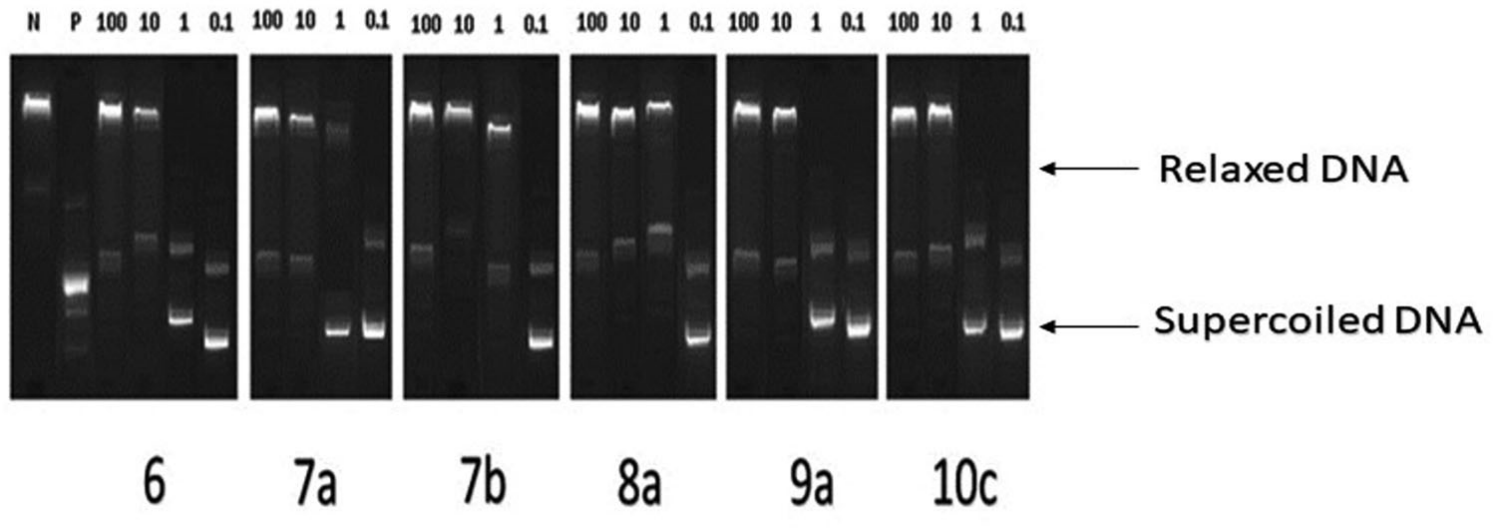
Recombinant Topo II inhibitory activities of compounds **6**, **7a**, **7b**, **8a**, **9a**, and **10c** at 100, 10, 1, 0.1 µM. Lane P: pBR322 DNA only; lane N: pBR322 DNA + Topo II; lanes (**6**, **7a**, **7 b**, **8a**, **9a** and **10c**): pBR322 DNA + Topo II + compounds **6**, **7a**, **7 b**, **8a**, **9a** and **10c**.

The conversion of supercoiled plasmid DNA to relaxed DNA by recombinant Topo II was examined in the presence of each of the most potent compounds **6**, **7a**, **7b**, **8a**, **9a**, and **10c** for measuring their Topo II inhibitory activities. Well-known Topo II inhibitor doxorubicin was used as a positive control. The reaction products of Topo II relaxation assays were analysed by electrophoretic mobility and developed in ethidium bromide in the presence of UV light. The inhibitory activities were evaluated at 4 concentrations of 100, 10, 1, and 0.1 μM for all compounds as shown in (Figure 6). Topo II inhibitory activities of the tested compounds are summarised in (Table 2).

**Table 2.** Recombinant Topo II inhibitory activities of compounds (**6**, **7a**, **7b**, **8a**, **9a**, and **10c**).

Compounds	Percentage of inhibition*			
	100 μM	10 μM	1 μM	0.1 μM
**6**	87.4	76.3	51.9	28.0
**7a**	83.6	55.9	27.9	13.7
**7b**	85.2	60.3	34.0	18.4
**8a**	91.6	77.4	50.3	32.5
**9a**	86.5	57.0	40.3	23.6
**10c**	90.2	72.6	37.7	24.8

*Percentage inhibition of Topo II activity = (Intensity of sample-treated DNA/Intensity of vehicle-treated control DNA) × 100.

All test compounds showed significant Topo II inhibitory activity in the range of 83–90% at 100 μM concentration. While at 0.1 μM concentration, the range of Topo II inhibition was 13.7–32.5% (Table 2). The IC_50_ value of each compound was calculated. Compounds **6**, **8a**, and **10c** were 1.01- to 2.32-fold more potent than doxorubicin (Table 3).

**Table 3.** Topo II IC_50_ results of compounds (**6**, **7a**, **7b**, **8a**, **9a**, and **10c**) compared to doxorubicin.

Compound	IC_50_ (µM*±SD)
**6**	0.92 **±** 0.05
**7a**	4.99 **±** 0.27
**7b**	3.34 **±** 0.18
**8a**	0.74 **±** 0.04
**9a**	2.58 **±** 0.14
**10c**	1.69 **±** 0.09
**Doxorubicin**	1.72 **±** 0.08

*The results given are the means of three experiments.

#### Cell cycle analysis and detection of apoptosis

The most prominent compounds **6** and **8a** that showed Topo II IC_50_ values in the sub-micro-molar range were further investigated in terms of their effects on apoptosis induction and cell cycle progression in both T-24 and PC-3 cell lines.

**Figure 7.**
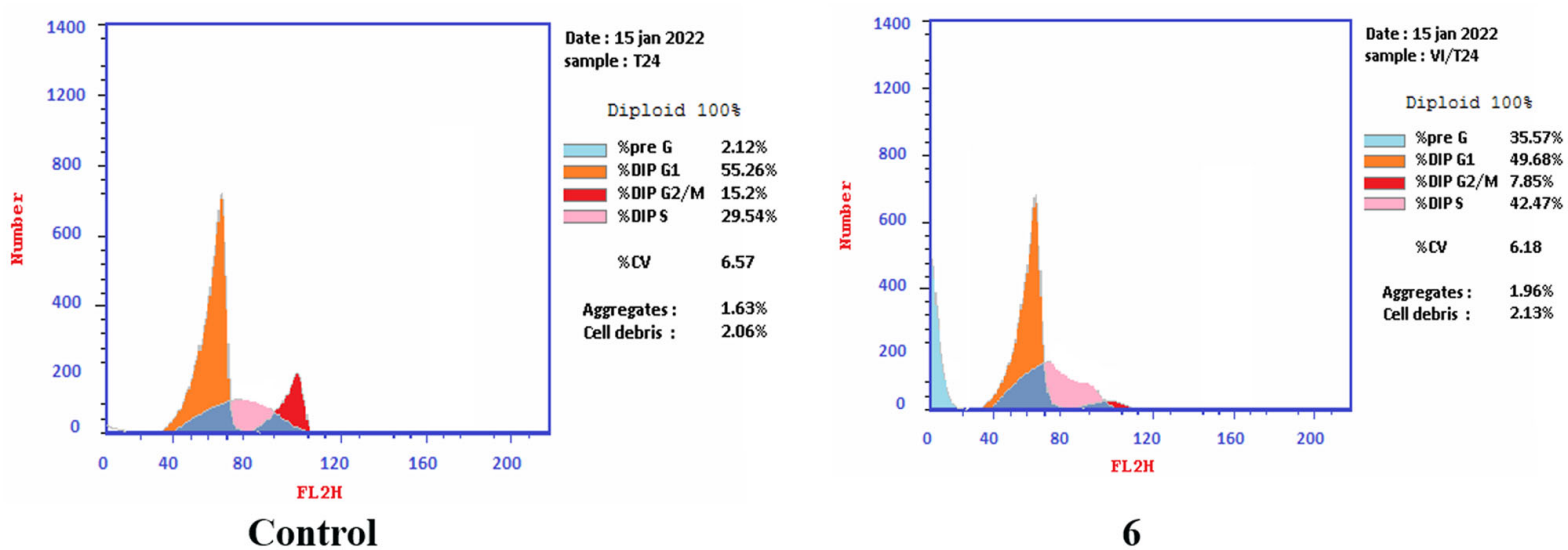
Effect of compound **6** (5.68 μM) on DNA-ploidy flow cytometric analysis of T-24 cells after 24 h.

**Figure 8.**
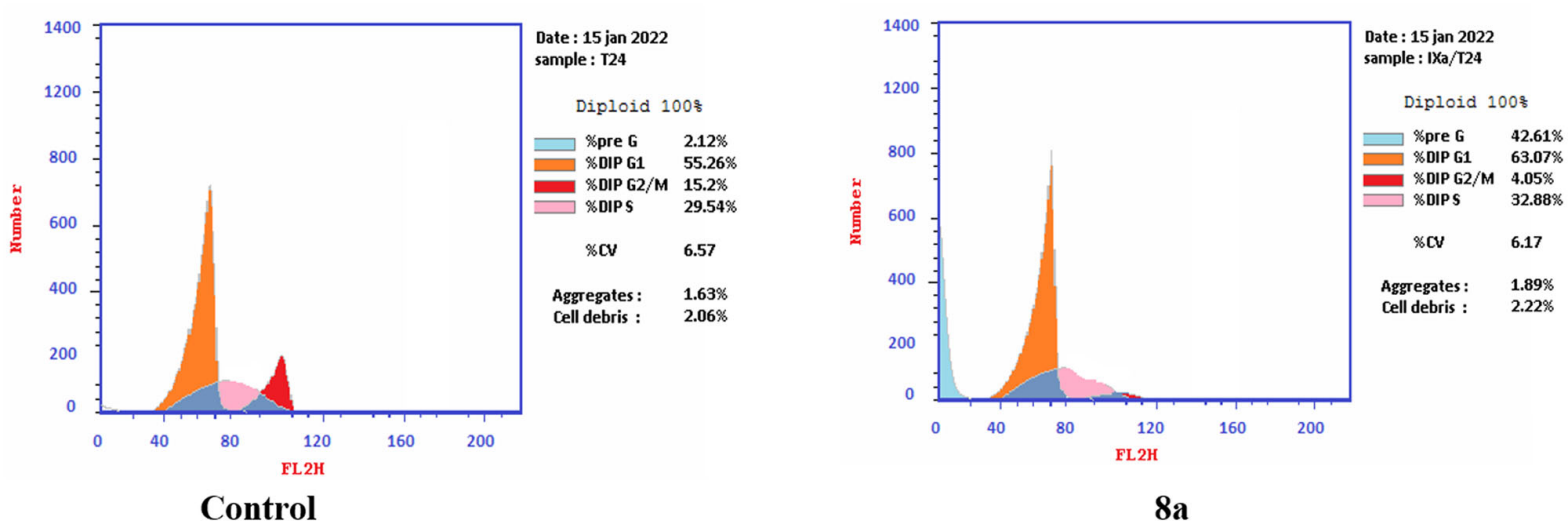
Effect of compound **8a** (3.36 μM) on DNA-ploidy flow cytometric analysis of T-24 cells after 24 h.

**Figure 9.**
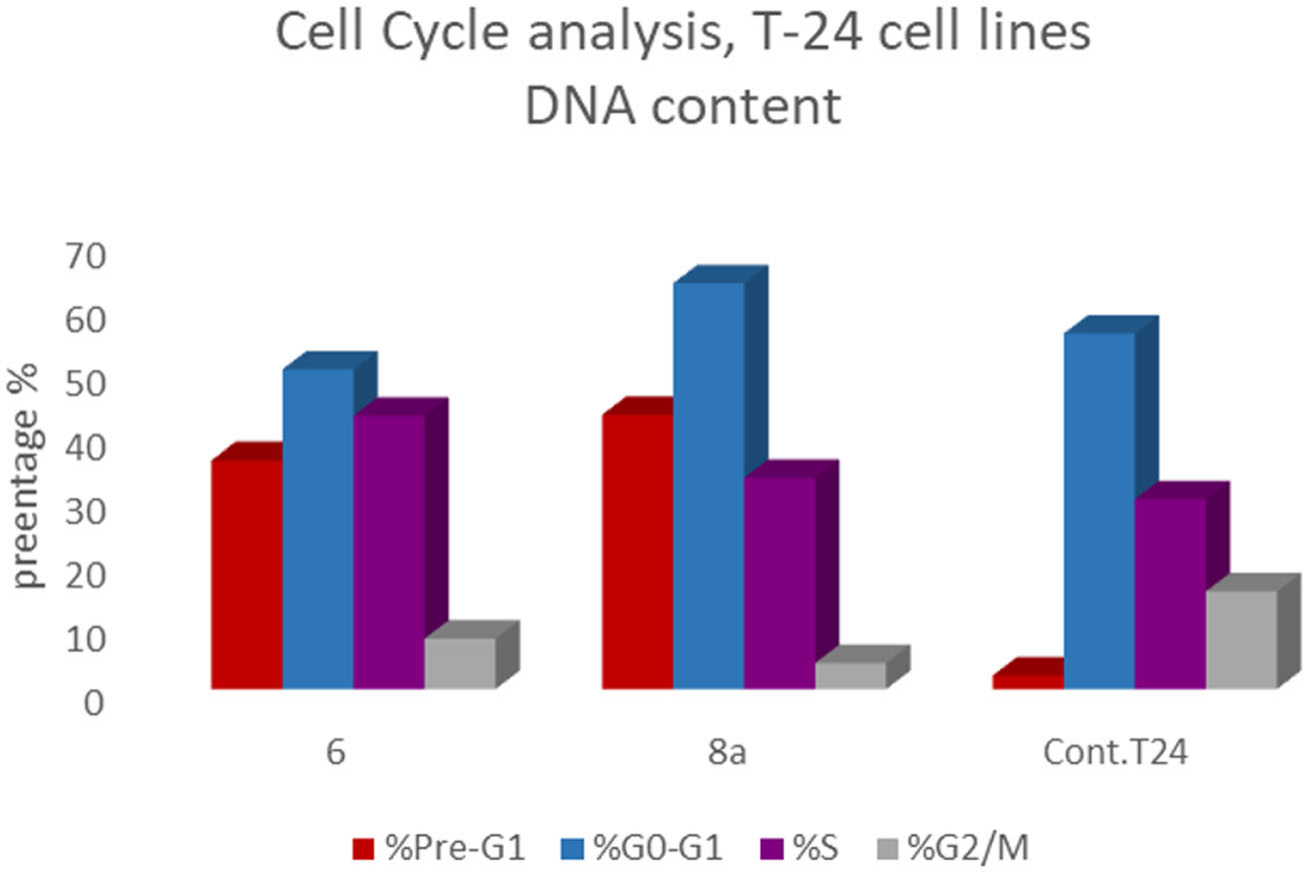
Bar presentation showing effects of compounds **6** (5.68 μM) and **8a** (3.36 μM) on DNA-ploidy flow cytometric analysis of T-24 cells after 24 h.

T-24 and PC-3 cells were treated with compounds **6** and **8a** at their IC_50_ values for 24 h and their effects on the normal cell cycle profile and induction of apoptosis were recorded. Treatment of T-24 and PC-3 cells with compounds **6** and **8a** resulted in an interference with the normal distribution of the cell cycle. In T-24 cell lines, both compounds **6** and **8a** induced a significant increase in the percentage of cells at pre-G_1_ by 16.7- and 20.1-fold, respectively when compared to the control. Also, they showed an increase in the percentage of cells in the S phase by 1.4- and 1.11-fold, sequentially compared to the control (Figures 7–9). The accumulation of cells in the pre-G1 phase indicated that CP derivatives **6** and **8a** induced tumour cell death and cytotoxicity via apoptosis. While the accumulation of the cells in the S phase might result from the cell cycle arrest of T-24 cells in the S phase.

**Figure 10.**
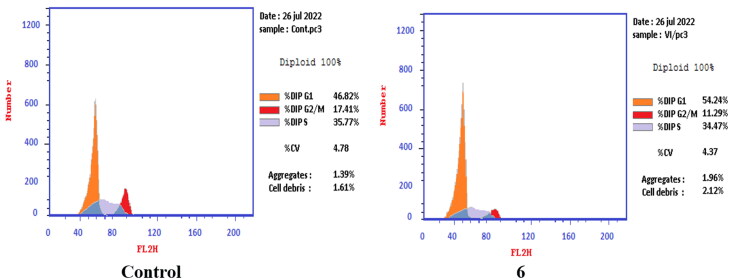
Effect of compound **6** (92.16 μM) on DNA ploidy flow cytometric analysis of PC-3 cells after 24 h.

**Figure 11.**
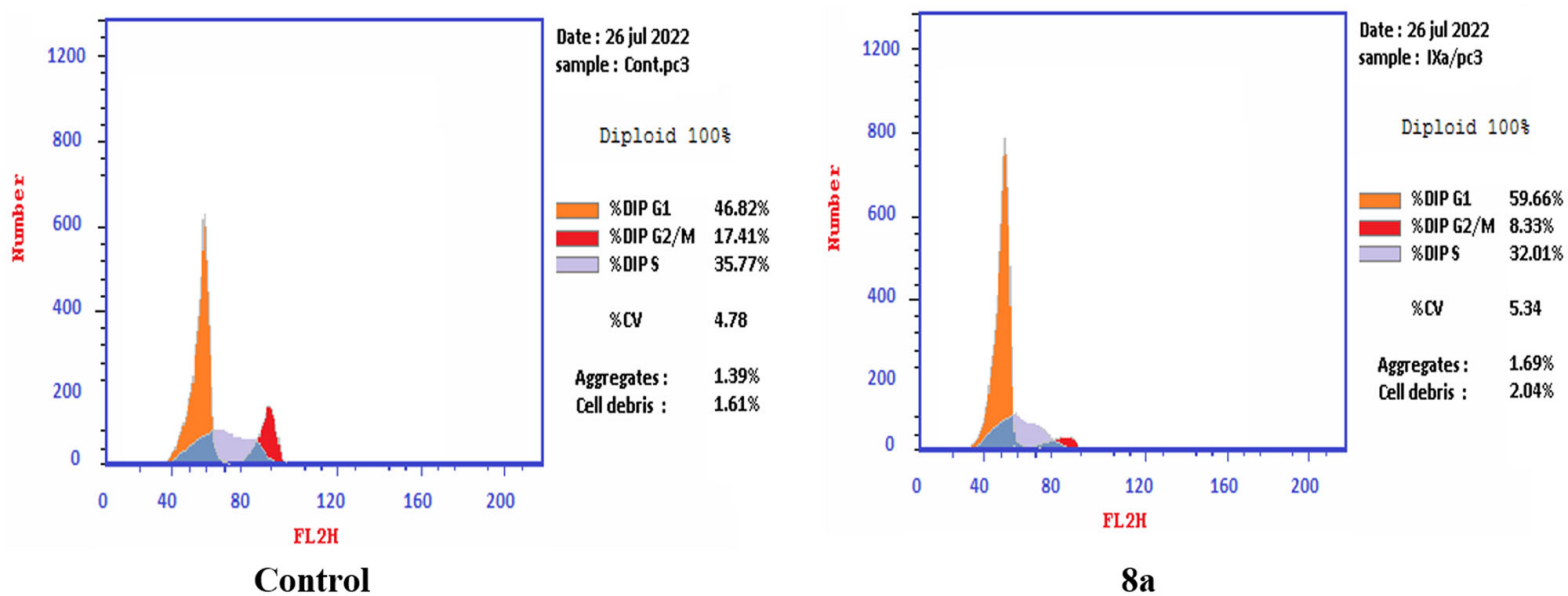
Effect of compound **8a** (10.95 μM) on DNA-ploidy flow cytometric analysis of PC-3 cells after 24 h.

**Figure 12.**
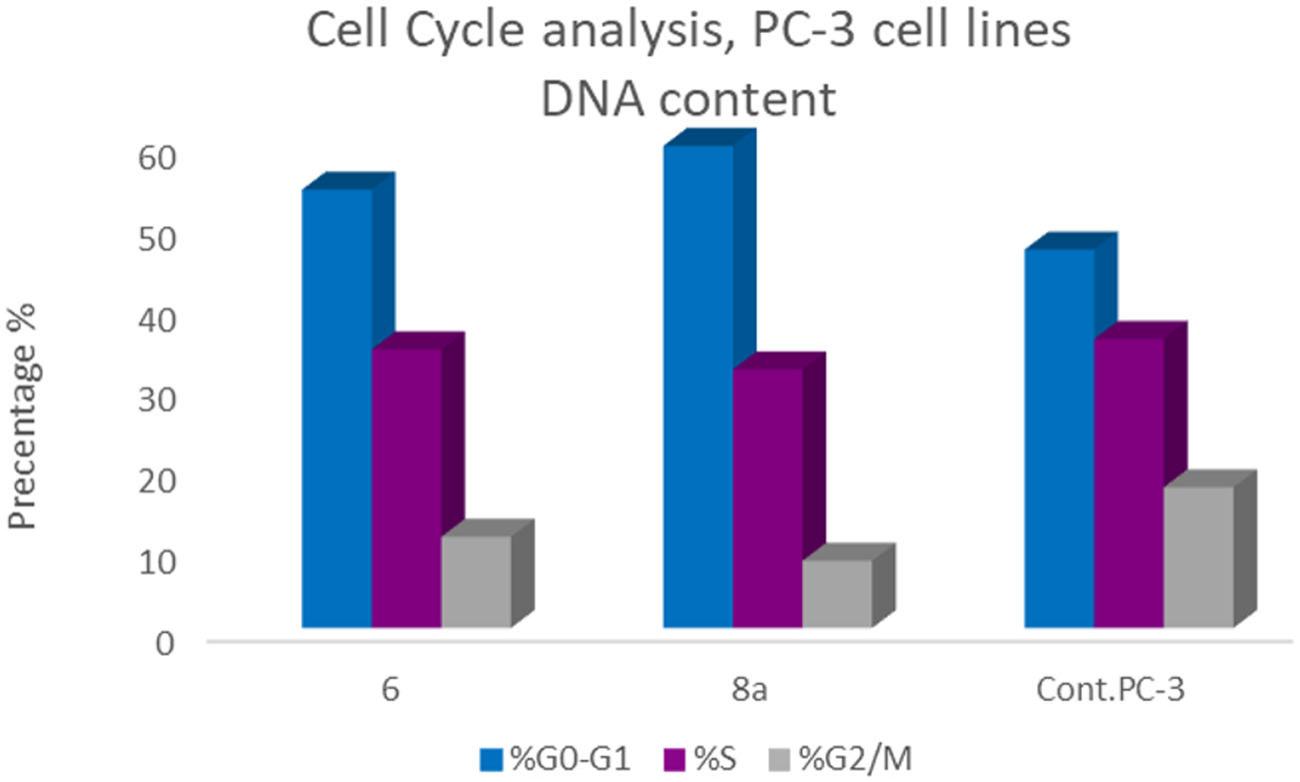
Bar presentation showing effects of compounds **6** (92.16 μM) and **8a** (10.95 μM) on DNA-ploidy flow cytometric analysis of PC-3 cells after 24 h.

In PC-3 cell lines, both compounds **6** and **8a** induced an increase in the percentage of cells at the G1 phase by 1.16- and 1.27-fold, respectively when compared to the control (Figures 10–12). This was confirmed by a concomitant decrease in the percentage of cells in the G2/M phase, where both compounds **6** and **8a** induced a decrease in the percentage of cells at the G2/M phase by 1.5- and 20.1-fold, respectively. Compounds **6** and **8a** induced G1-phase cell cycle arrest of PC-3 cells at their IC_50_ concentrations. Topoisomerase inhibitors cause DNA damage which is related to G1/S and S phase arrest. Cell cycle arrest prevents the replication of damaged DNA. In PC-3 cell line compounds **6** and **8a** probably arrested the cell cycle in the G1 phase by inhibition of Cyclin D–Cdk4–Cdks complex, however in the T-24 cell line, they might arrest the cell cycle in the S phase by the inhibition of both Cyclin D–Cdk4–Cdks complex and cyclins E1–E2/Cdk2, which promote G1/S transition^66–69^.

Similar effects regarding cell cycle arrest at different phases by CP derivatives have been reported, which approved that CP is a promising framework for anticancer drug design and discovery. In 2018, two CP derivatives induced a significant increase in the percentage of cells at pre-G1and G2/M phases by 13.9-, 8.8-fold, and 2.05-, 2.06-fold, respectively compared to the negative control in the UO-31 cell line^39^.

In 2021, Abdel-Rahman et al. reported that a Mannich base CP derivative induced an increase in the percentage of cells at pre-G1and G2/M phases by 17.9- and 4.1-fold, respectively, in the OVCAR-3 cell line compared to the negative control^70^.

#### Apoptosis determination by annexin V-FITC assay

**Figure 13.**
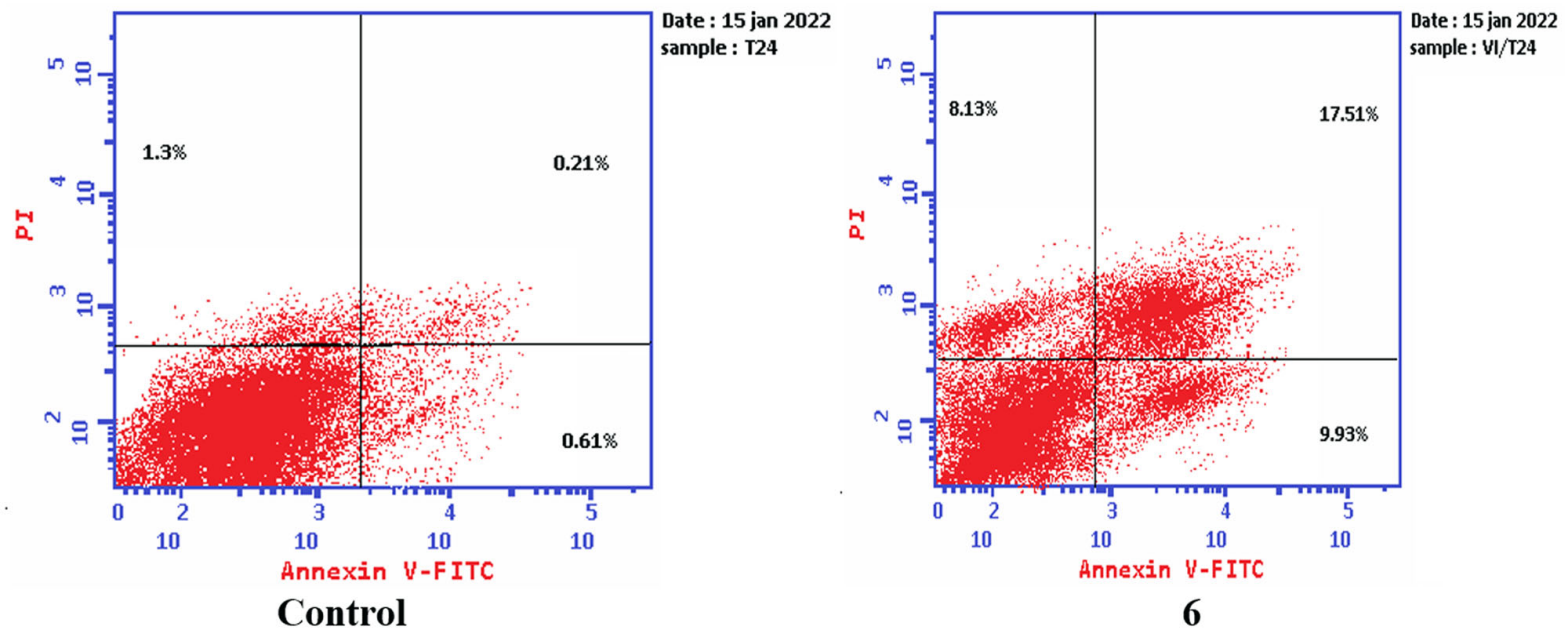
Representative dot plots of T-24 cells treated with **6** (5.68 μM) for 24 h and analysed by flow cytometry after double staining of the cells with annexin-V FITC and PI.

**Figure 14.**
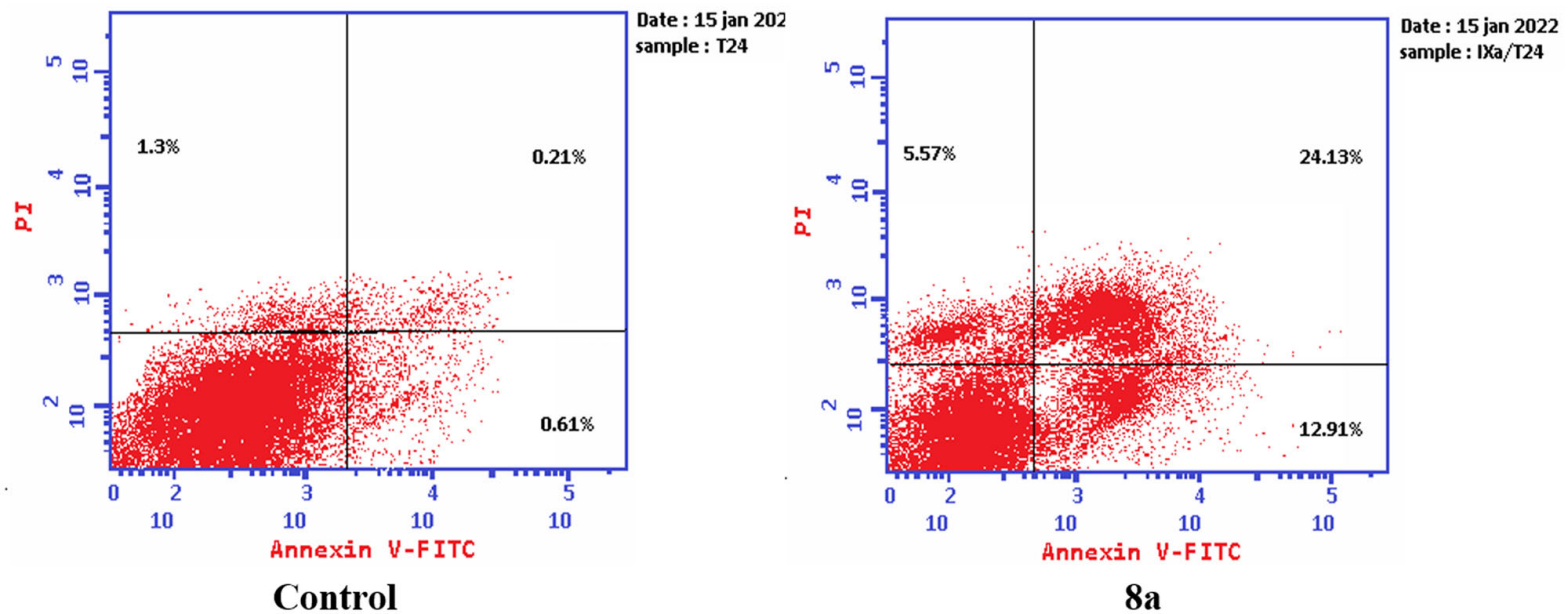
Representative dot plots of T-24 cells treated with **8a** (3.36 μM) for 24 h and analysed by flow cytometry after double staining of the cells with annexin-V FITC and PI.

To verify the ability of compounds **6** and **8a** to induce apoptosis, a flow cytometry assay was performed using propidium iodide (PI), and immunofluorescent markers of the protein annexin-V. Dual staining for annexin-V and with PI provides the distinction between viable cells, early apoptotic cells, late apoptotic cells, and necrotic cells^71–73^. Treatment of T-24 cells with compounds **6** and **8a** at their IC_50_ concentrations resulted in a decrease in the percentage of viable cells. The results showed that compound **6** induced both early and late apoptosis with 16.27- and 83.38-fold more than the control, respectively, and induced total apoptosis and necrosis with 16.77- and 6.25-fold more than the control, respectively (Figure 13). While compound **8a** induced both early and late apoptosis with 21.16- and 114.90-fold more than the control, respectively, and induced total apoptosis and necrosis with 20.1- and 4.28-fold more than the control, sequentially (Figure 14).

**Figure 15.**
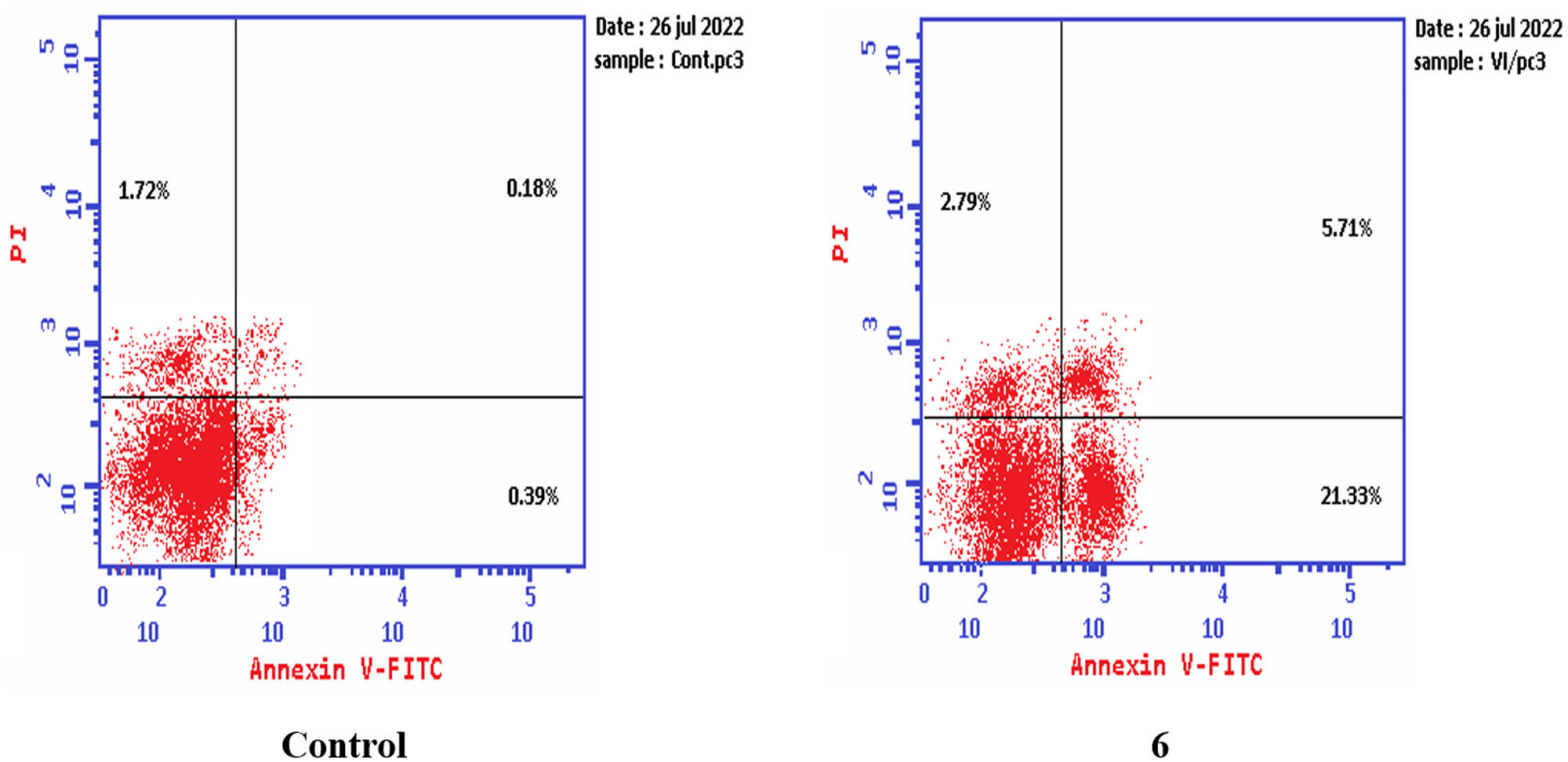
Representative dot plots of PC-3 cells treated with **6** (92.16 μM) for 24 h and analysed by flow cytometry after double staining of the cells with annexin-V FITC and PI.

**Figure 16.**
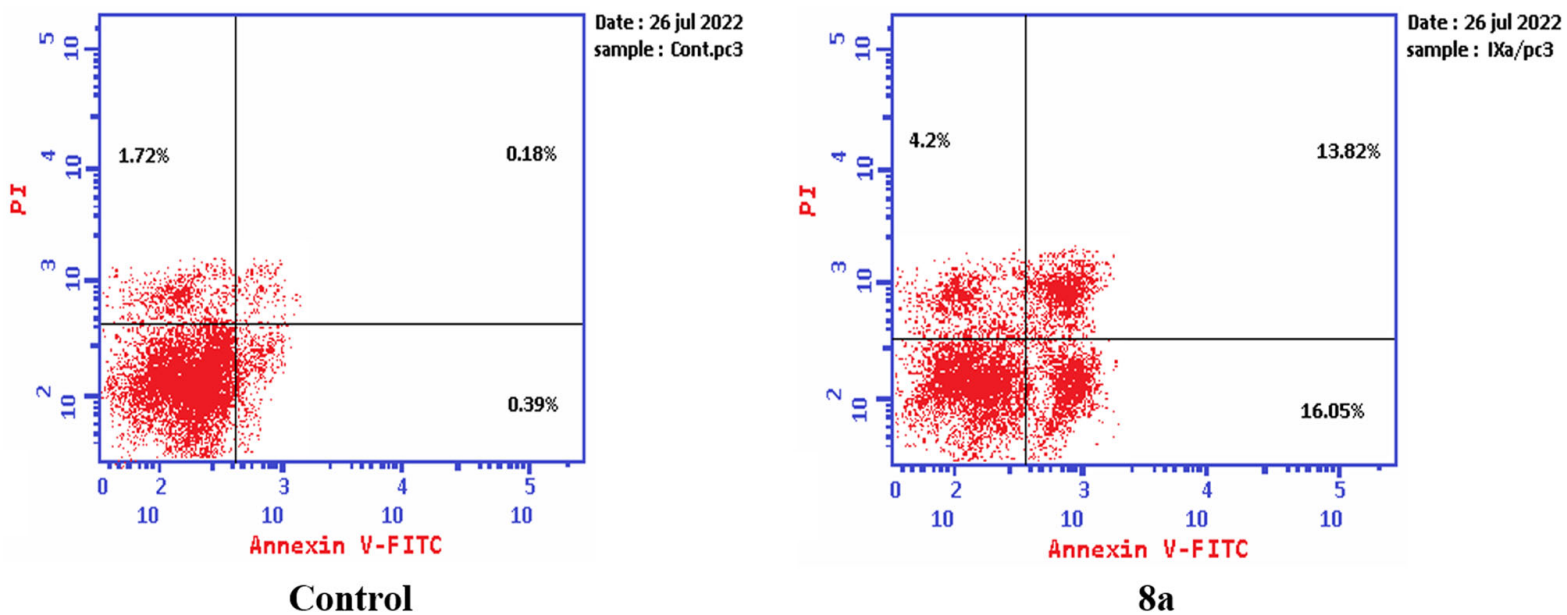
Representative dot plots of PC-3 cells treated with **8a** (10.95 μM) for 24 h and analysed by flow cytometry after double staining of the cells with annexin-V FITC and PI.

While treatment of PC-3 cells with compounds **6** and **8a** at their IC_50_ concentrations resulted in a decrease in the percentage of viable cells. The results showed that compound **6** induced both early and late apoptosis with 54.69- and 31.72-fold more than the control, respectively, and induced total apoptosis and necrosis with 13.02- and 1.62-fold more than the control, respectively (Figure 15). While compound **8a** induced both early and late apoptosis with 41.15- and 76.77-fold more than the control, respectively, and induced total apoptosis and necrosis with 14.87- and 2.44-fold more than the control, sequentially (Figure 16).

#### Effect of compounds 6 and 8a on the level of active caspase-3 (key executioner of apoptosis)

Caspase-3 (a key effector enzyme) plays an important role in apoptosis since its activation leads to catalysing specific enzymes responsible for DNA fragmentation, which leads to cell death^74–77^. Apoptosis induction in T-24 cells by compounds **6** and **8a** was investigated via caspase 3, compared to doxorubicin as a reference drug.

**Figure 17.**
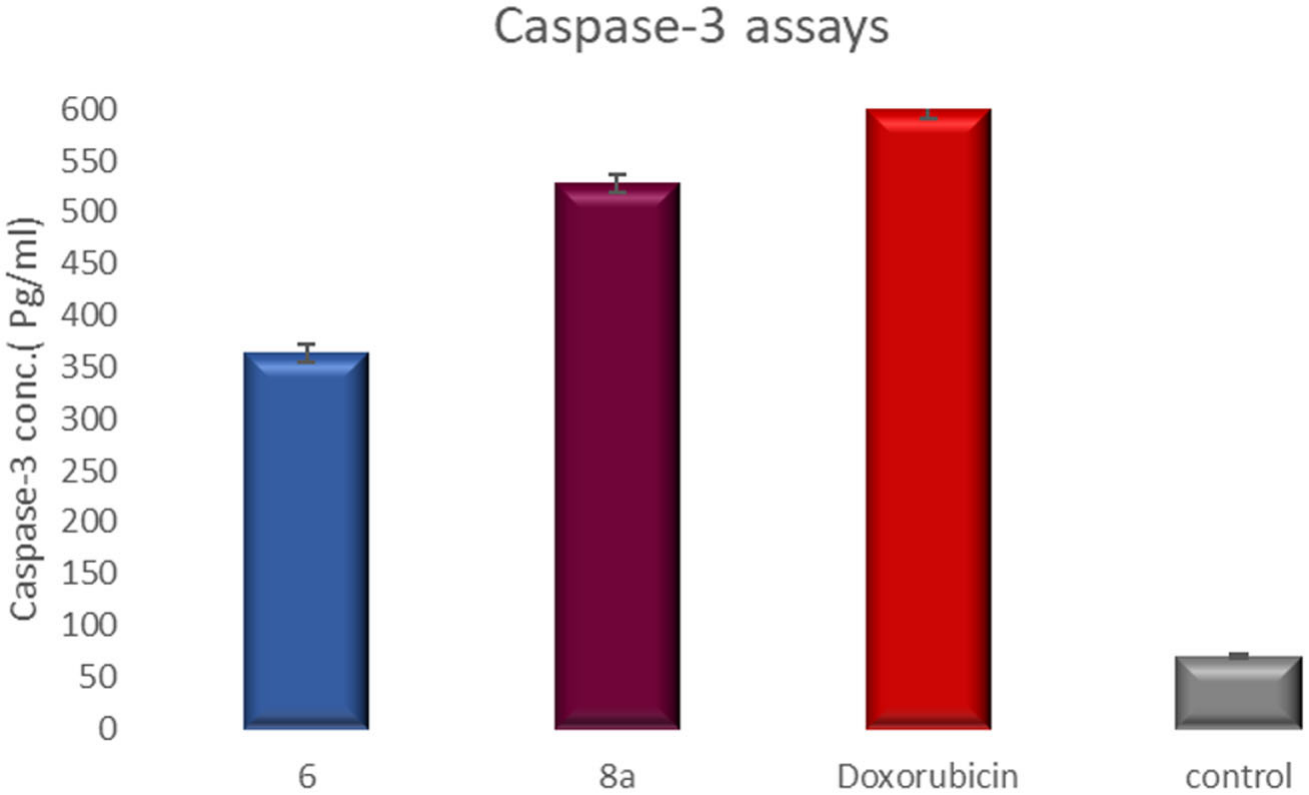
Graphical representation for active caspase-3 assays of compounds **6** and **8a** compared to doxorubicin as a positive control.

Treatment of T-24 cells with compounds **6** and **8a** at concentrations 5.68 and 3.36 μM, sequentially enhanced the level of active caspase-3 compared to the control (5.23- and 7.6-fold) (Table 4, Figure 17).

**Table 4.** Active caspase-3 assay results.

Compound	Caspase-3 conc. (pg/ml* ± SD)
**6**	362.9 ± 8.58
**8a**	527.6 ± 8.39
**Doxorubicin**	598.8 ± 8.63
**Control**	69.36 ± 1.70

*The results given are the means of three experiment.

#### Molecular docking study

**Figure 18.**
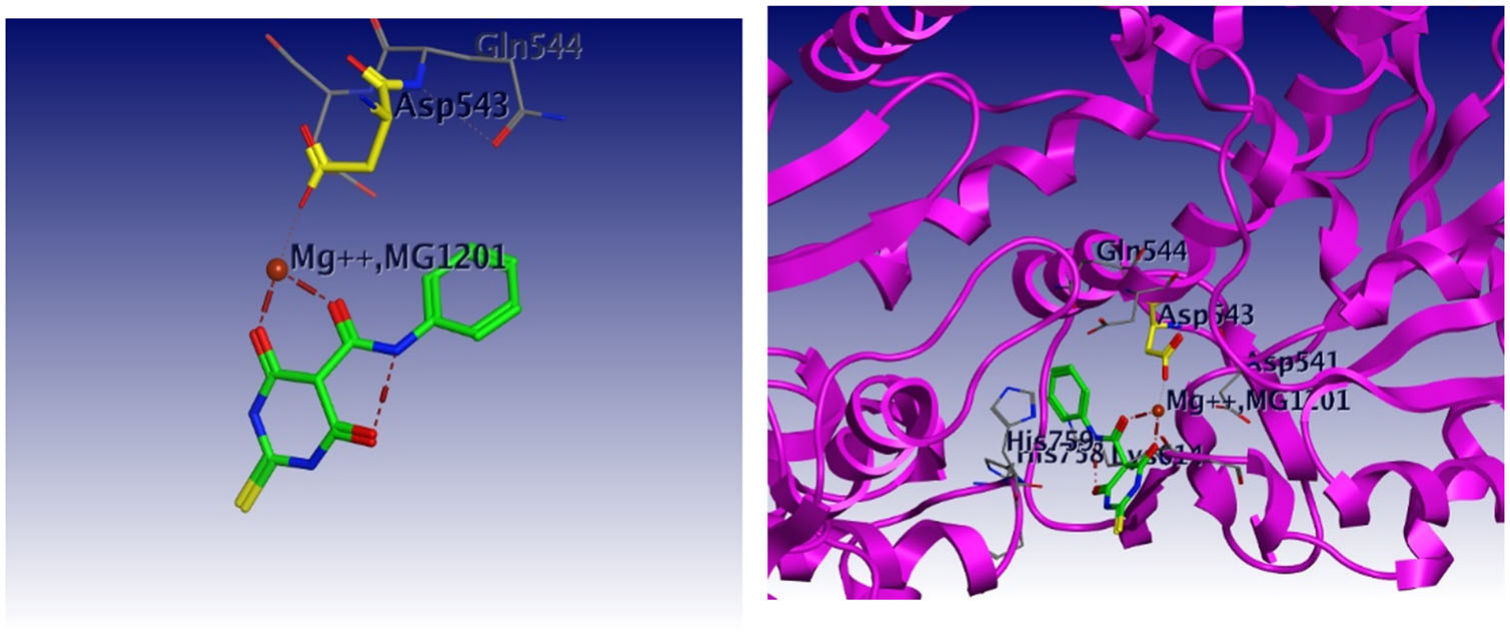
3D interaction of merbarone with DNA binding site of topoisomerase IIα. Red dashed lines represent coordinate bond interactions with Mg^2+^. Red tiny, dashed lines are hydrogen bonding interactions with amino acid Asp 543. Mg2^+^ is shown as a nonbonded sphere (crimson red). Residues that are involved in hydrogen bonding are shown in the stick presentation.

**Figure 19.**
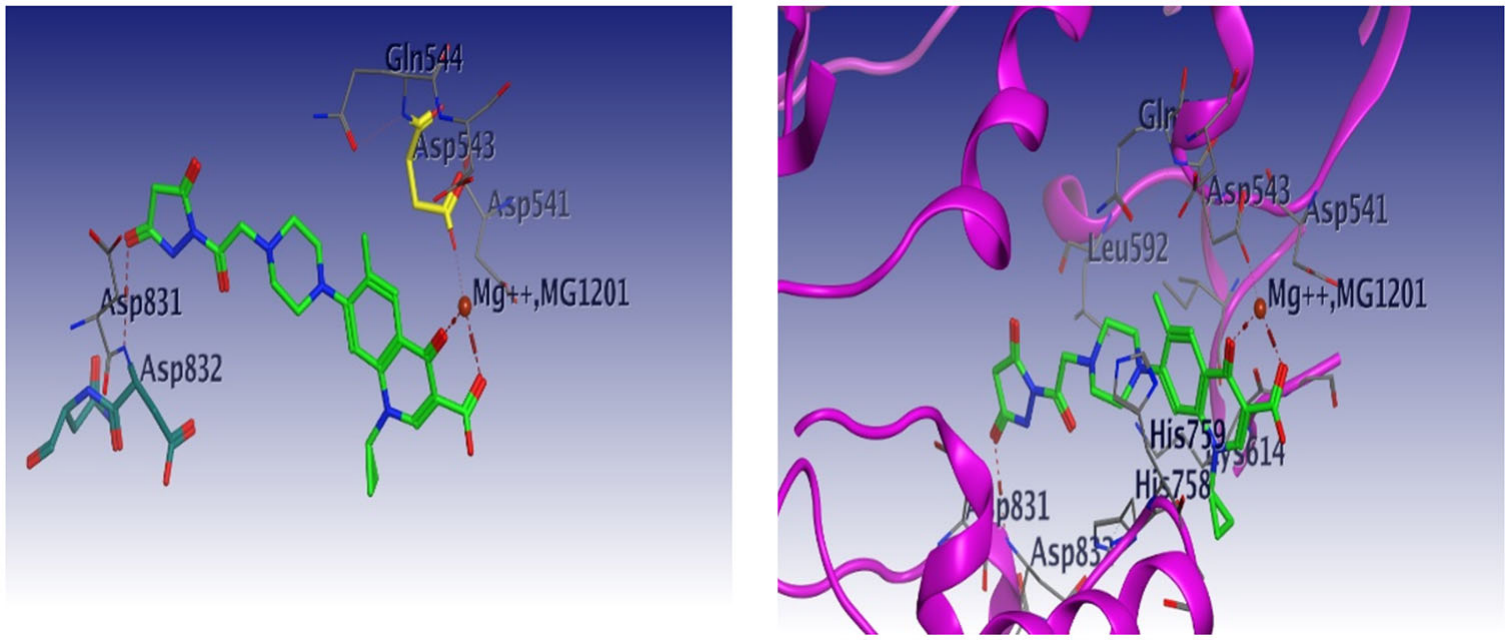
3D interaction of compound **6** with DNA binding site of topoisomerase IIα. Red dashed lines represent coordinate bond interactions with Mg^2+^. Red tiny, dashed lines are hydrogen bonding interactions with amino acid Asp 543, and Asp 831. Mg^2+^ is shown as a nonbonded sphere (crimson red). Residues that are involved in hydrogen bonding are shown in the stick presentation.

**Figure 20.**
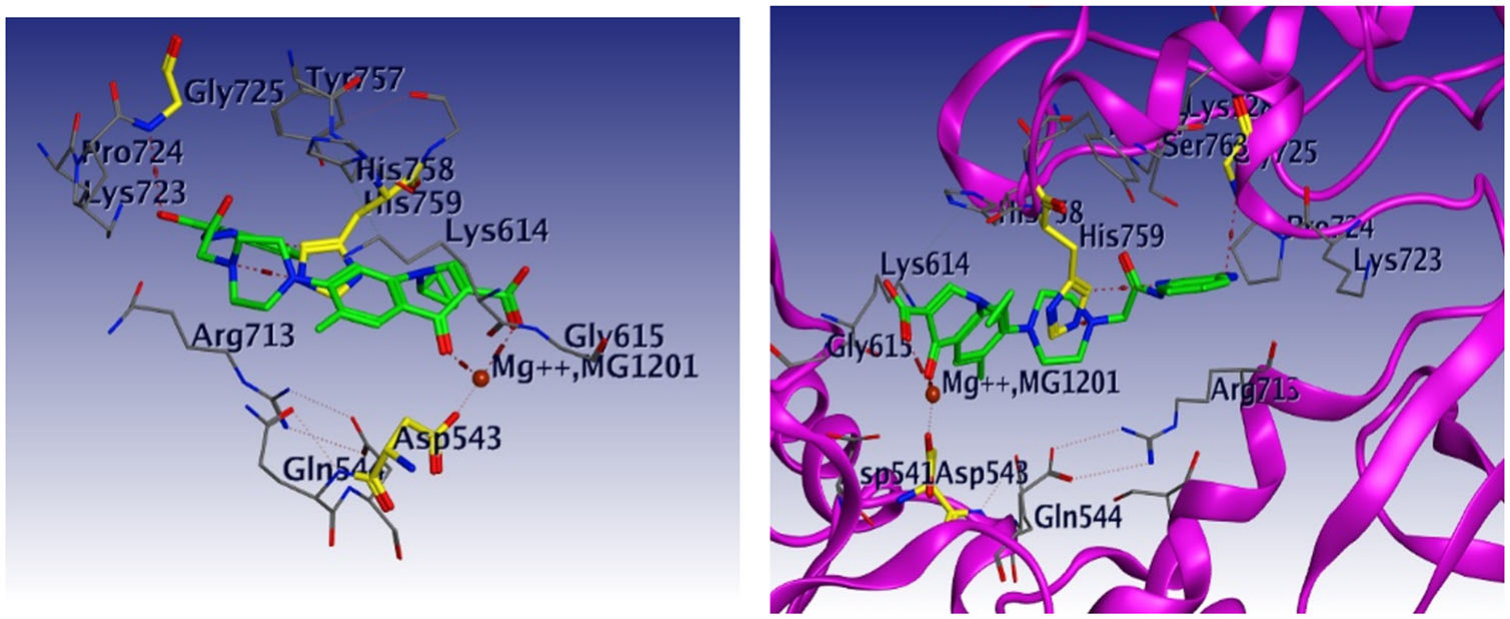
3D interaction of compound **8a** with DNA binding site of topoisomerase IIα. Red dashed lines represent coordinate bond interactions with Mg^2+^. Red tiny, dashed lines are hydrogen bonding interactions with amino acid Asp 543, acids His 759 and Gly 725. Mg^2+^ is shown as a nonbonded sphere (crimson red). Residues that are involved in hydrogen bonding are shown in the stick presentation.

Docking studies were carried out for compounds **6** and **8a** which showed potent activity in the Topo II enzyme inhibition assay. We used topoisomerase IIα co-crystallised with DNA (PDB ID: 4FM9)^63^ for molecular docking of compounds **6**, **8a**, and merbarone. Docking of merbarone in the DNA binding site was carried out for validation of the molecular docking and it was compared with a previously reported study^78^. Interestingly, the test compounds exerted favourable interactions with the Topo II enzyme active site, with almost the same binding pattern as merbarone. Merbarone showed coordinate bond interactions with Mg^2+^ and H-bond interaction with amino acid Asp 543 (Figure 18). Compound **6** interacted in a similar pattern, the oxygen atoms of both coplanar carbonyl groups at positions 3 and 4 formed coordinate bond interactions with Mg^2+^ and H-bond interaction with amino acid Asp 543. Moreover, compound **6** interacted as an H-bond acceptor through the carbonyl group of its pyrazolidine moiety with amino acid Asp 831(Figure 19). Regarding compound **8a**, it interacted with the Topo II enzyme through coordinate bonding with the magnesium ion Mg^2+^ via the oxygen atoms of both carbonyl groups at positions 3 and 4 and H-bond interaction with amino acid Asp 543. Additionally, it interacted as an H-bond acceptor with amino acids His 759 and Gly 725 through the N atom of piperazine and carbonyl group of indoline moiety, respectively. It exerted arene cation interaction with Arg 713 (Figure 20).

## Conclusion

In summary, a series of novel CP derivatives were designed and synthesised. They were evaluated for their anti-proliferative activity against bladder T-24 and prostate PC-3 cancer cell lines. 14 compounds exhibited potent antiproliferative activity against the T-24 cell line, with IC_50_ values between 3.36 and 28.55 µM, which was 1.02- to 8.66-fold more potent than the reference drug doxorubicin. 10 compounds proved their potency against the PC-3 cell line with IC_50_ values between 3.24 and 19.33 µM, which was 1.2- to 7.1-fold more potent than doxorubicin. The most potent compounds **6**, **7a**, **7b**, **8a**, **9a**, and **10c** showed significant Topo II inhibitory activity. Compounds **6**, **8a**, and **10c** were 1.01- to 2.32-fold more potent than doxorubicin. The most prominent compounds **6** and **8a** were further investigated regarding their effects on cell cycle progression, induction of apoptosis, and level of active caspase-3 in the T-24 and PC-3 cell lines. Both compounds induced apoptosis in T-24 cells (16.8- and 20.1-fold, respectively compared to control). This evidence was supported by an increase in the level of apoptotic caspase-3 (5.23- and 7.6-fold). Compounds **6** and **8a** arrested the cell cycle in the S phase. Regarding the induction of apoptosis in PC-3 cell lines, both compounds **6** and **8a** induced G1-phase cell cycle arrest at their IC_50_ concentrations. This was confirmed by the increase in the percentage of cells in the G1 phase (1.16- and 1.27-fold, respectively), and a concomitant decrease in the percentage of cells in G2/M phase, (1.5- and 20.1-fold, respectively). The treatment of PC-3 cells with compounds **6** and **8a** at their IC_50_ concentrations resulted in a decrease in the percentage of viable cells. The results showed that both compounds **6** and **8a** induced both early and late apoptosis. The molecular docking study of these compounds with Topo II protein revealed more favourable binding modes compared to merbarone, explaining their remarkable Topo II inhibitory potency. CP derivatives are promising leads for further studying, designing, and synthesis of potent anti-proliferative candidates.

## Supplementary Material

Supplemental Material
