# Supplementary material for: Design, synthesis, and biological evaluation of novel ciprofloxacin derivatives as potential anticancer agents targeting topoisomerase II enzyme
Source: J Enzyme Inhib Med Chem. 2022 Oct 28;38(1):118–37. doi: 10.1080/14756366.2022.2136172 (PMC9635472; doi:10.1080/14756366.2022.2136172)
Supplement: Supplemental Material [file IENZ_A_2136172_SM7502.pdf]

Supporting information

Design, Synthesis, and Biological Evaluation of Novel Ciprofloxacin Derivatives as Potential Anticancer Agents Targeting Topoisomerase II enzyme.

Hadeer K. Swedan[1], Asmaa E. Kassab [2,] Ehab M. Gedawy[2,3,] Salwa E. Elmeligie[2]

[1] Central Administration of Research and Health Development, Ministry of Health, and Population (MoHP), Cairo, P.O. Box 11516, Egypt.

[2] Department of Pharmaceutical Organic Chemistry, Faculty of Pharmacy, Cairo University, Kasr El-Aini Street, Cairo, P.O. Box 11562, Egypt.

[3] Department of Pharmaceutical Chemistry, Faculty of Pharmacy and Pharmaceutical Industries, Badr University in Cairo (BUC), Badr City, Cairo, P.O. Box 11829, Egypt.

Correspondence to: Asmaa E. Kassab, Department of Pharmaceutical Organic Chemistry, Faculty of Pharmacy, Cairo University, Cairo, P.O. Box 11562, Egypt.

Tel: 002023639307.

Fax: 002023635140.

E-mail: asmaa.kassab@pharma.cu.edu.eg

Address: 33 Kasr El-Aini Street, Cairo, Egypt.

Information Classification: General

Compound 3

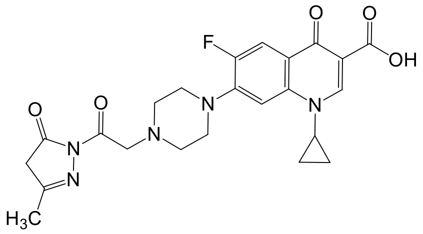

O
O
F
OH
N
N
O
O
N
N
N
H3C

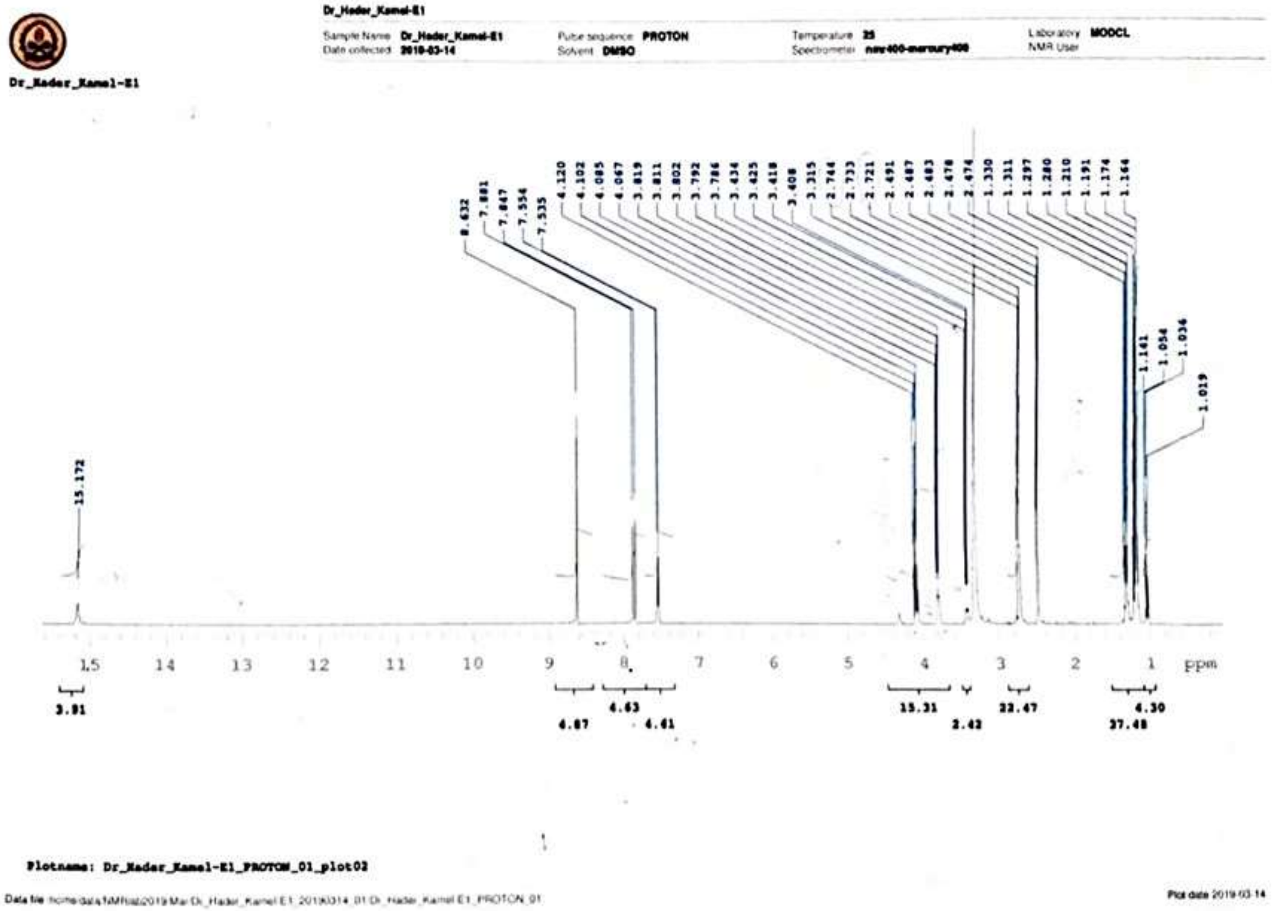

Dr_Hader_Kamel-E1
Sample Name Dr_Hader_Kamel-E1
Date collected 2019-03-14
Pulse sequence PROTON
Solvent DMSO
Temperature 25
Laboratory MODCL
NMR User
Dr_Hader_Kamel-E1
ppm
Plotname: Dr_Hader_Kamel-E1_PROTON_01_plot02
Plot date 2019-03-14

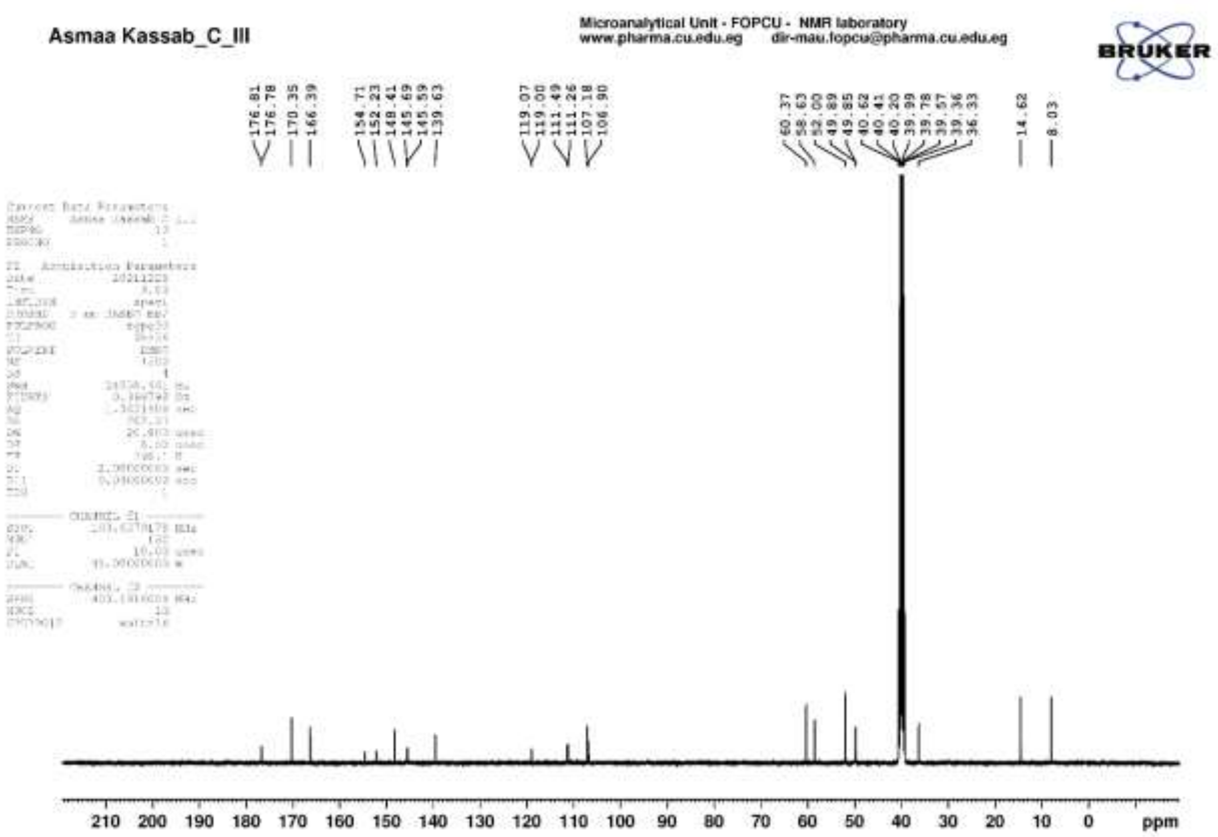

Information Classification: General

Compound 4

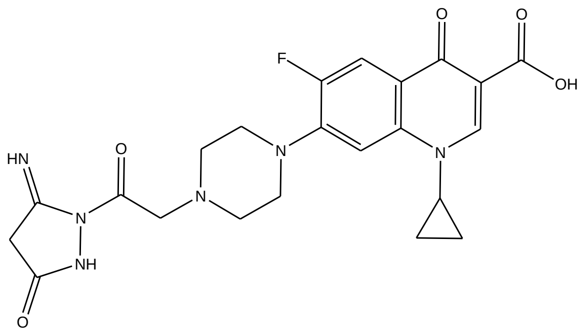
F
O
O
OH
N
N
N
HN
O
N
NH
O

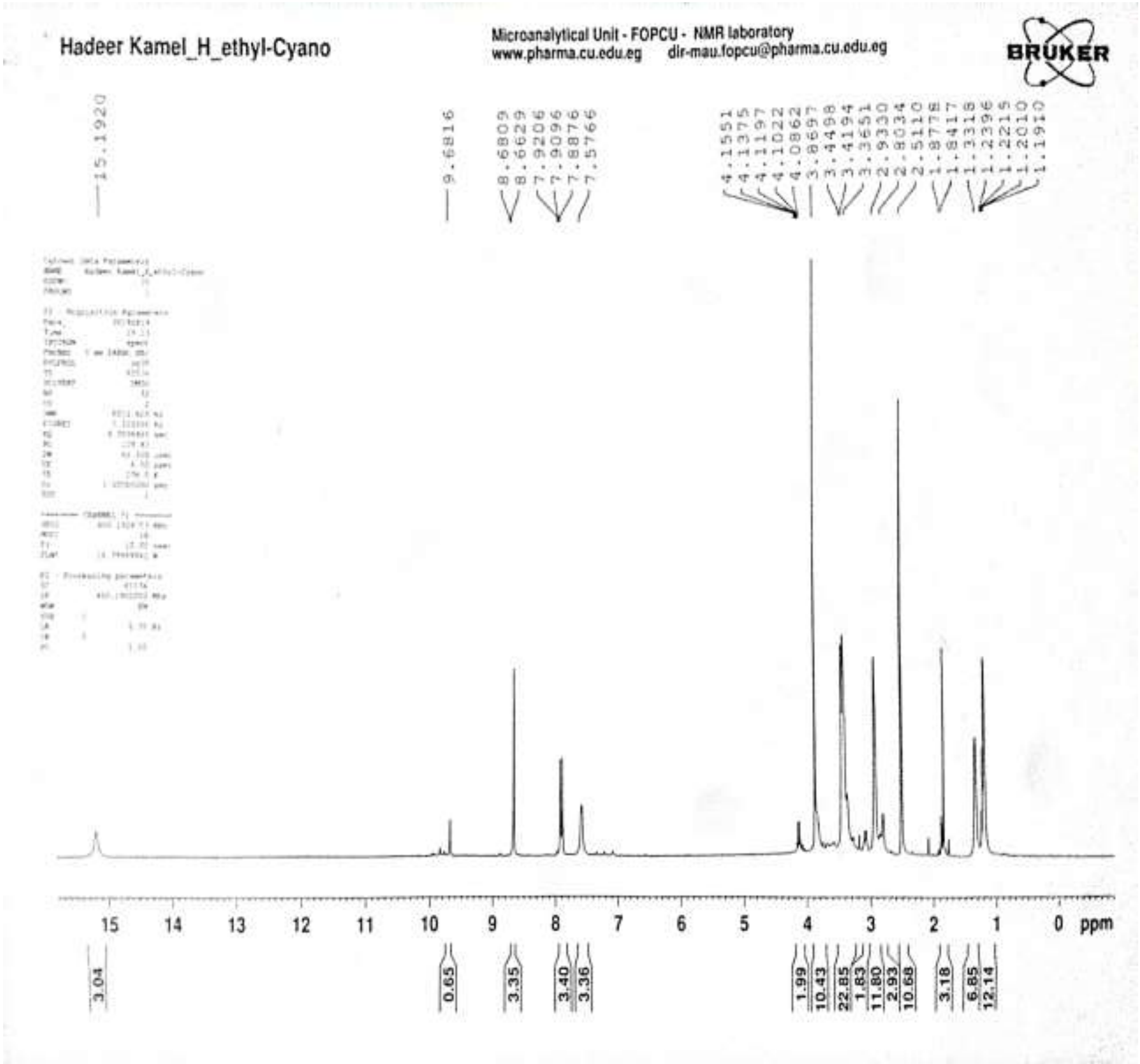
Hadeer Kamel_H_ethyl-Cyano
Microanalytical Unit - FOPCU - NMR laboratory
www.pharma.cu.edu.eg dir-mau.fopcu@pharma.cu.edu.eg
15.1920
9.6816
8.6809
8.6629
7.9206
7.9096
7.8876
7.5766
4.1551
4.1375
4.1197
4.1022
4.0862
3.8697
3.4498
3.4194
3.3651
2.9330
2.8034
2.5110
1.8778
1.8417
1.3318
1.2396
1.2215
1.2010
1.1910
15 14 13 12 11 10 9 8 7 6 5 4 3 2 1 0 ppm
3.04
0.65
3.35
3.40
3.36
1.99
10.43
22.85
1.83
11.80
2.93
10.68
3.18
6.85
12.14

Asmaa Kassab_C_IV

Microanalytical Unit - FOPCU - NMR laboratory
www.pharma.cu.edu.eg dir-mau.fopcu@pharma.cu.edu.eg

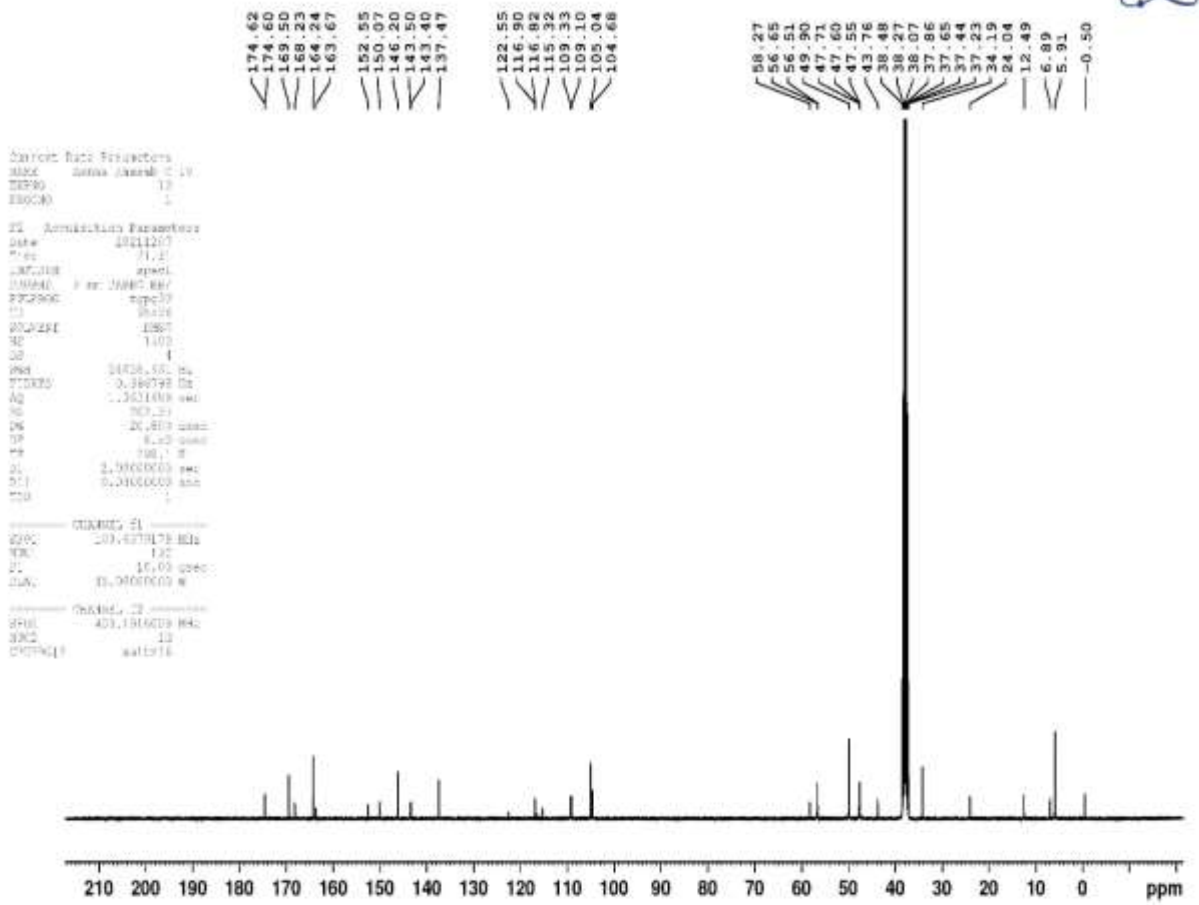

Information Classification: General

Compound 5

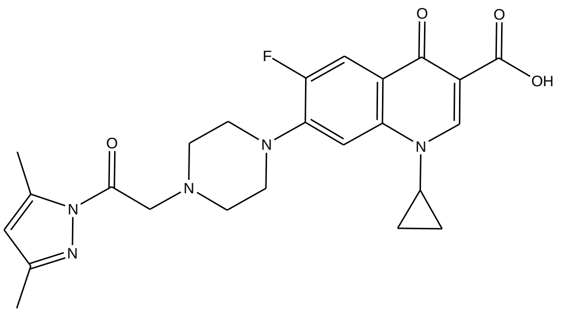

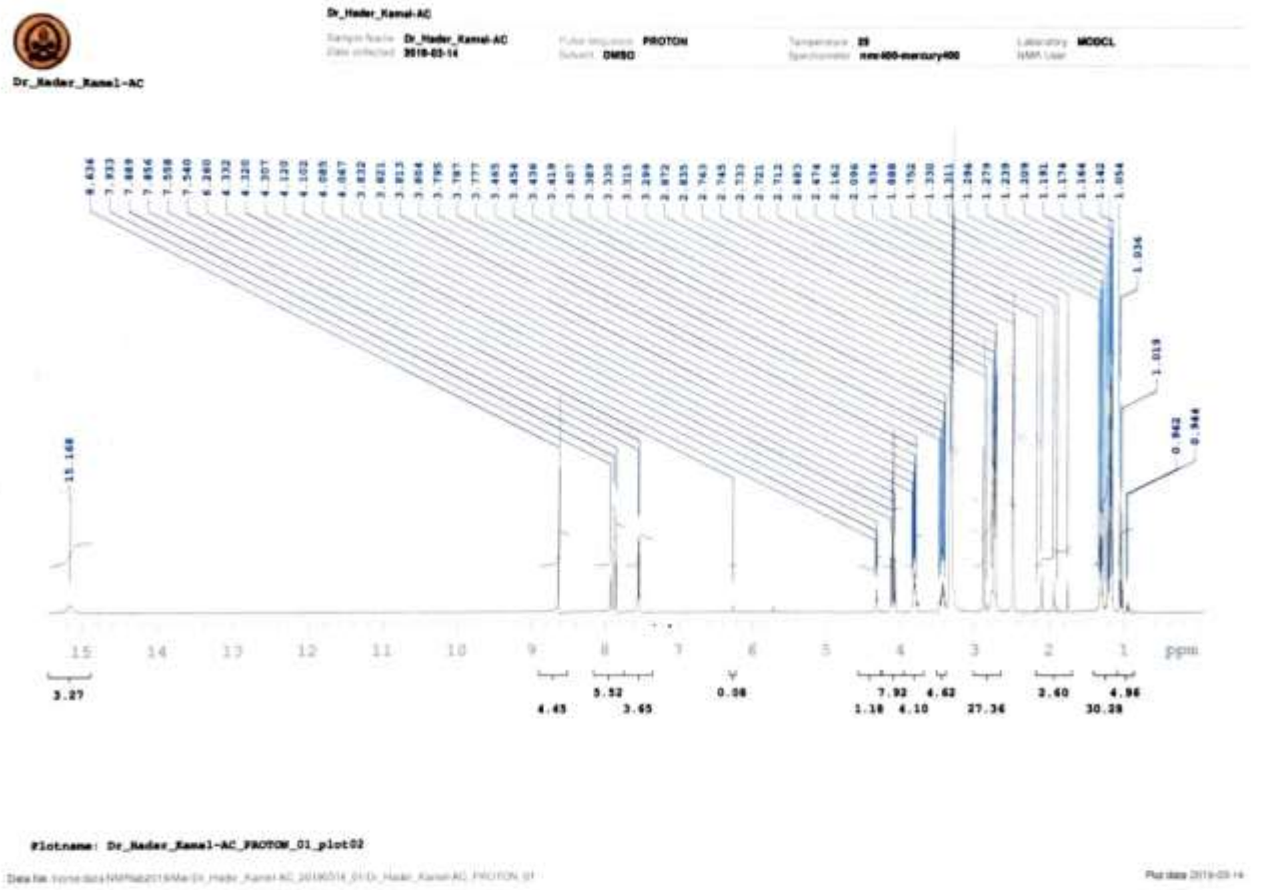

Information Classification: General

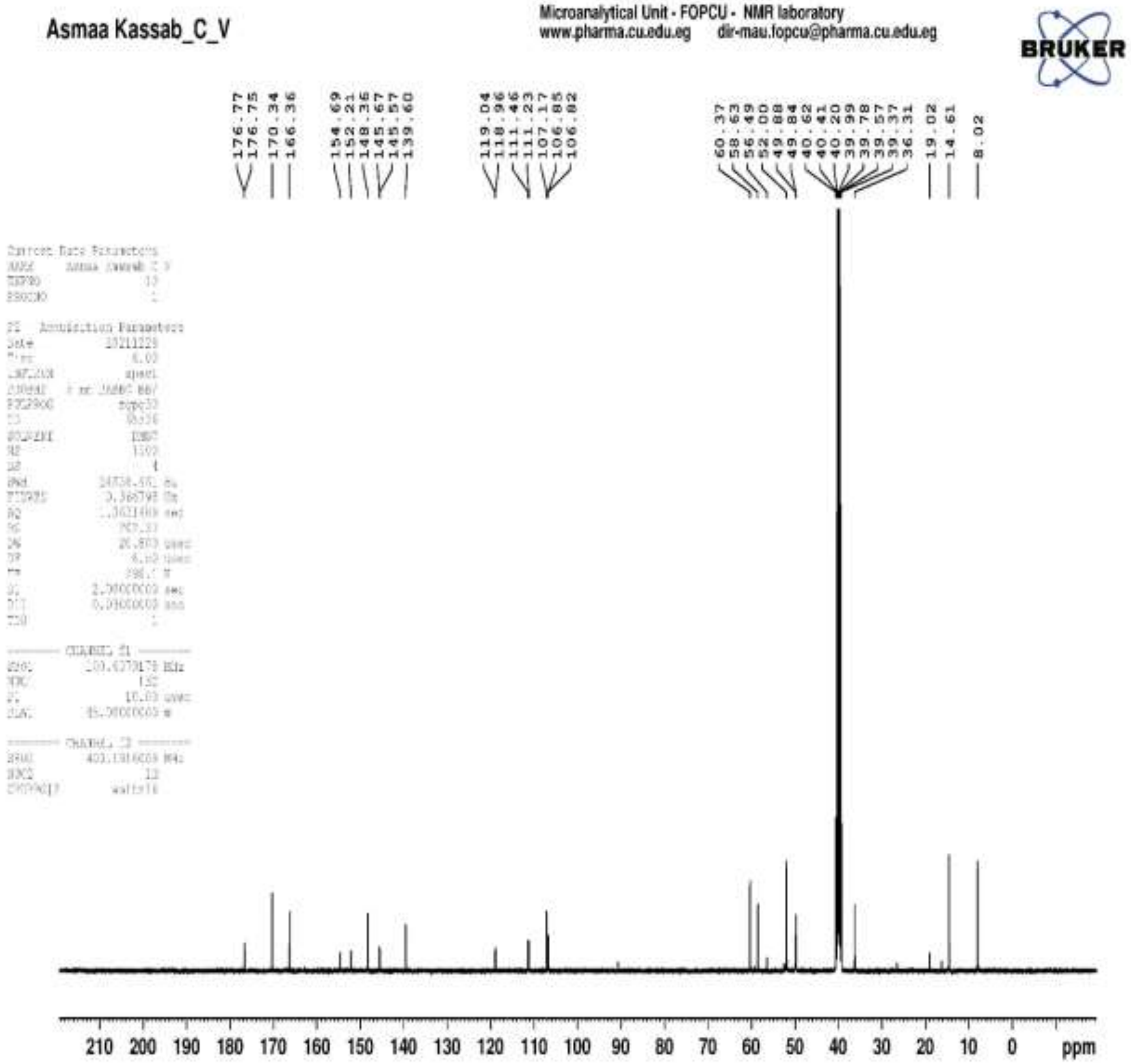

Information Classification: General

Compound 6

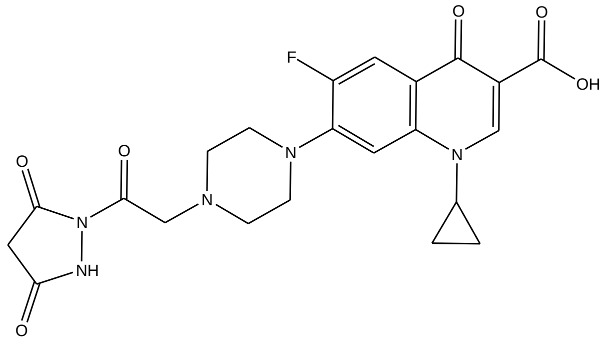

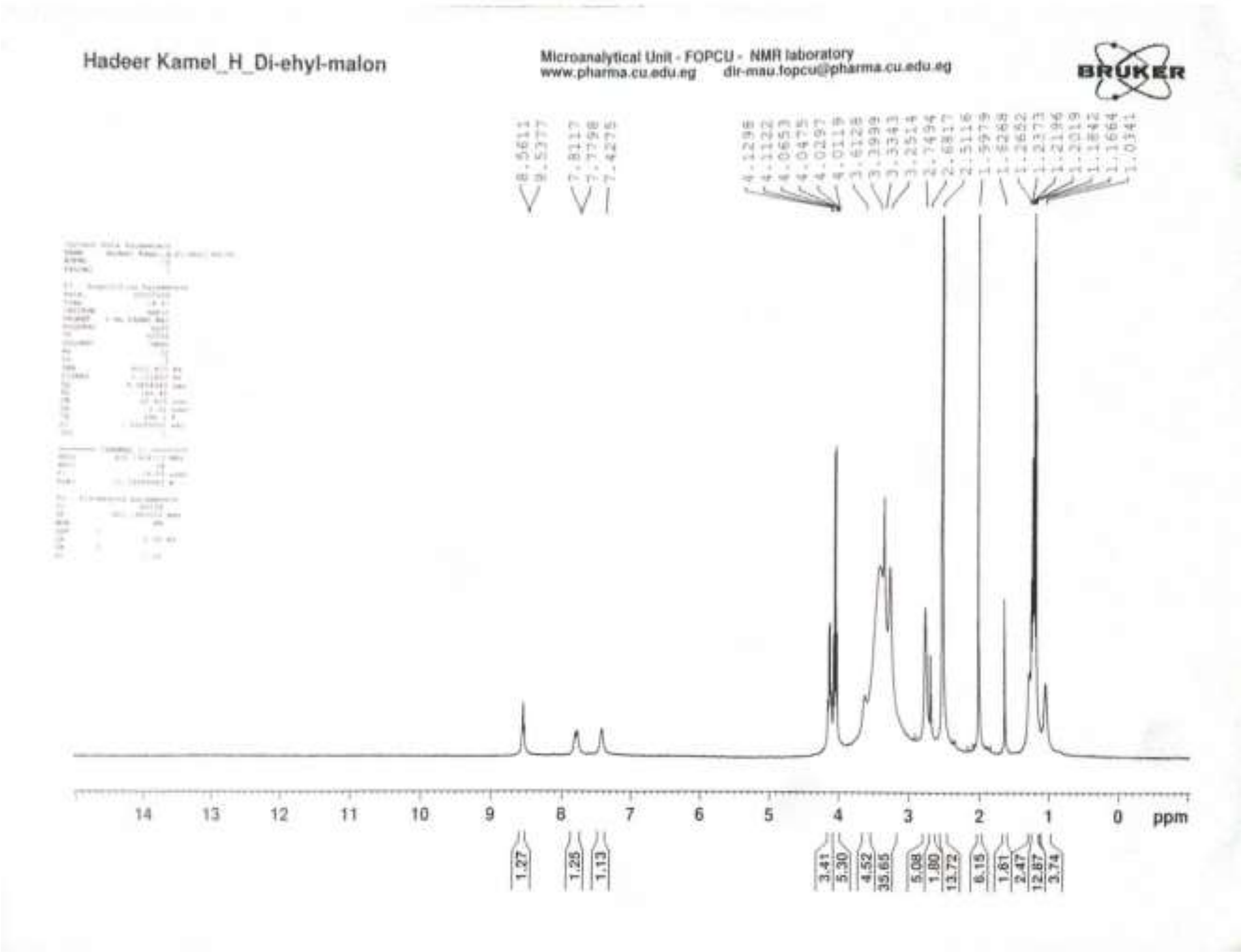
Hadeer Kamel_H_Di-ehyl-malon
Microanalytical Unit - FOPCU - NMR laboratory
www.pharma.cu.edu.eg dir-mau.fopcu@pharma.cu.edu.eg
ppm

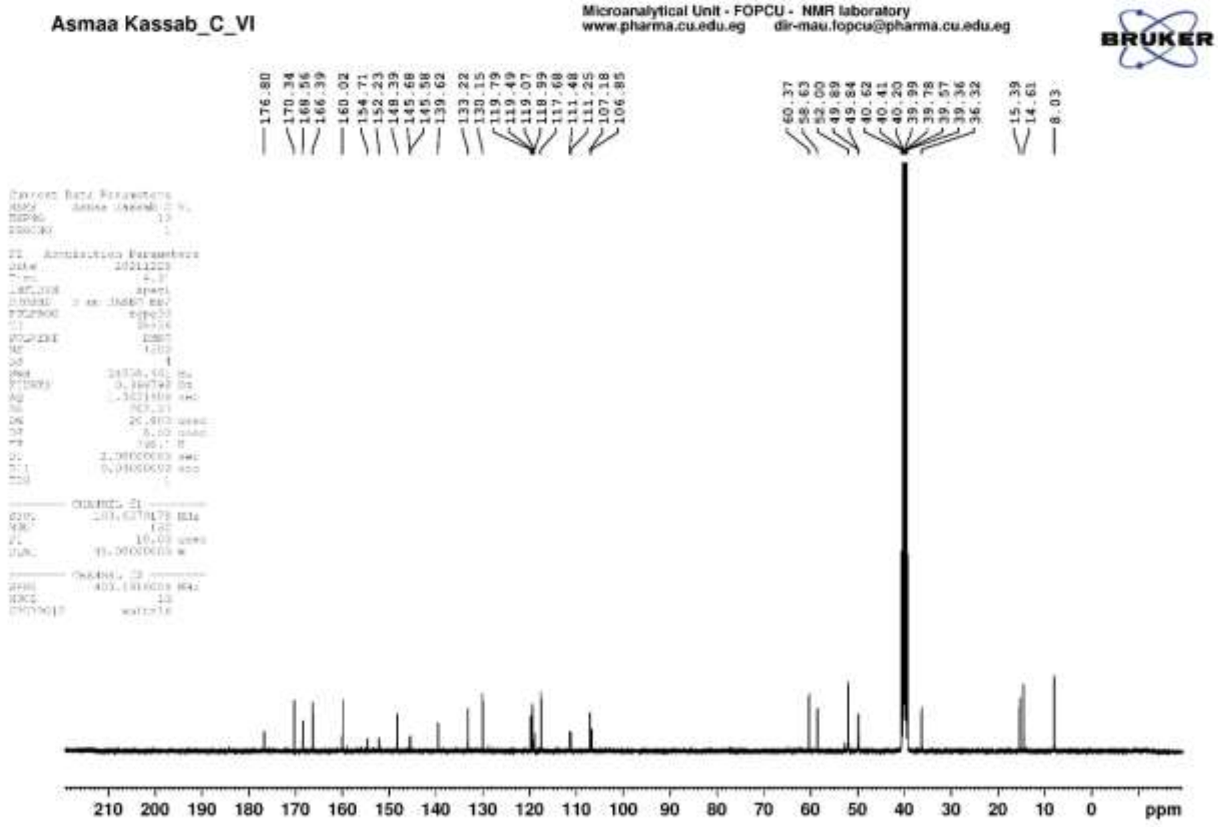

Asmaa Kassab_C_VI
Microanalytical Unit - FOPCU - NMR laboratory
www.pharma.cu.edu.eg dir-mau.fopcu@pharma.cu.edu.eg
176.80
170.34
168.56
166.39
160.02
154.71
152.23
148.39
145.68
145.58
139.62
133.22
130.15
119.79
119.49
119.07
118.99
117.68
111.48
111.25
107.18
106.85
60.37
58.63
52.00
49.89
49.84
40.62
40.41
40.20
39.99
39.78
39.57
39.36
36.32
15.39
14.61
8.03
210 200 190 180 170 160 150 140 130 120 110 100 90 80 70 60 50 40 30 20 10 0 ppm

Compound 7a

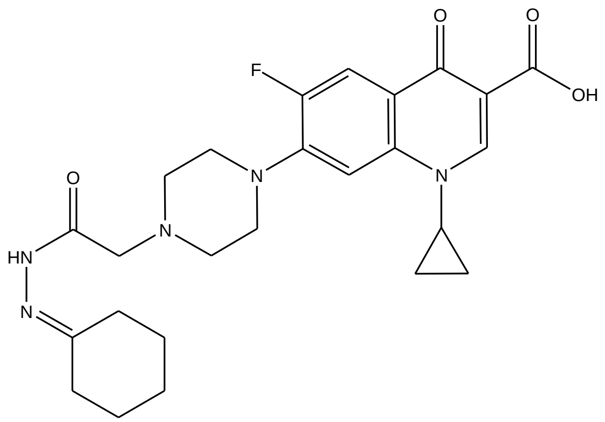

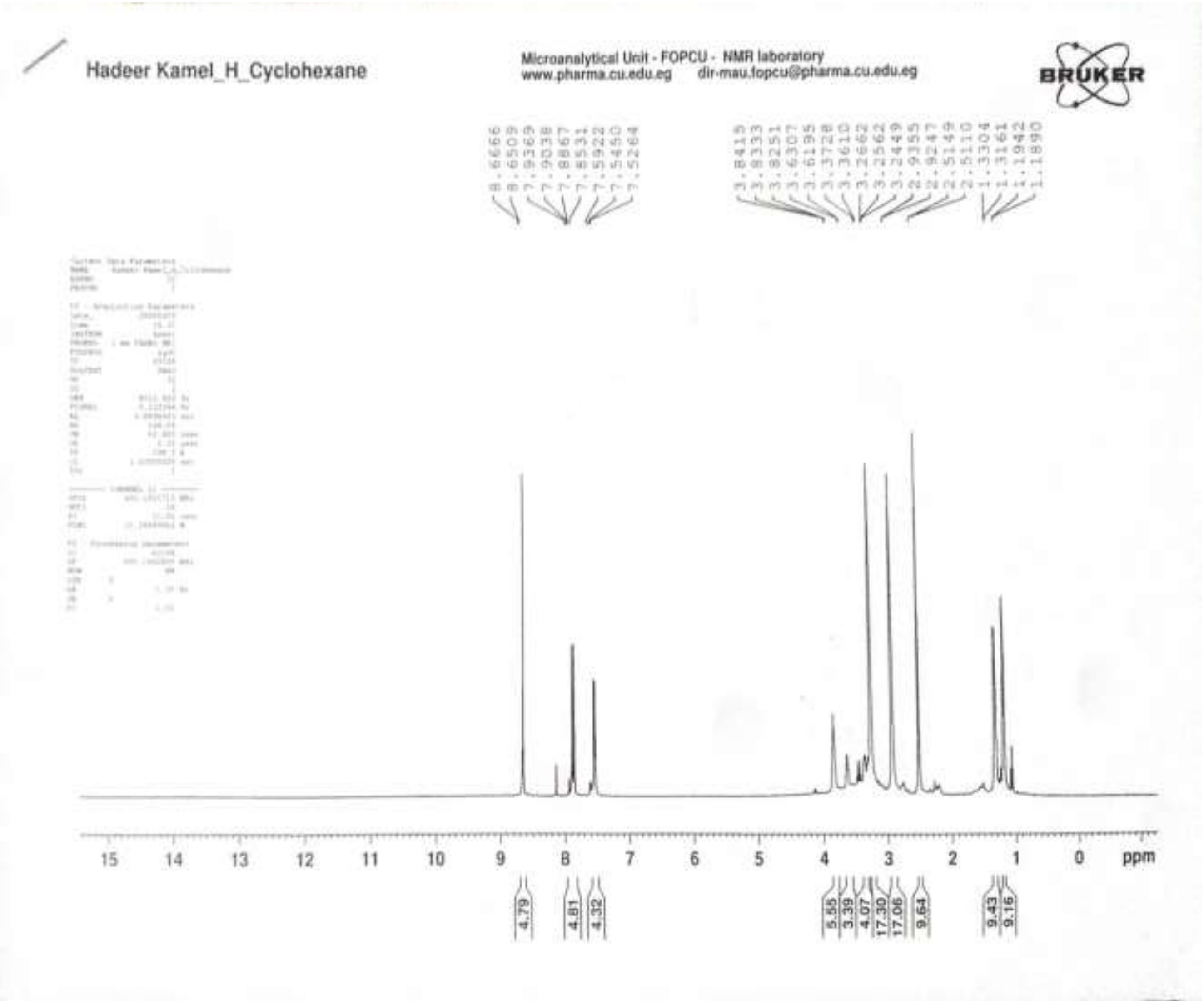

Hadeer Kamel_H_Cyclohexane
Microanalytical Unit - FOPCU - NMR laboratory
www.pharma.cu.edu.eg dir-mau.fopcu@pharma.cu.edu.eg
BRUKER
8.6666
8.6509
7.9369
7.9038
7.8867
7.8531
7.5922
7.5450
7.5264
3.8415
3.8333
3.8251
3.6307
3.6195
3.3728
3.3610
3.2662
3.2562
3.2449
2.9355
2.9247
2.5149
2.5110
1.3304
1.3161
1.1942
1.1890
15
14
13
12
11
10
9
8
7
6
5
4
3
2
1
0
ppm
4.79
4.81
4.32
5.55
3.39
4.07
17.30
17.06
9.64
9.43
9.16

Asmaa Kassab_C_VII

Microanalytical Unit - FOPCU - NMR laboratory
www.pharma.cu.edu.eg dir-mau.fopcu@pharma.cu.edu.eg

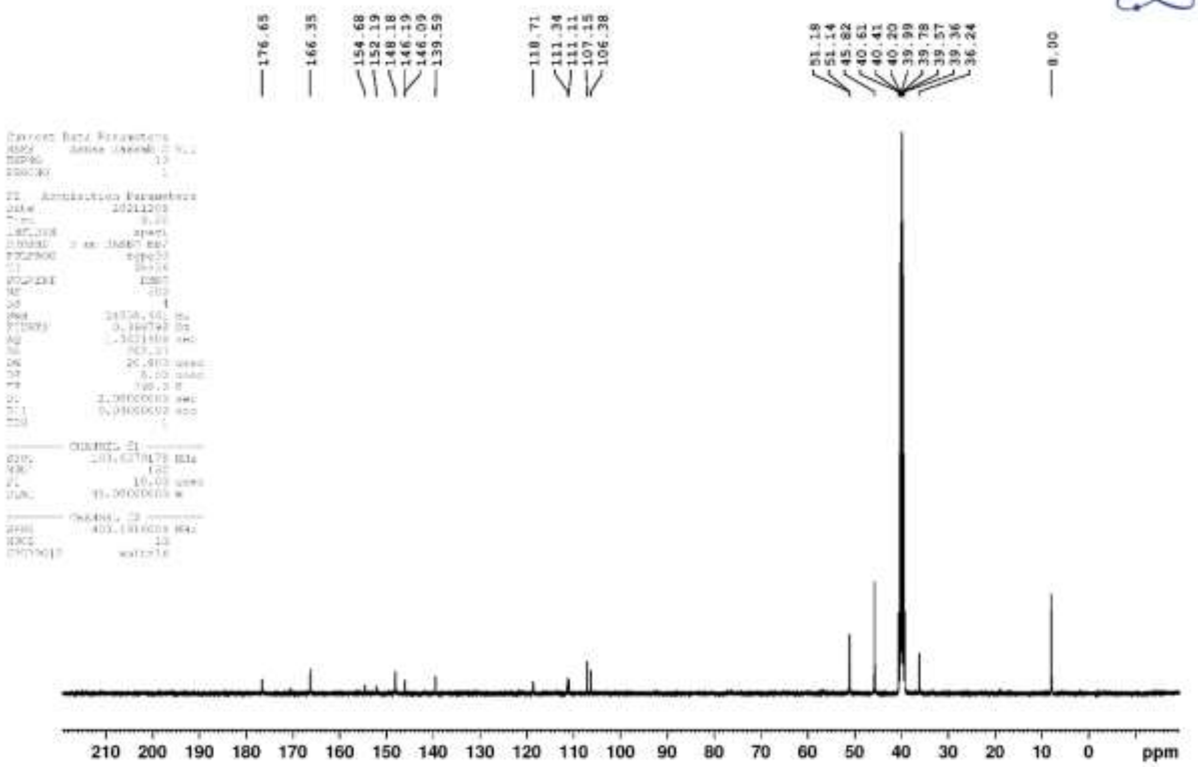

Information Classification: General

Compound 7b

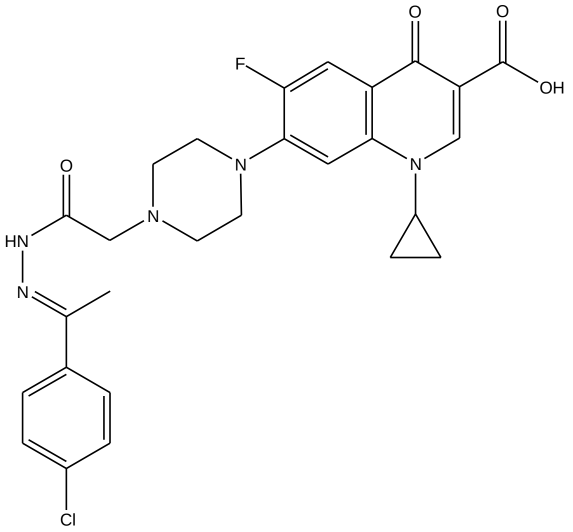

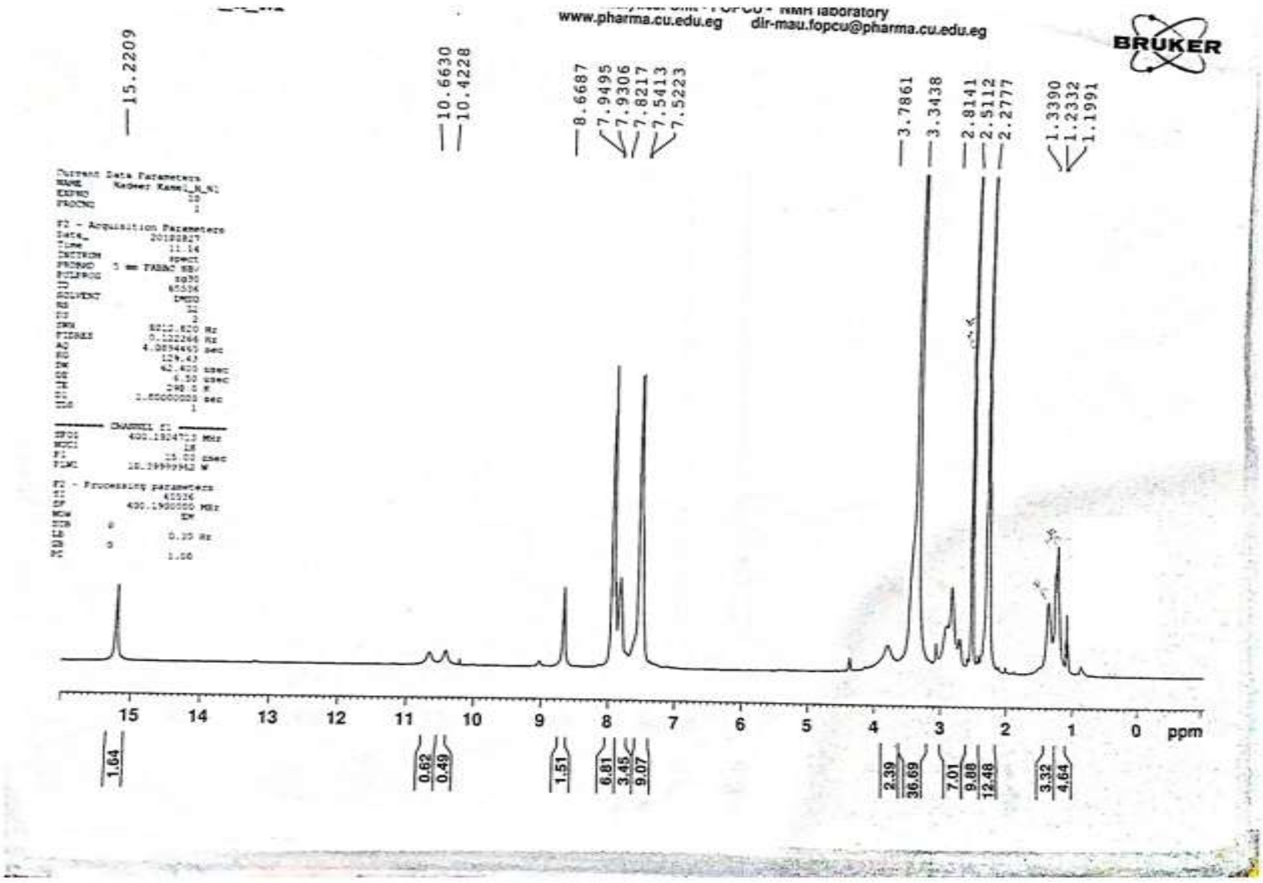

Information Classification: General

Compound 7d

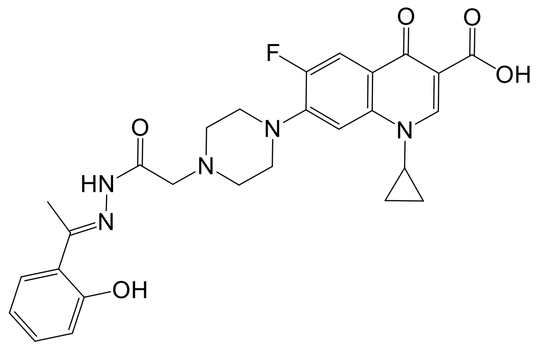

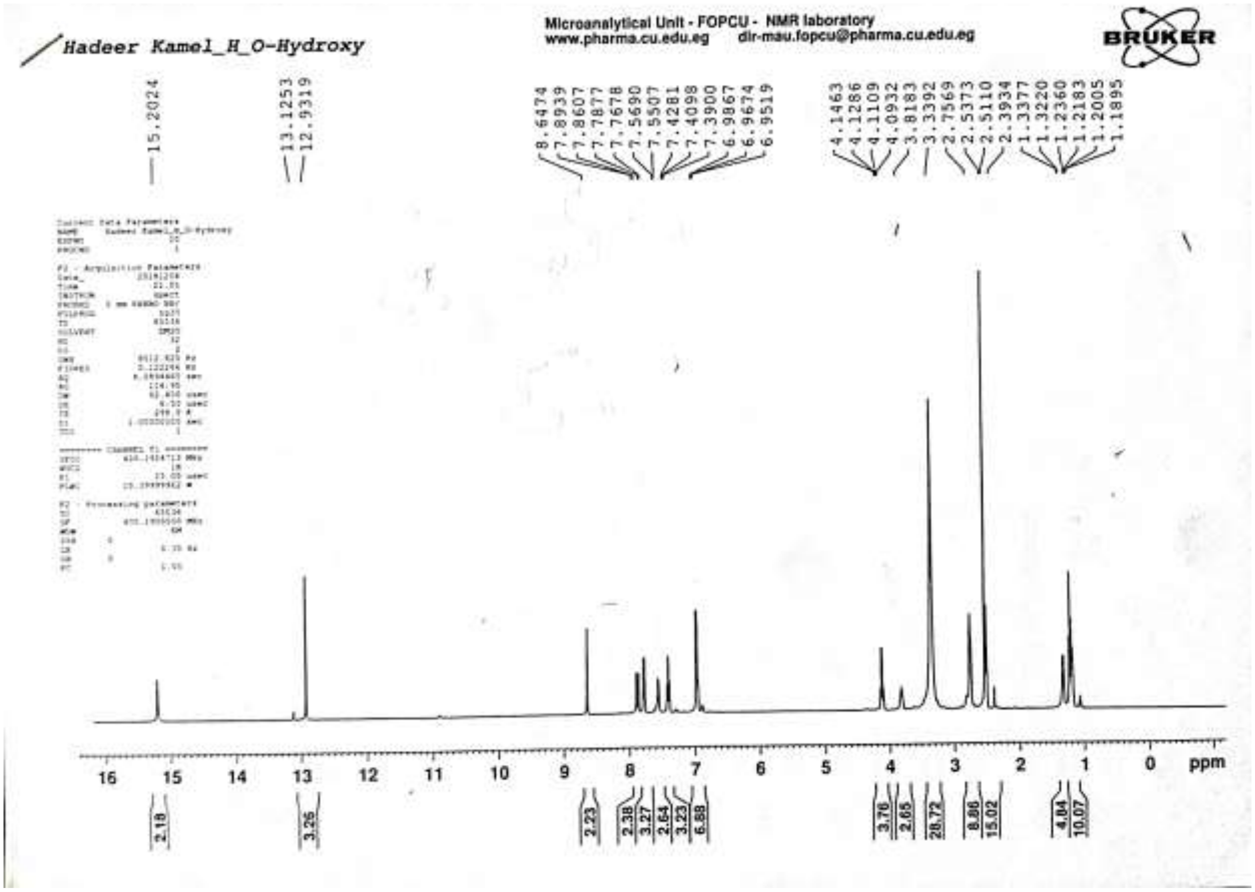

Hadeer Kamel_H_O-Hydroxy
Microanalytical Unit - FOPCU - NMR laboratory
www.pharma.cu.edu.eg dir-mau.fopcu@pharma.cu.edu.eg
BRUKER
15.2024
13.1253
12.9319
8.6474
7.8939
7.8607
7.7877
7.7678
7.5690
7.5507
7.4281
7.4098
7.3900
6.9867
6.9674
6.9519
4.1463
4.1286
4.1109
4.0932
3.8183
3.3392
2.7569
2.5373
2.5110
2.3934
1.3377
1.3220
1.2360
1.2183
1.2005
1.1895
ppm
2.18
3.26
2.23
2.38
3.27
2.64
3.23
6.88
3.76
2.65
28.72
8.86
15.02
4.84
10.07

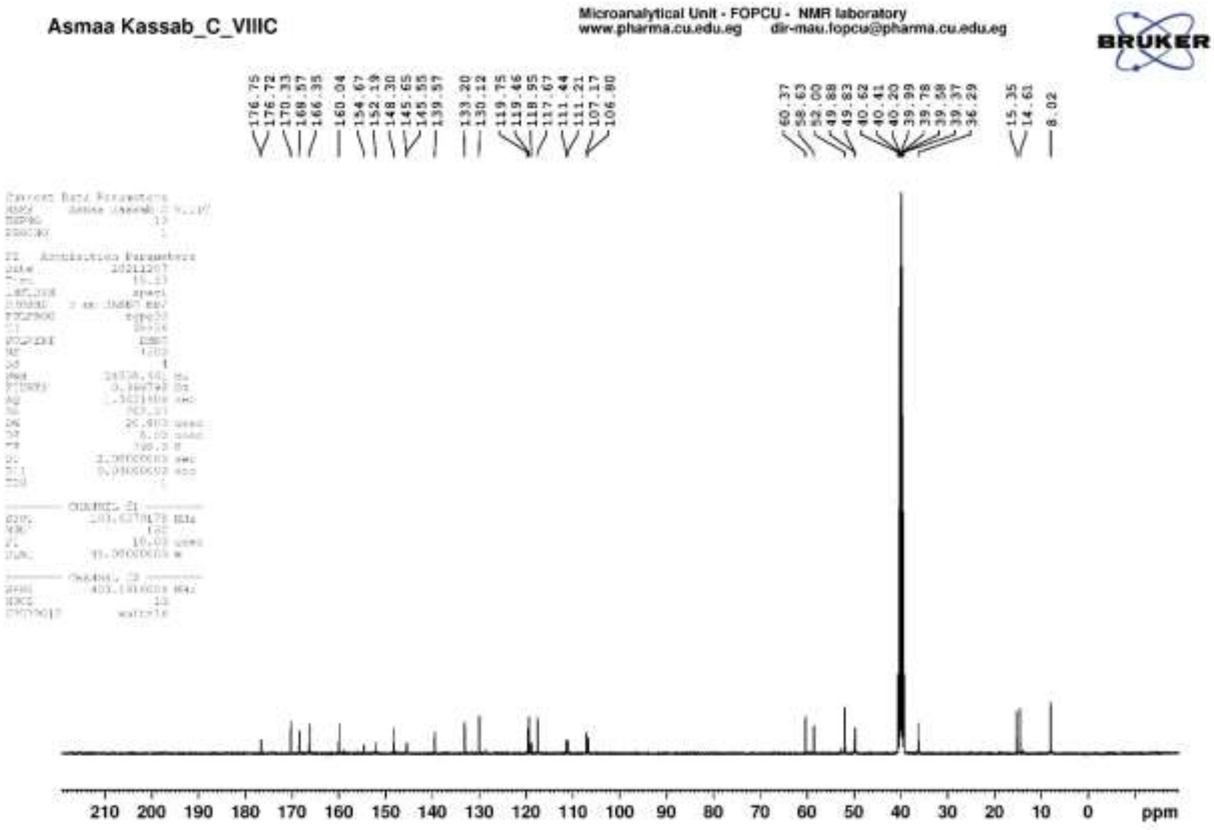

Information Classification: General

Compound 7e

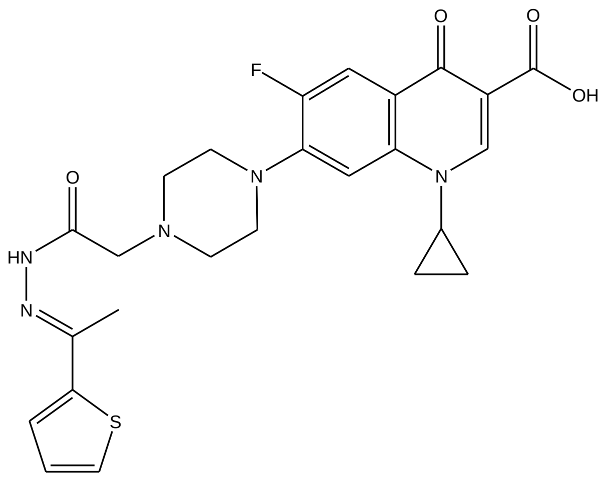

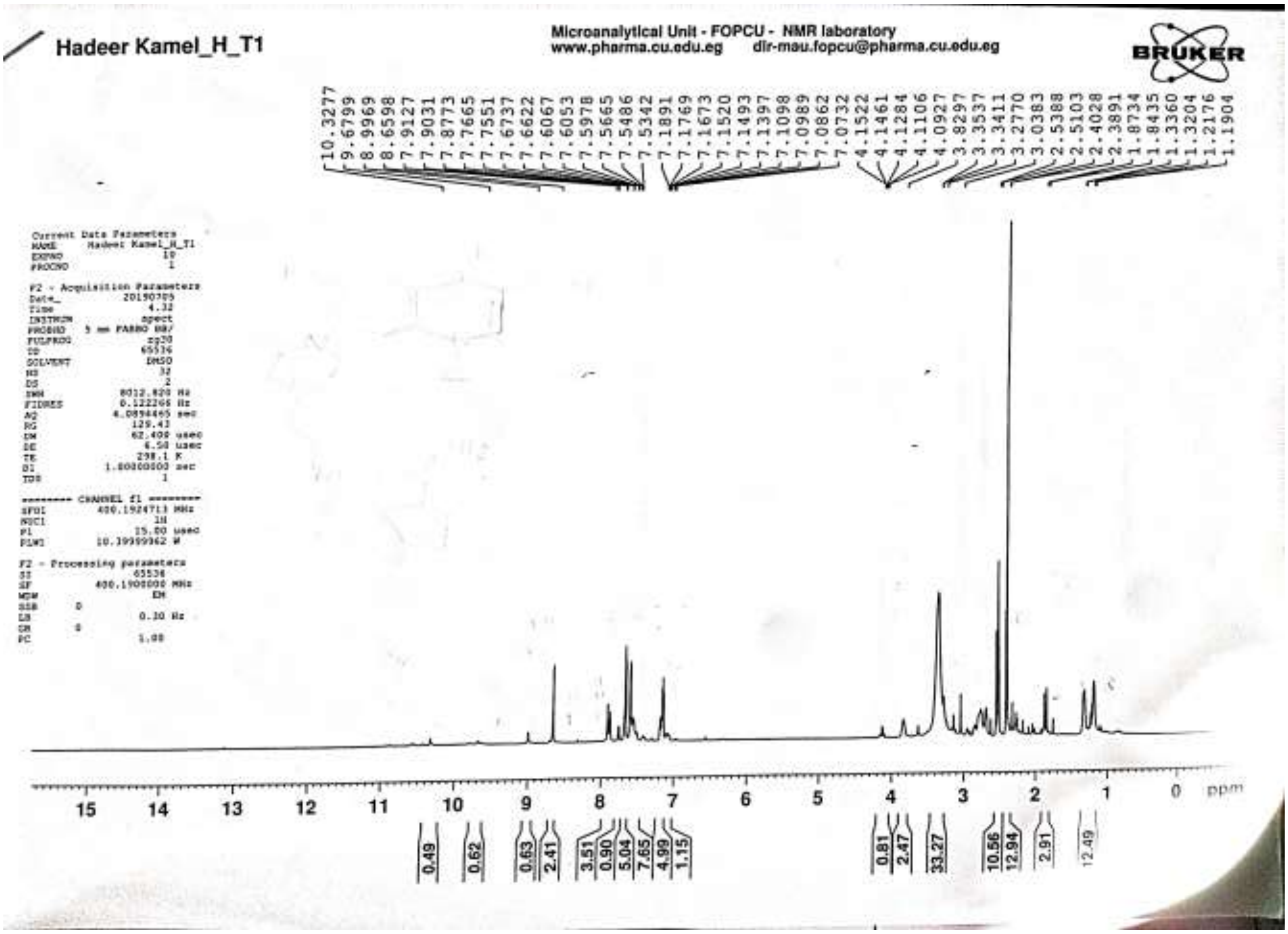

Asmaa Kassab_C_VIIId

Microanalytical Unit - FOPCU - NMR laboratory
www.pharma.cu.edu.eg dir-mau.fopcu@pharma.cu.edu.eg

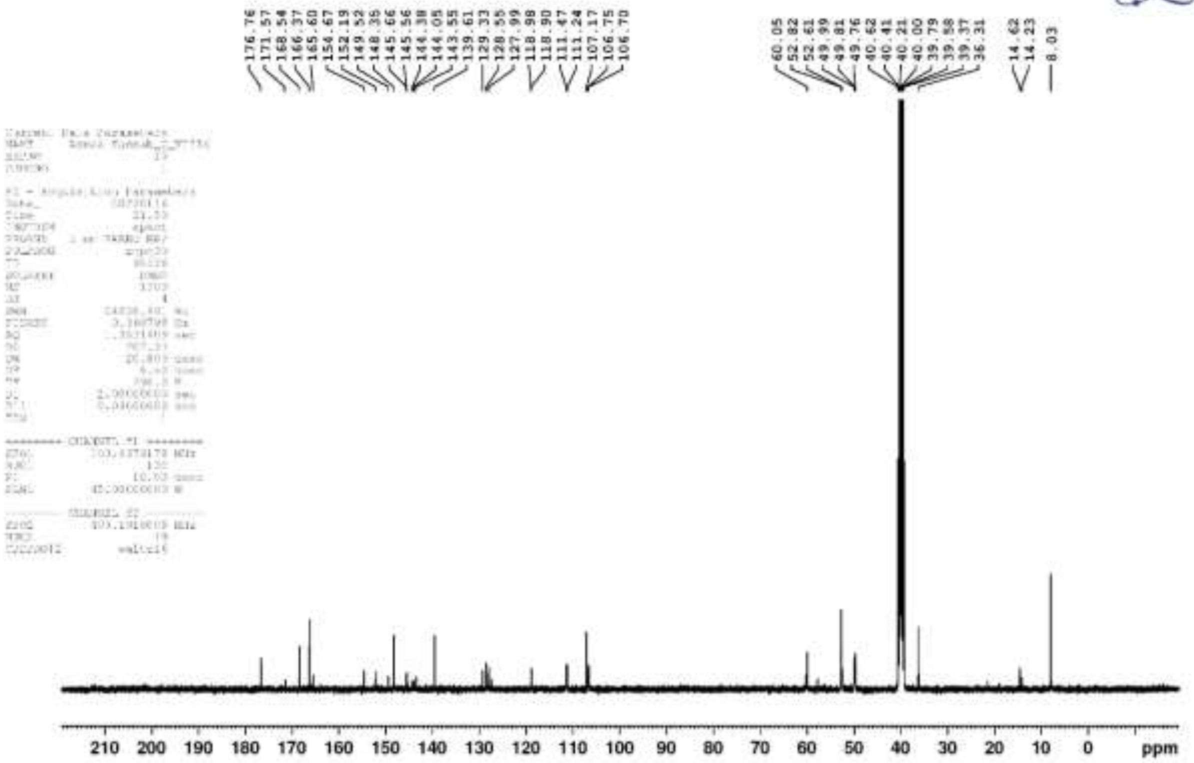

Information Classification: General

Compound 8a

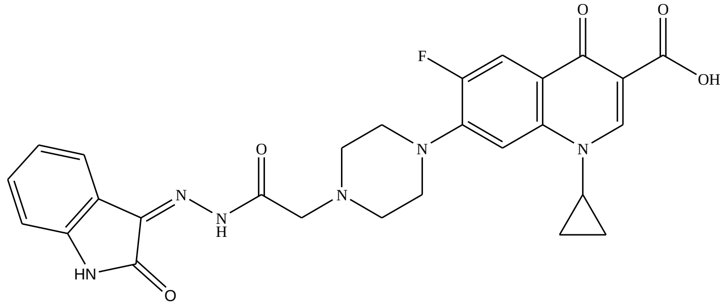
O
O
F
OH
N
N
O
N
N
N
H
HN
O

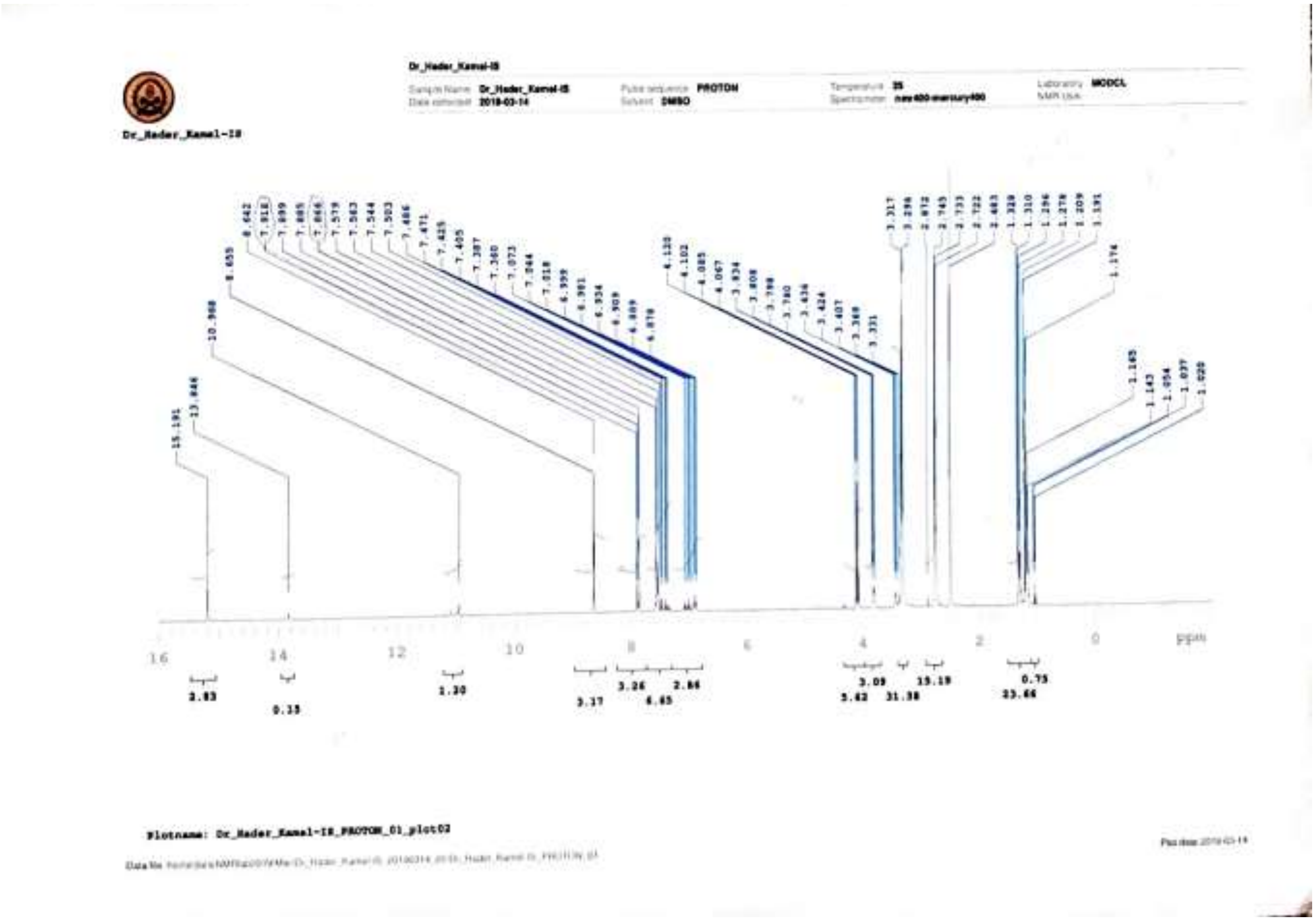
Dr_Hader_Kamel-IS
Sample Name Dr_Hader_Kamel-IS
Date collected 2018-03-14
Pulse sequence PROTON
Solvent DMSO
Temperature 25
Spectrometer nmr400-mercury400
Laboratory MODCL
Dr_Hader_Kamel-I8
ppm
Plotname: Dr_Hader_Kamel-I8_PROTON_01_plot02

Asmaa Kassab_C_IXa

Microanalytical Unit - FOPCU - NMR laboratory
www.pharma.cu.edu.eg dir-mau.fopcu@pharma.cu.edu.eg

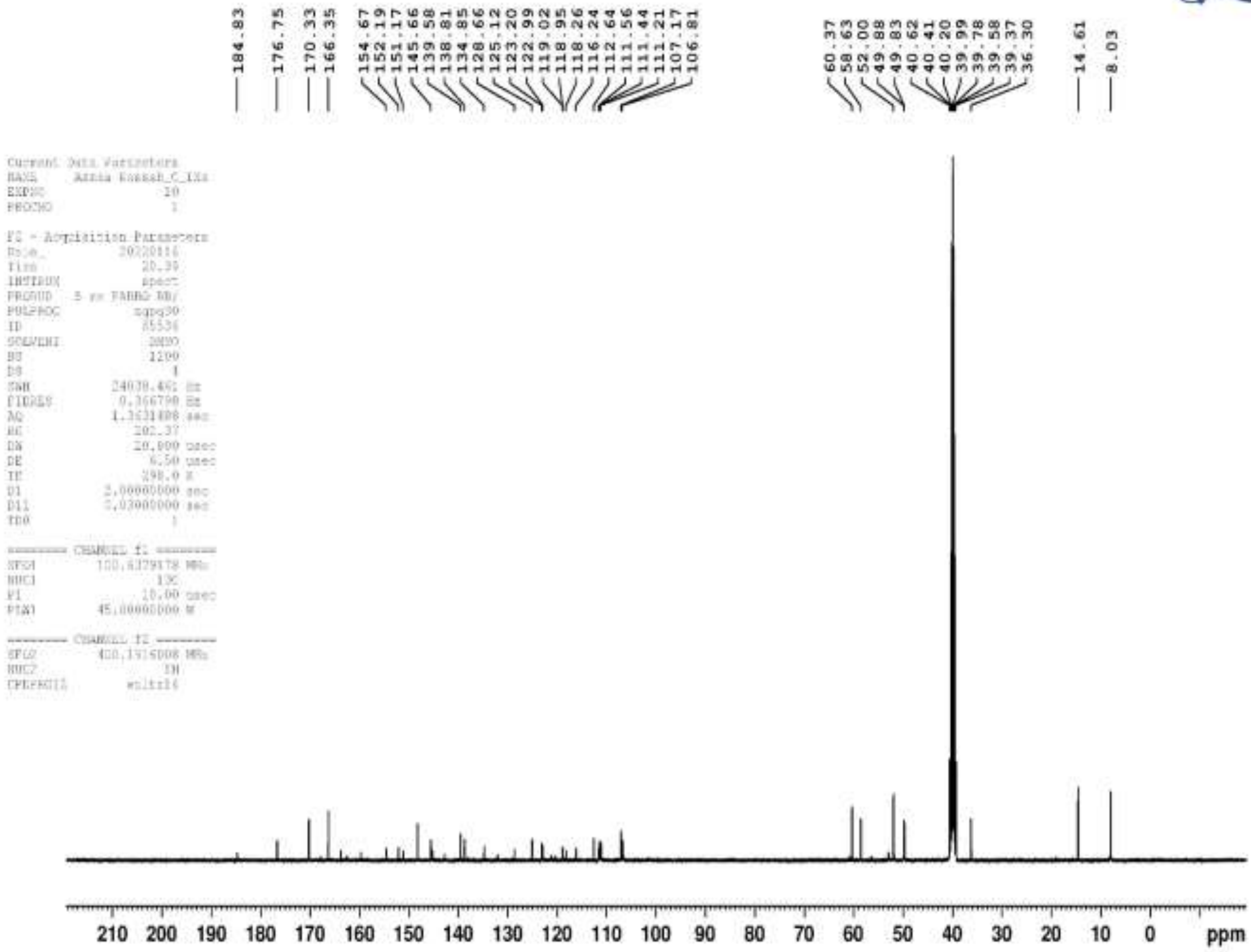

Compound 8b

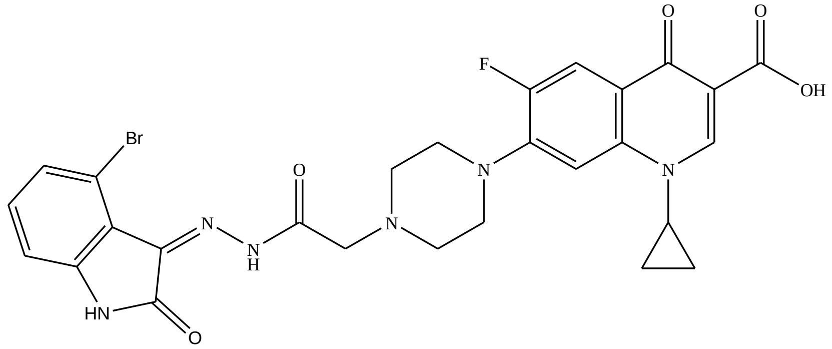

O
O
F
OH
Br
O
N
N
N
N
N
H
HN
O

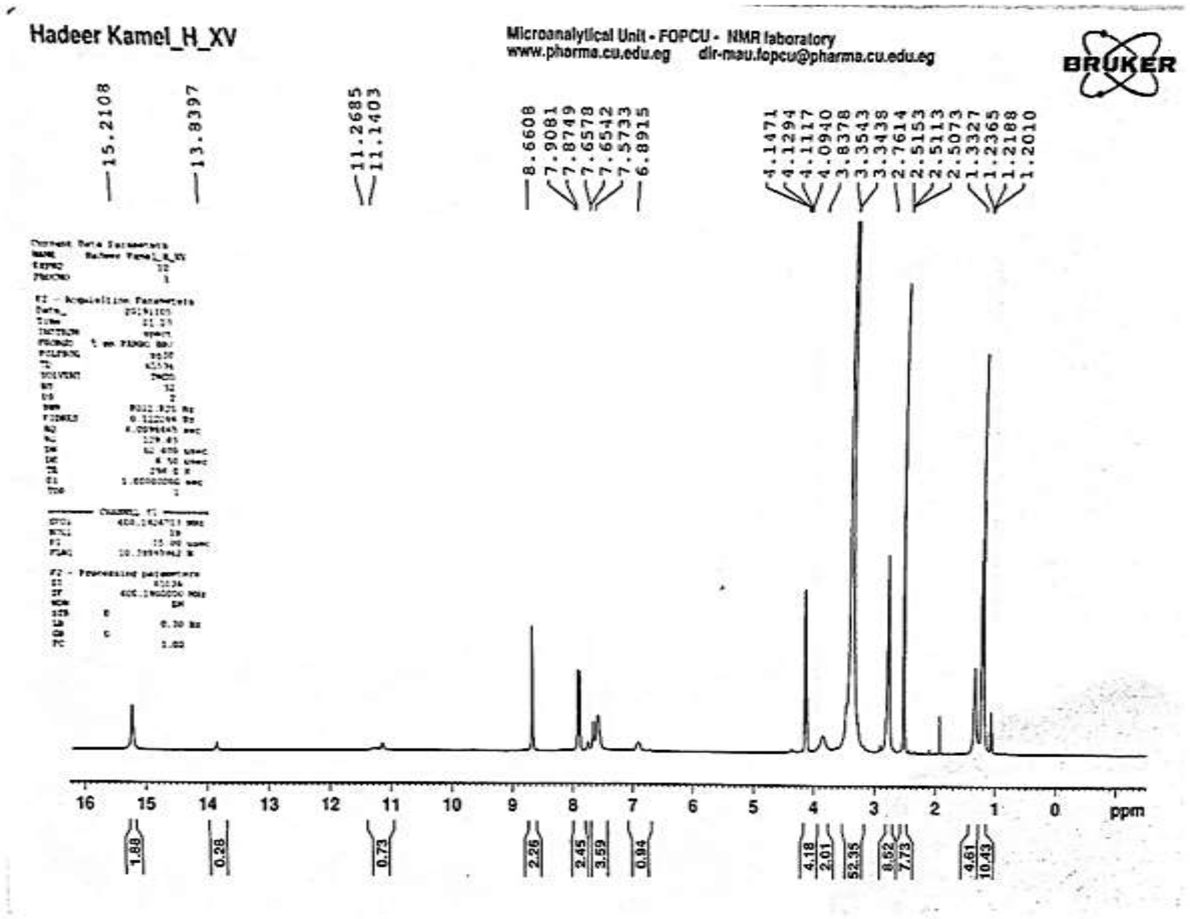

Hadeer Kamel_H_XV
Microanalytical Unit - FOPCU - NMR laboratory
www.pharma.cu.edu.eg dir-mau.fopcu@pharma.cu.edu.eg
BRUKER
15.2108
13.8397
11.2685
11.1403
8.6608
7.9081
7.8749
7.6578
7.6542
7.5733
6.8915
4.1471
4.1294
4.1117
4.0940
3.8378
3.3543
3.3438
2.7614
2.5153
2.5113
2.5073
1.3327
1.2365
1.2188
1.2010
1.88
0.28
0.73
2.26
2.45
3.59
0.94
4.18
2.01
52.35
8.52
7.73
4.61
10.43
ppm

Asmaa Kassab_C_IXb

Microanalytical Unit - FOPCU - NMR laboratory
www.pharma.cu.edu.eg dir-mau.fopcu@pharma.cu.edu.eg

BRUKER

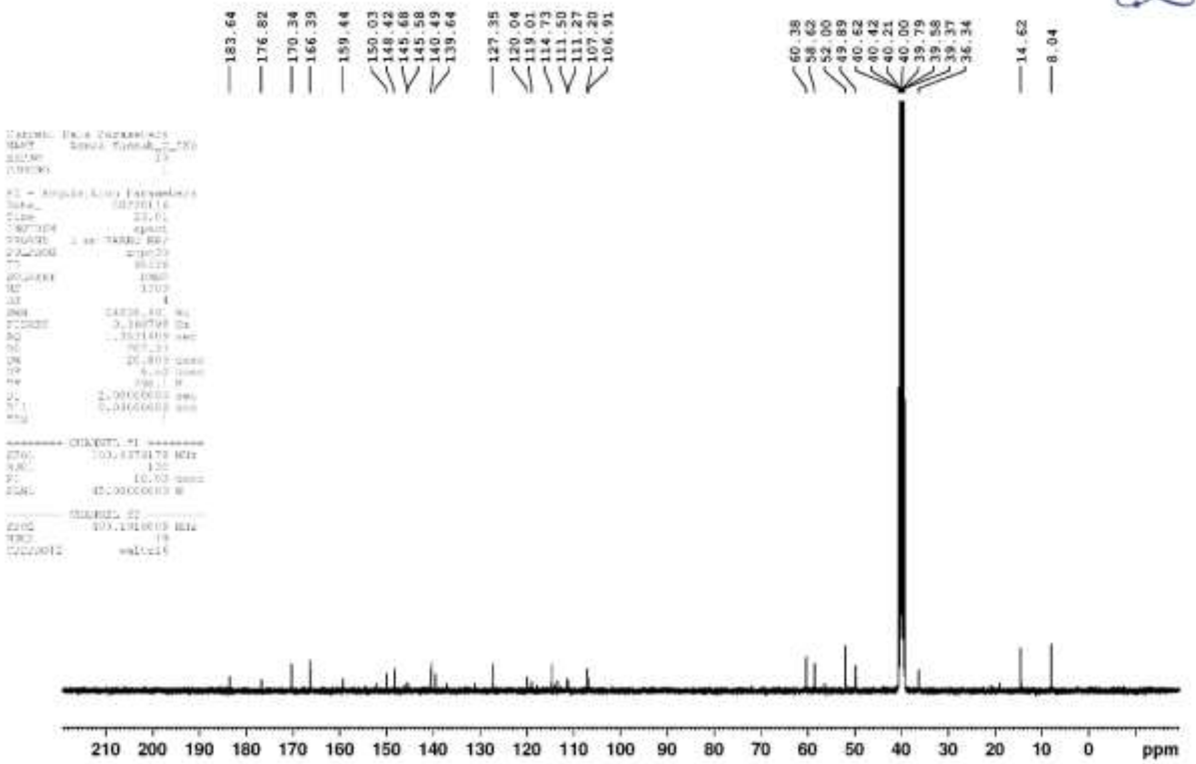

Information Classification: General

Compound 9a

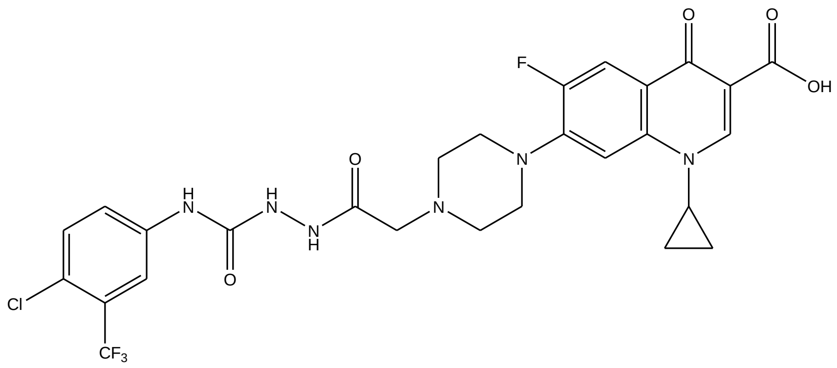

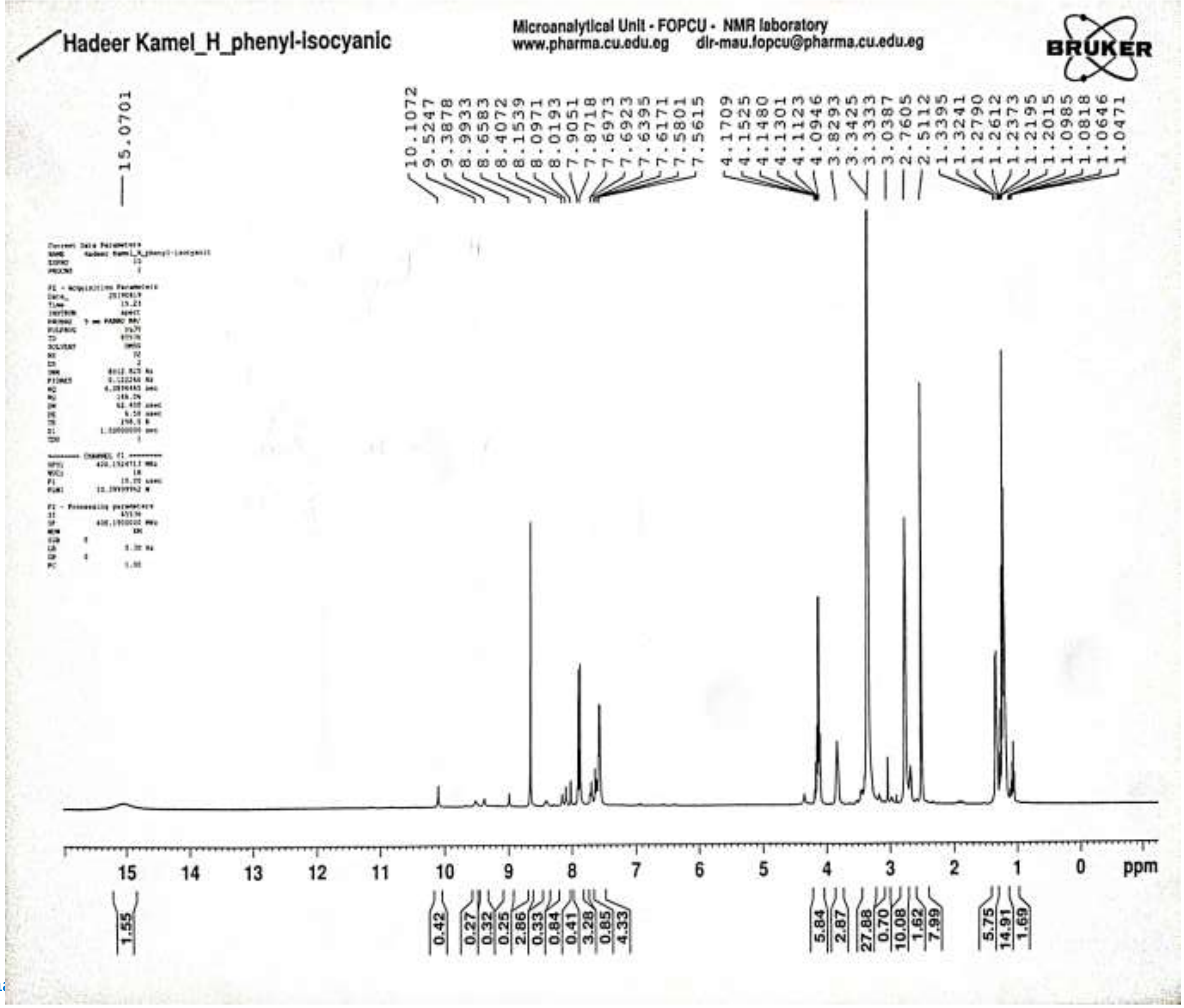
Hadeer Kamel_H_phenyl-isocyanic
Microanalytical Unit - FOPCU - NMR laboratory
www.pharma.cu.edu.eg dir-mau.fopcu@pharma.cu.edu.eg
ppm

Asmaa Kassab_C_Xa

Microanalytical Unit - FOPCU - NMR laboratory
www.pharma.cu.edu.eg dir-mau.fopcu@pharma.cu.edu.eg

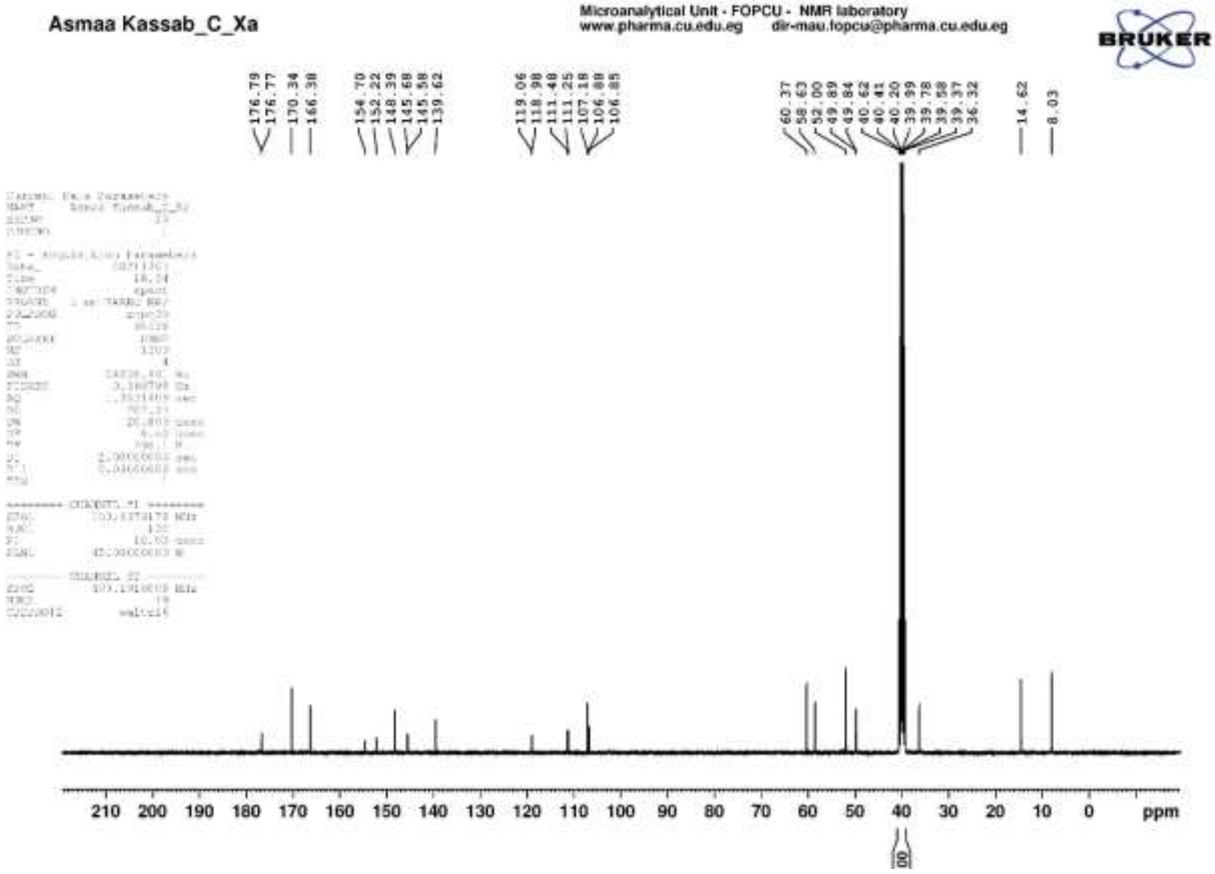

Compound 9b

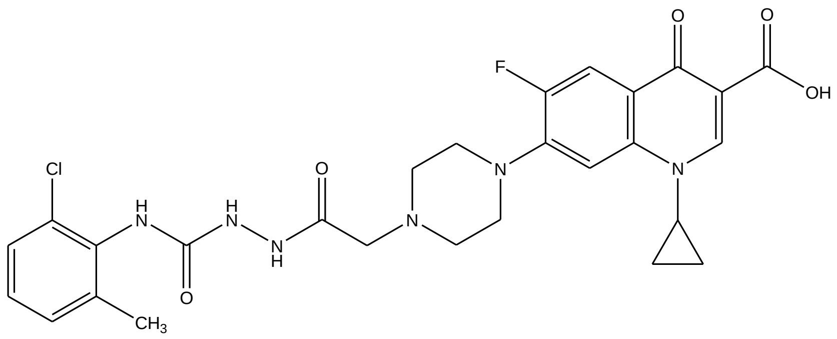

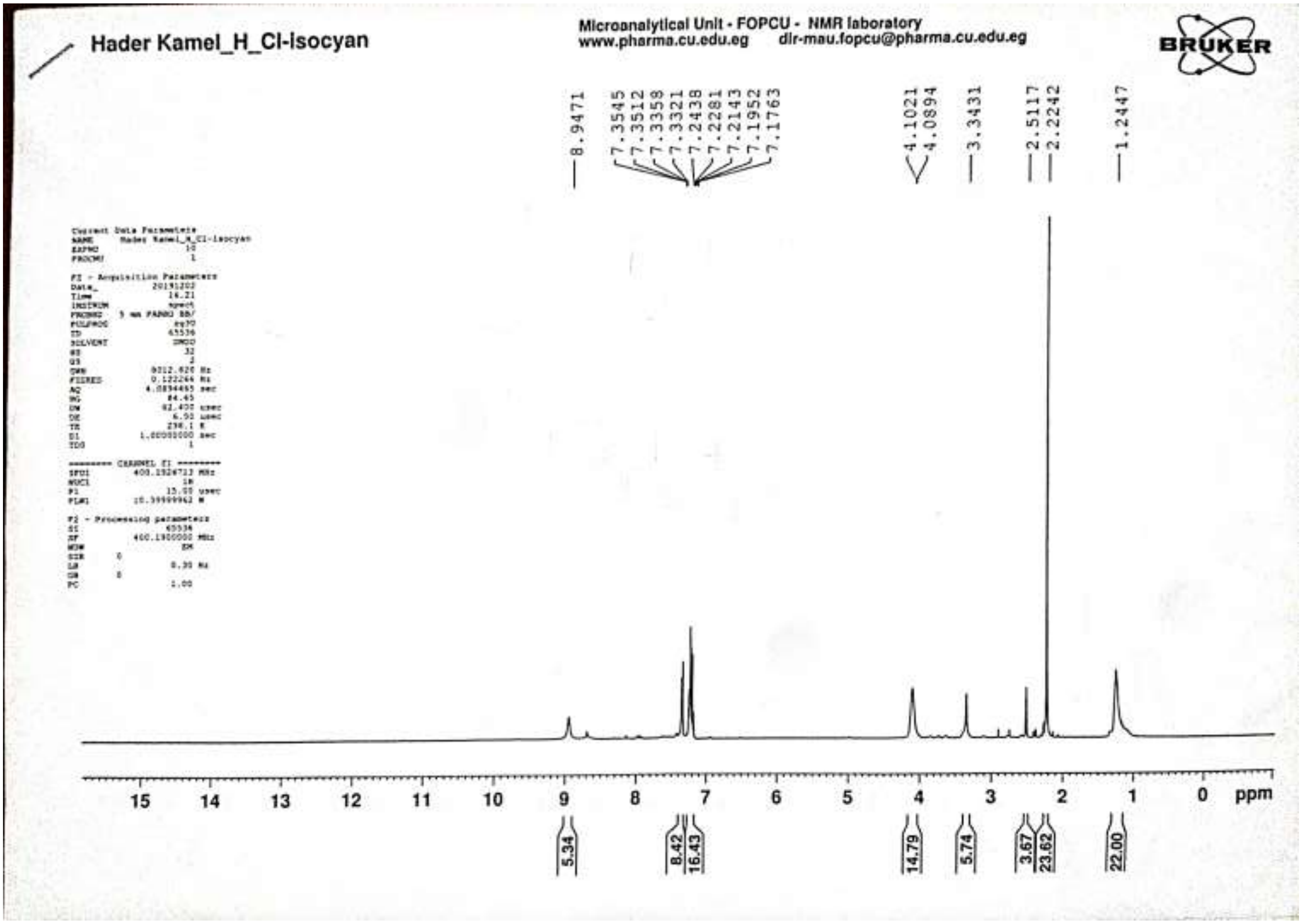

Information Classification: General

Asmaa Kassab_C_Xb

Microanalytical Unit - FOPCU - NMR laboratory
www.pharma.cu.edu.eg dir-mau.fopcu@pharma.cu.edu.eg

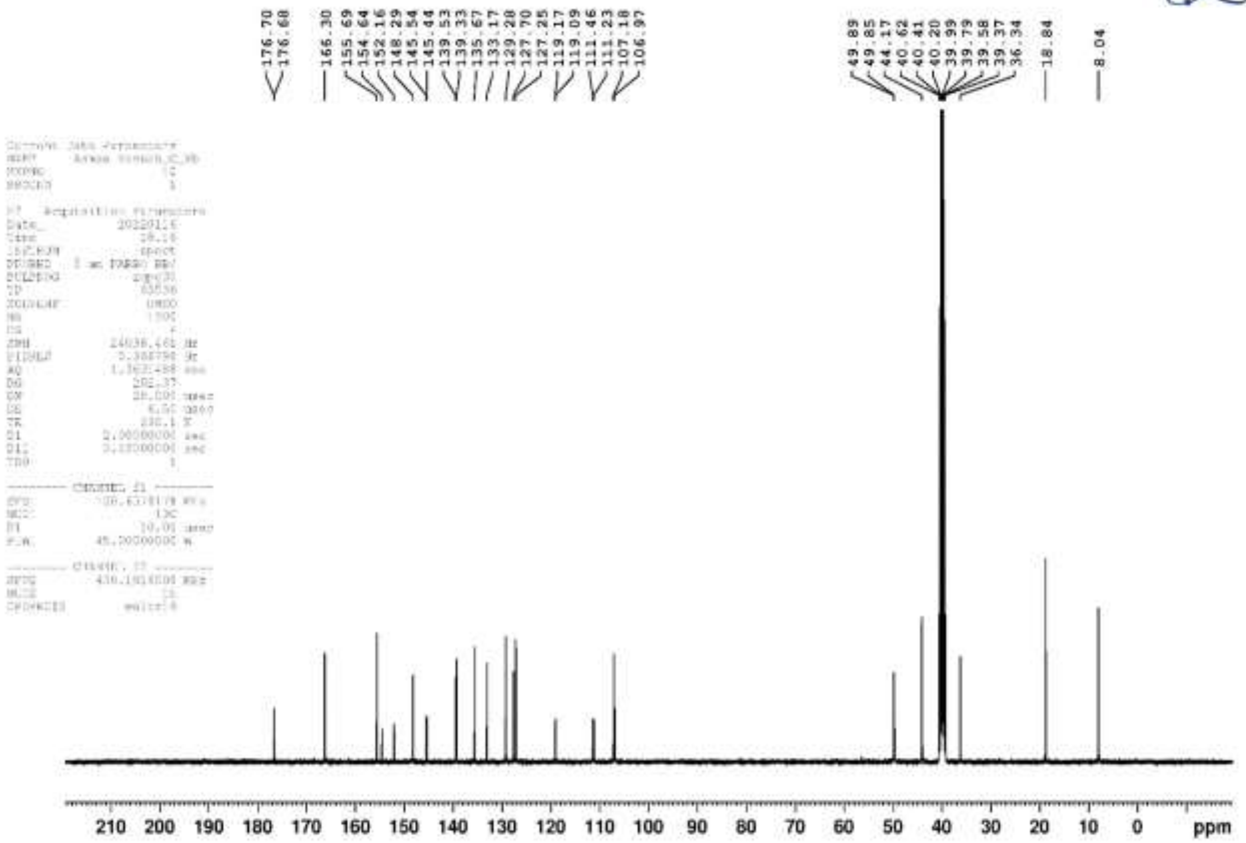

Information Classification: General

Compound 9c

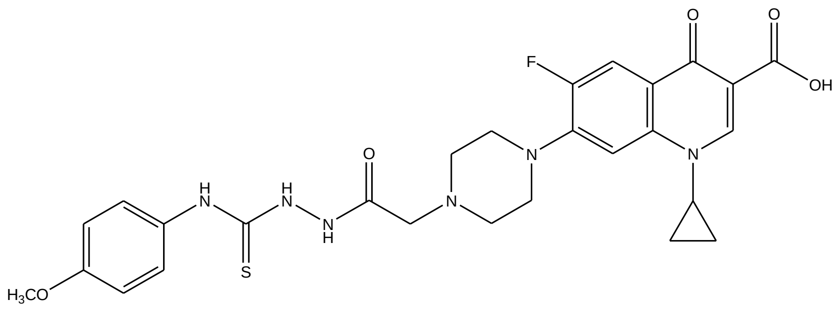
O
O
F
OH
N
N
O
H
N
H
N
N
N
H
H3CO
S

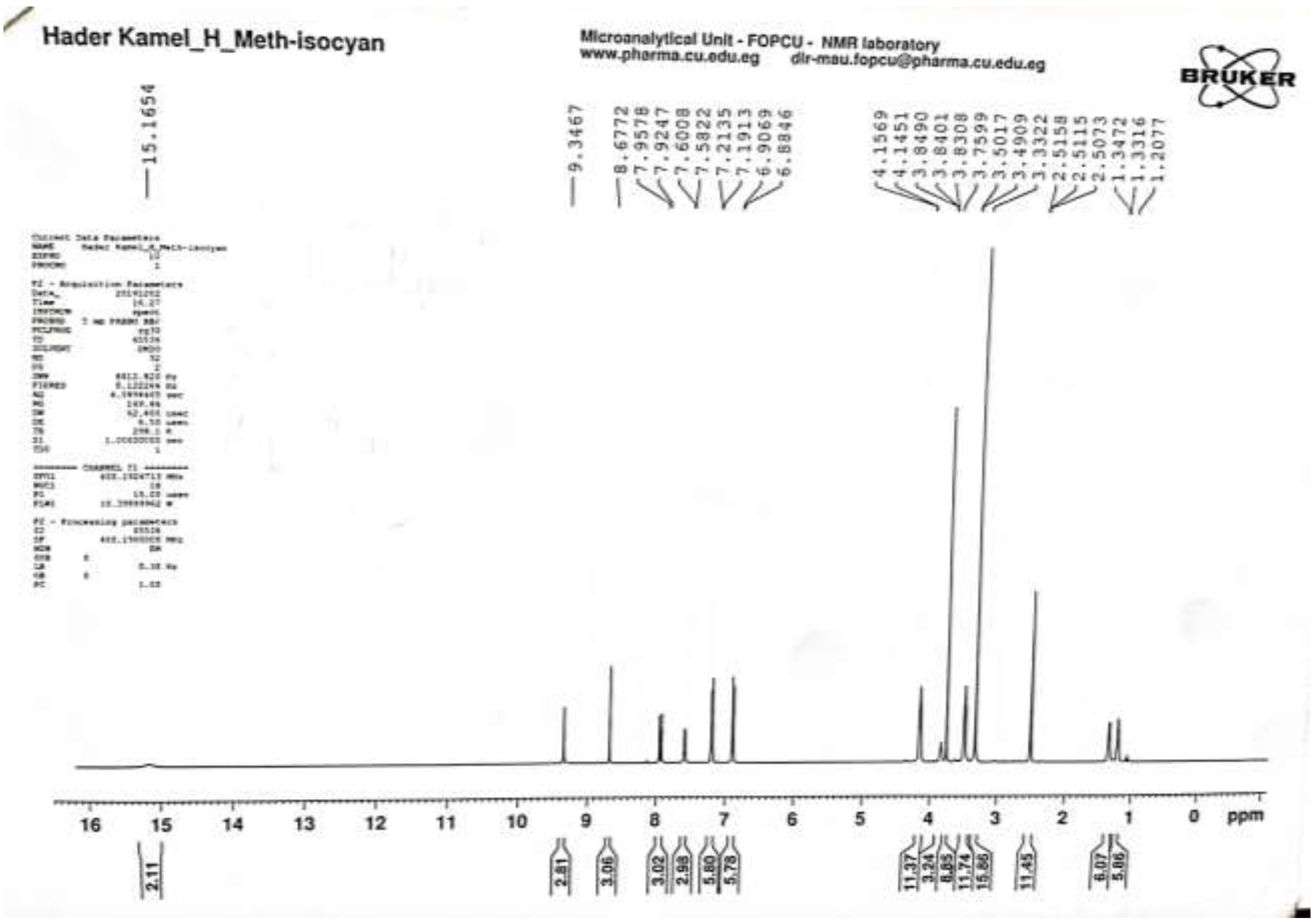
Hader Kamel_H_Meth-isocyan
Microanalytical Unit - FOPCU - NMR laboratory
www.pharma.cu.edu.eg dir-mau.fopcu@pharma.cu.edu.eg
BRUKER
15.1654
9.3467
8.6772
7.9578
7.9247
7.6008
7.5822
7.2135
7.1913
6.9069
6.8846
4.1569
4.1451
3.8490
3.8401
3.8308
3.7599
3.5017
3.4909
3.3322
2.5158
2.5115
2.5073
1.3472
1.3316
1.2077
16 15 14 13 12 11 10 9 8 7 6 5 4 3 2 1 0 ppm
2.11
2.81
3.06
3.02
2.98
5.80
5.78
11.37
3.24
8.85
11.74
15.86
11.45
6.07
5.86

Hadeer Kamel_C_Methoxy-iso-Cyn

Microanalytical Unit - FOPCU - NMR laboratory
www.pharma.cu.edu.eg dir-mau.fopcu@pharma.cu.edu.eg

Current Data Parameters	
NAME	Hadeer Kamel_C_Methoxy-
EXPNO	10
PROCNO	1
F2 - Acquisition Parameters	
Date_	20191224
Time	11.22
INSTRUM	spect
PROBHD	5 mm PABBO BB/
PULPROG	zgpg30
TD	65536
SOLVENT	DMSO
NS	300
DS	4
SWH	24038.461 Hz
FIDRES	0.366798 Hz
AQ	1.3631488 sec
RG	202.37
DW	20.800 usec
DE	6.50 usec
TE	298.1 K
D1	2.00000000 sec
D11	0.03000000 sec
TD0	1
======== CHANNEL f1 ========	
SFO1	100.6379178 MHz
NUC1	13C
P1	10.00 usec
PLW1	45.00000000 W
======== CHANNEL f2 ========	
SFO2	400.1916008 MHz
NUC2	1H
CPDPRG[2	waltz16

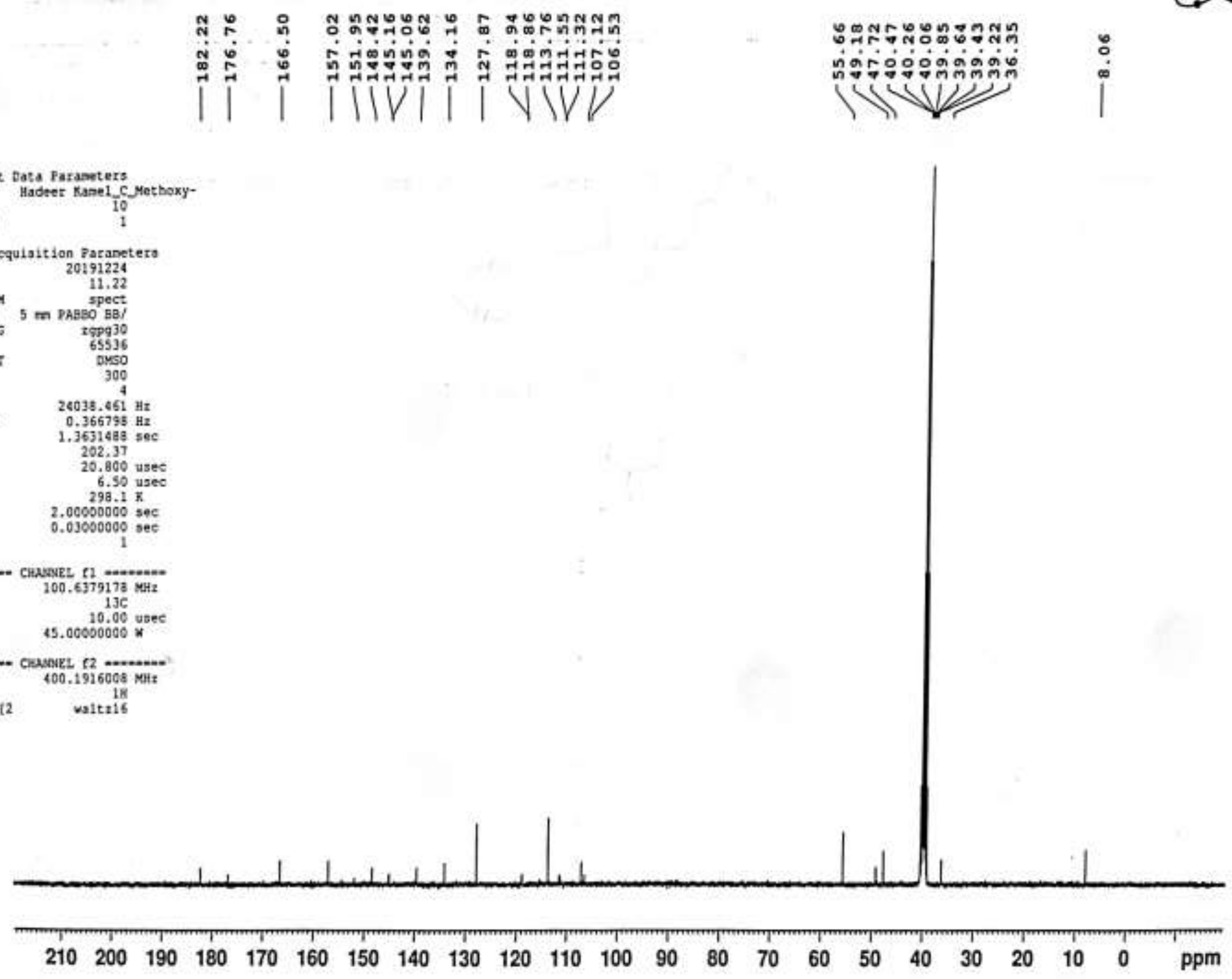

Information Classification: General

Compound 10a

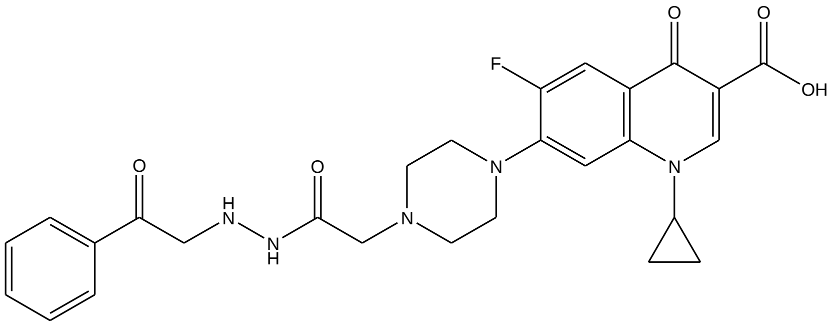

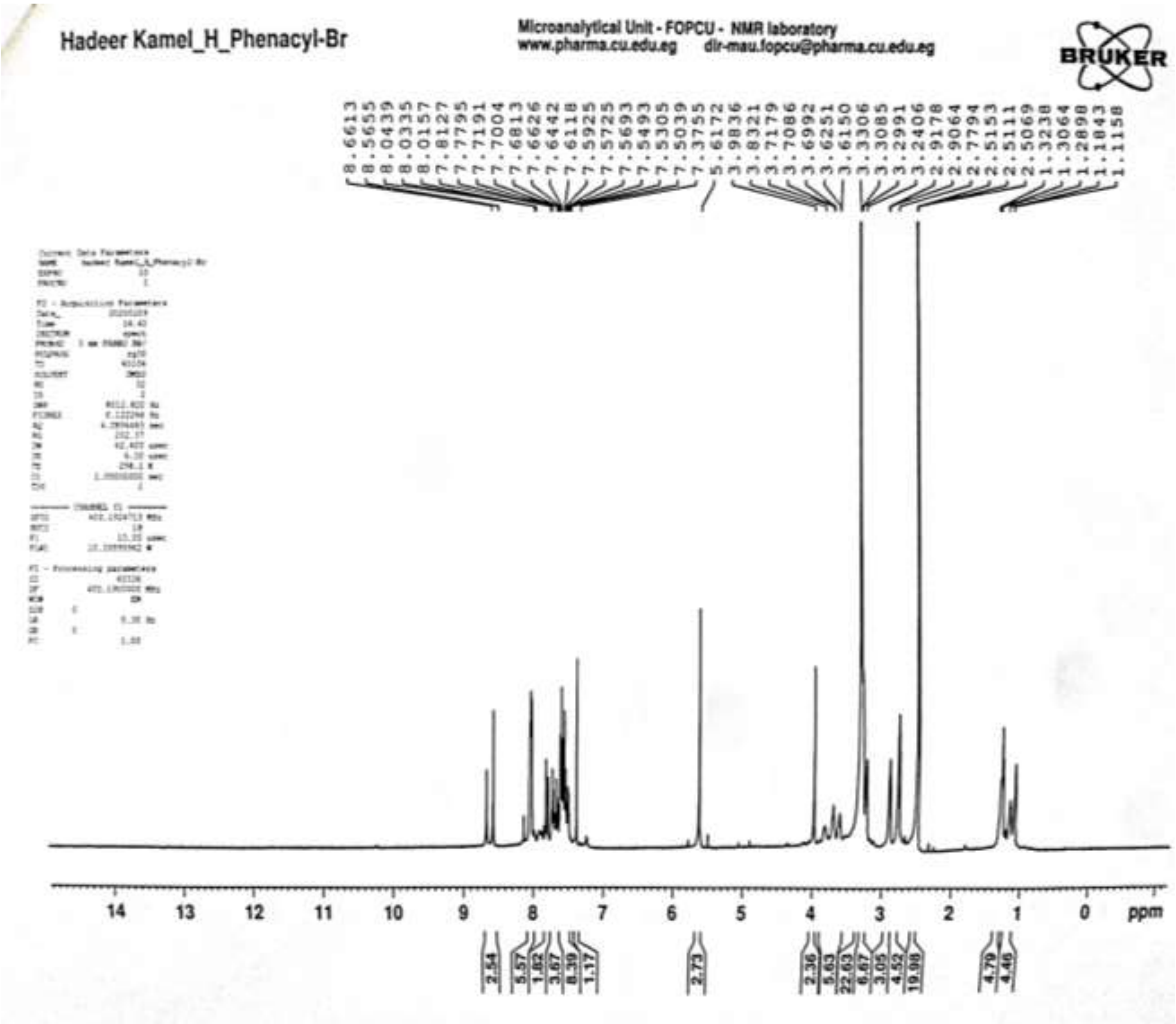

Compound 10b

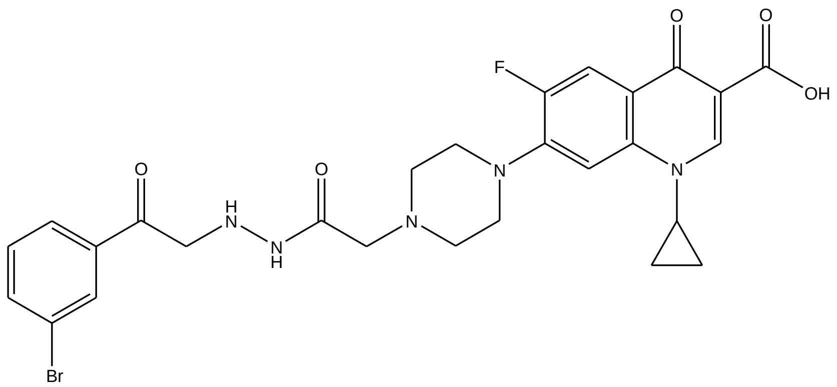

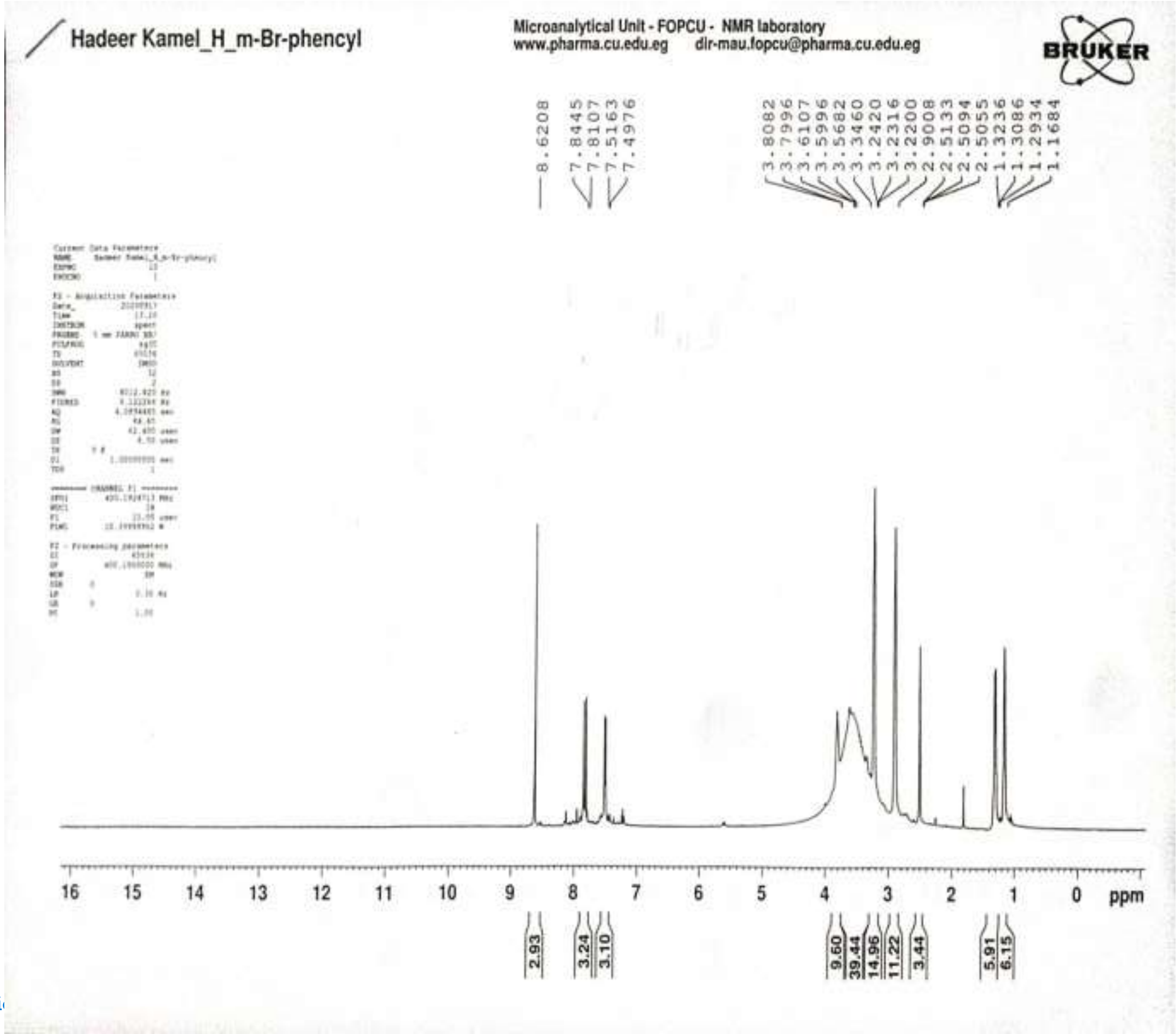

Informati

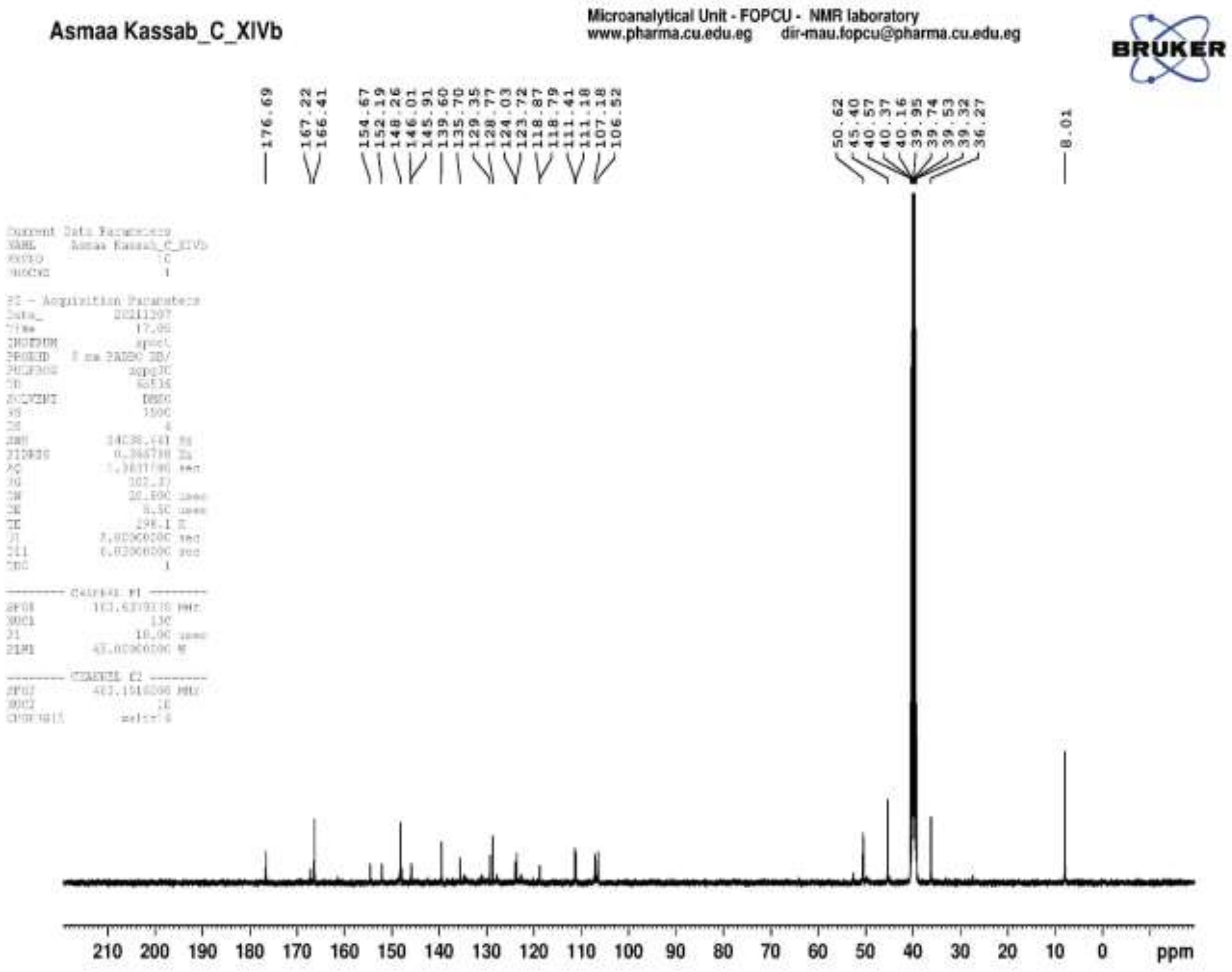
Asmaa Kassab_C_XIVb
Microanalytical Unit - FOPCU - NMR laboratory
www.pharma.cu.edu.eg dir-mau.fopcu@pharma.cu.edu.eg
BRUKER
176.69
167.22
166.41
154.67
152.19
148.26
146.01
145.91
139.60
135.70
129.35
128.77
124.03
123.72
118.87
118.79
111.41
111.18
107.18
106.52
50.62
45.40
40.57
40.37
40.16
39.95
39.74
39.53
39.32
36.27
8.01
210 200 190 180 170 160 150 140 130 120 110 100 90 80 70 60 50 40 30 20 10 0 ppm

Compound 10c

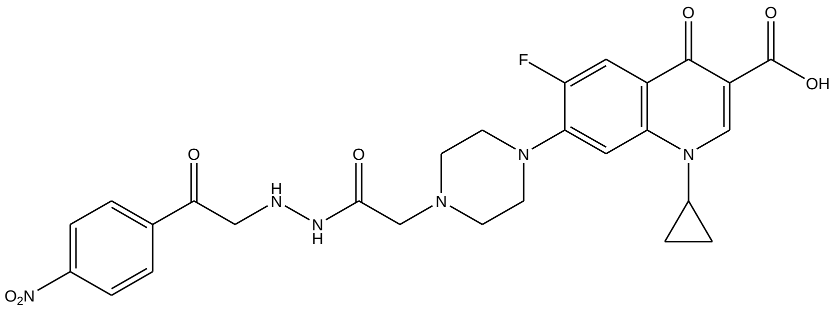

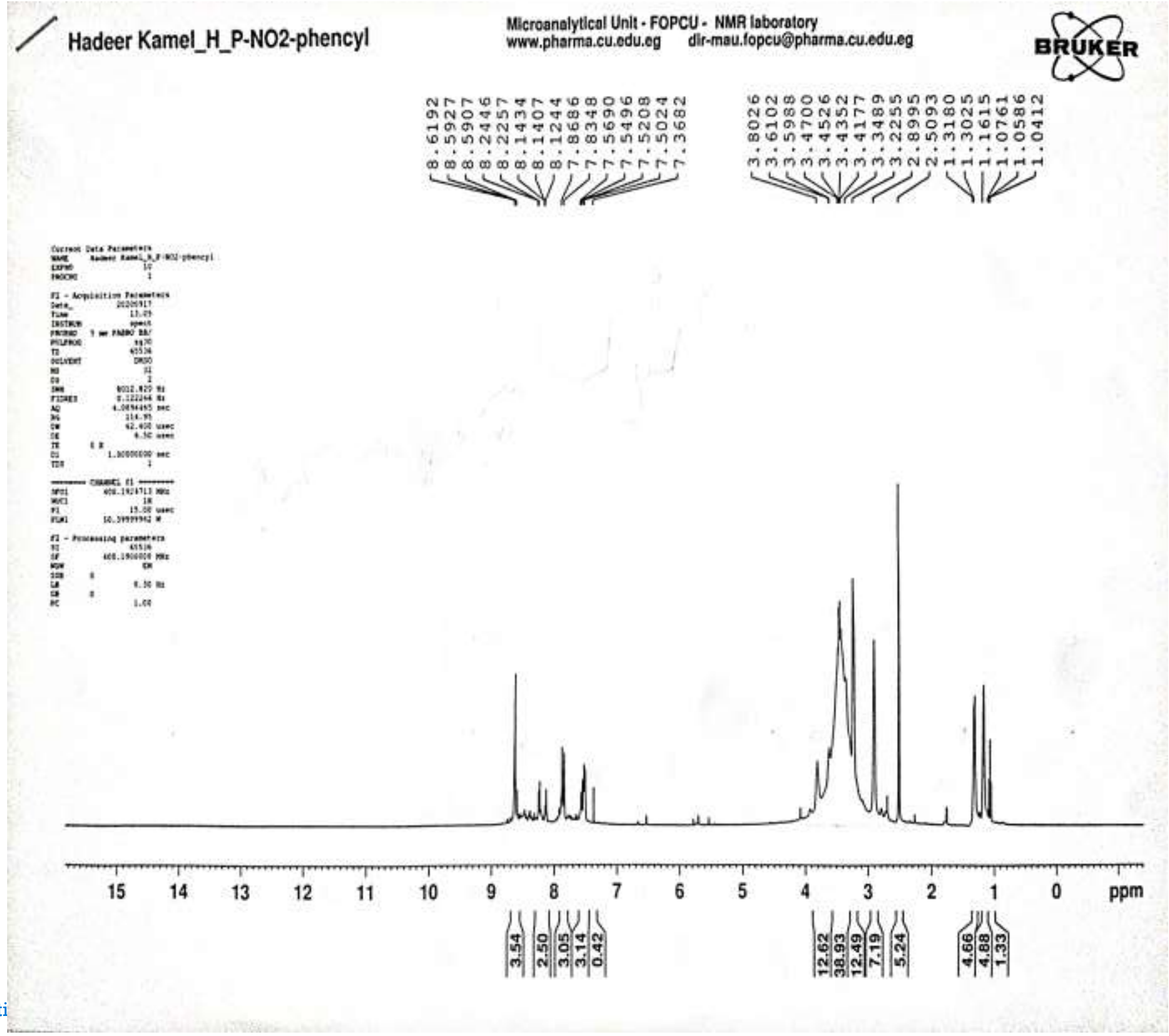

Informati

Asmaa Kassab_C_XIVC

Microanalytical Unit - FOPCU - NMR laboratory
www.pharma.cu.edu.eg dir-mau.fopcu@pharma.cu.edu.eg

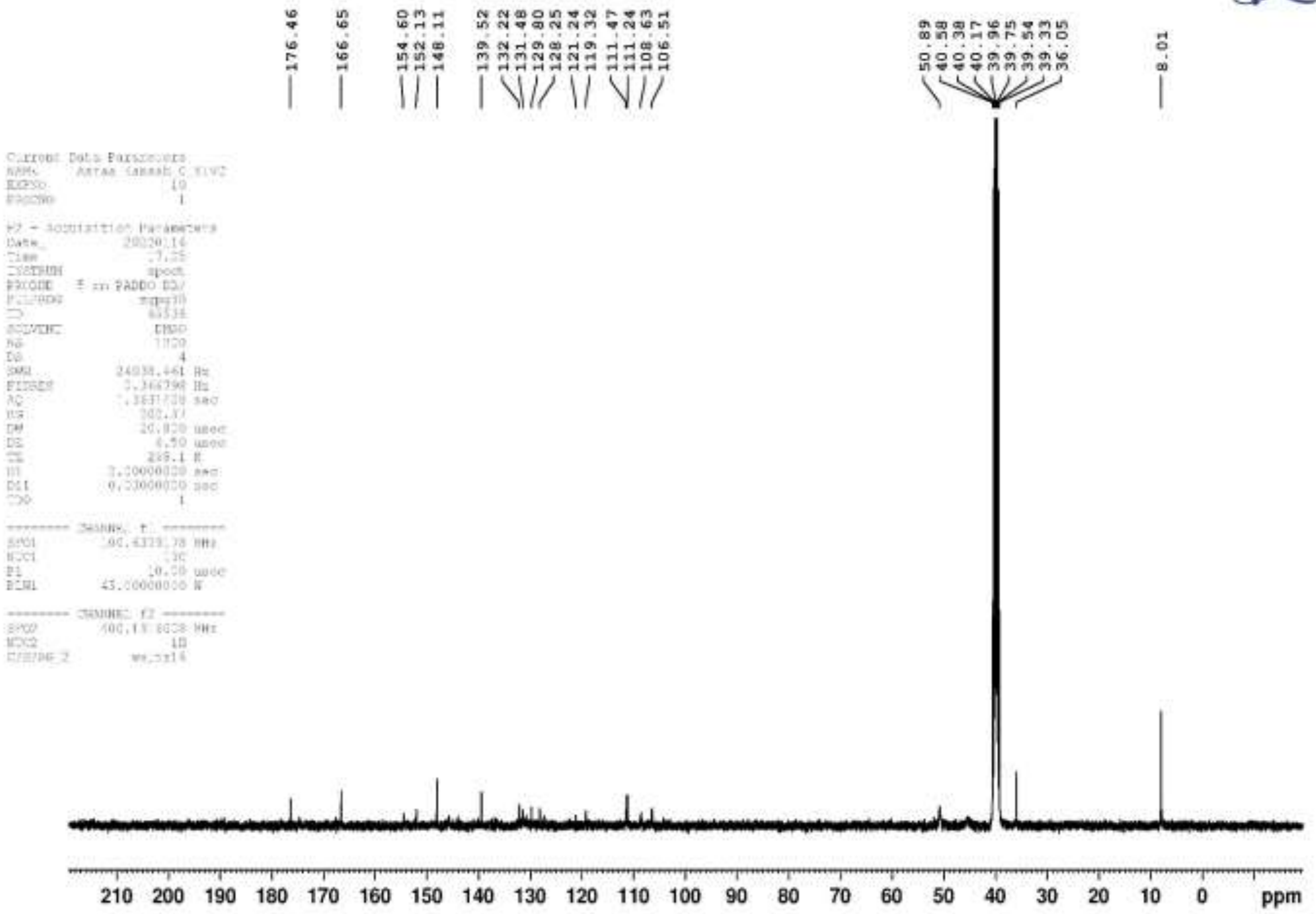

Compound 11

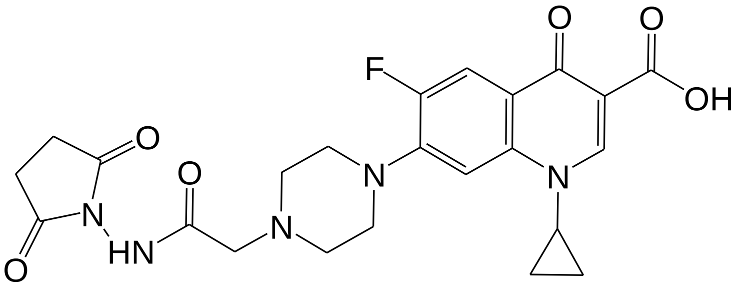

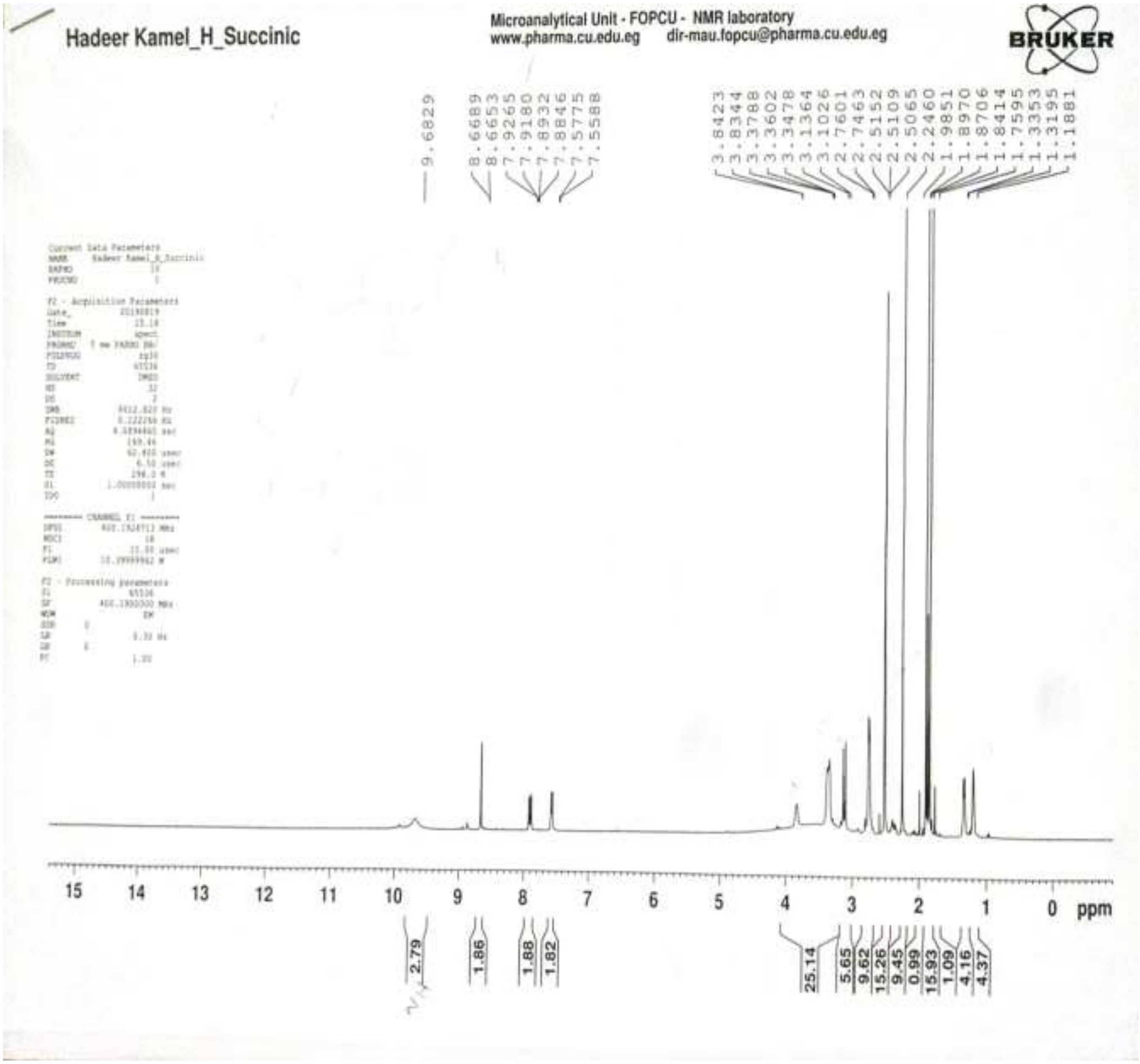

Information Classification: General

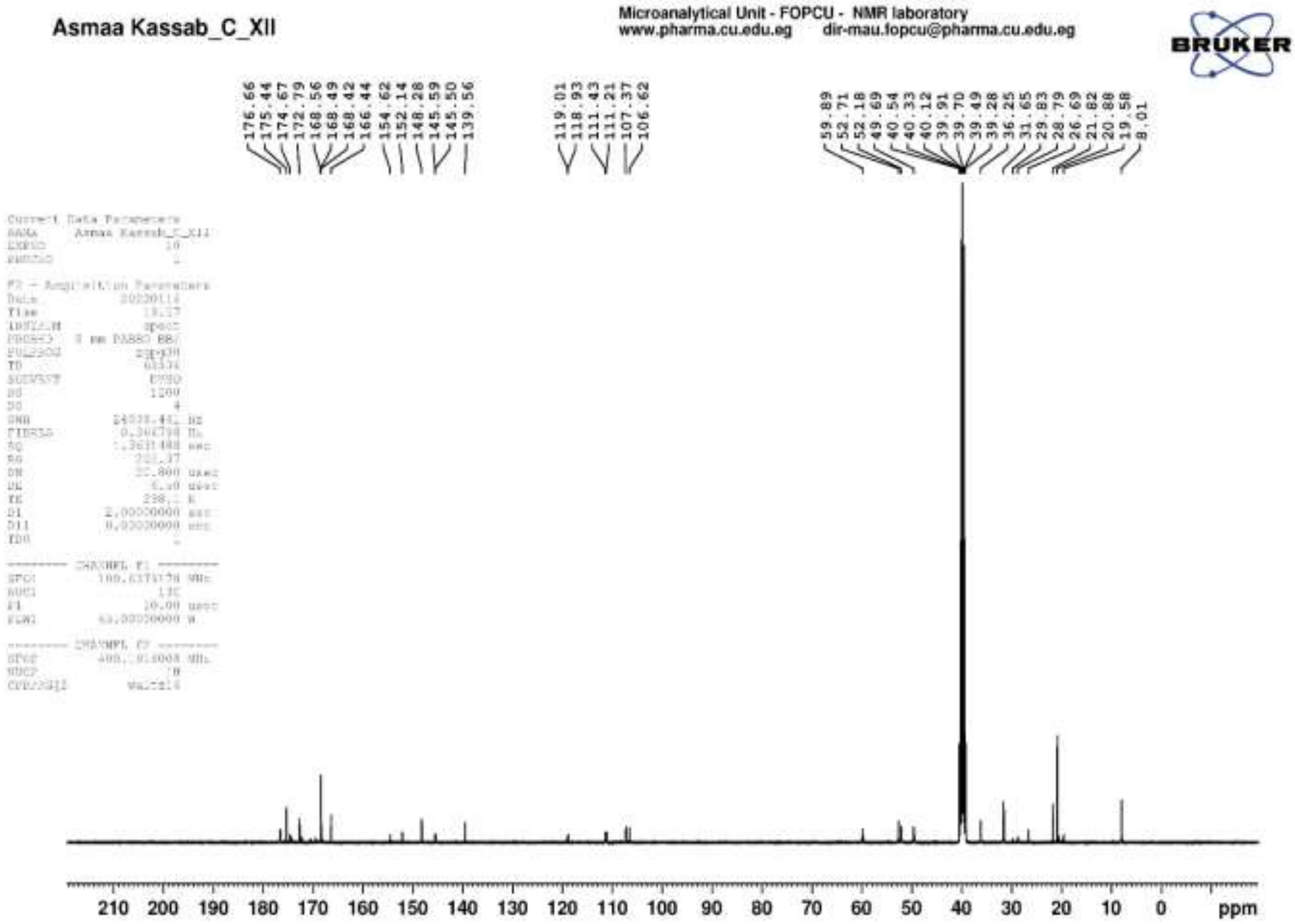
Asmaa Kassab_C_XII
Microanalytical Unit - FOPCU - NMR laboratory
www.pharma.cu.edu.eg dir-mau.fopcu@pharma.cu.edu.eg
BRUKER
176.66
175.44
174.67
172.79
168.56
168.49
168.42
166.44
154.62
152.14
148.28
145.59
145.50
139.56
119.01
118.93
111.43
111.21
107.37
106.62
59.89
52.71
52.18
49.69
40.54
40.33
40.12
39.91
39.70
39.49
39.28
36.25
31.65
29.83
28.79
26.69
21.82
20.88
19.58
8.01
210 200 190 180 170 160 150 140 130 120 110 100 90 80 70 60 50 40 30 20 10 0 ppm

Compound 12

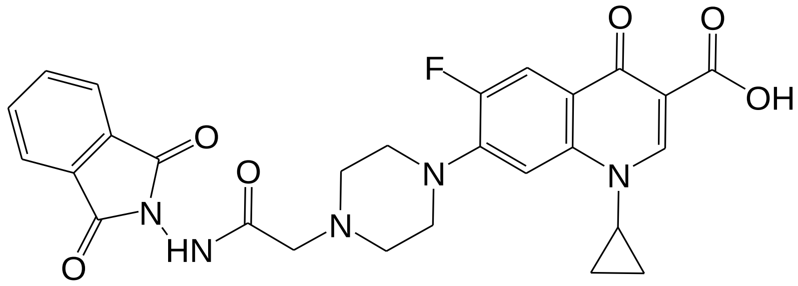

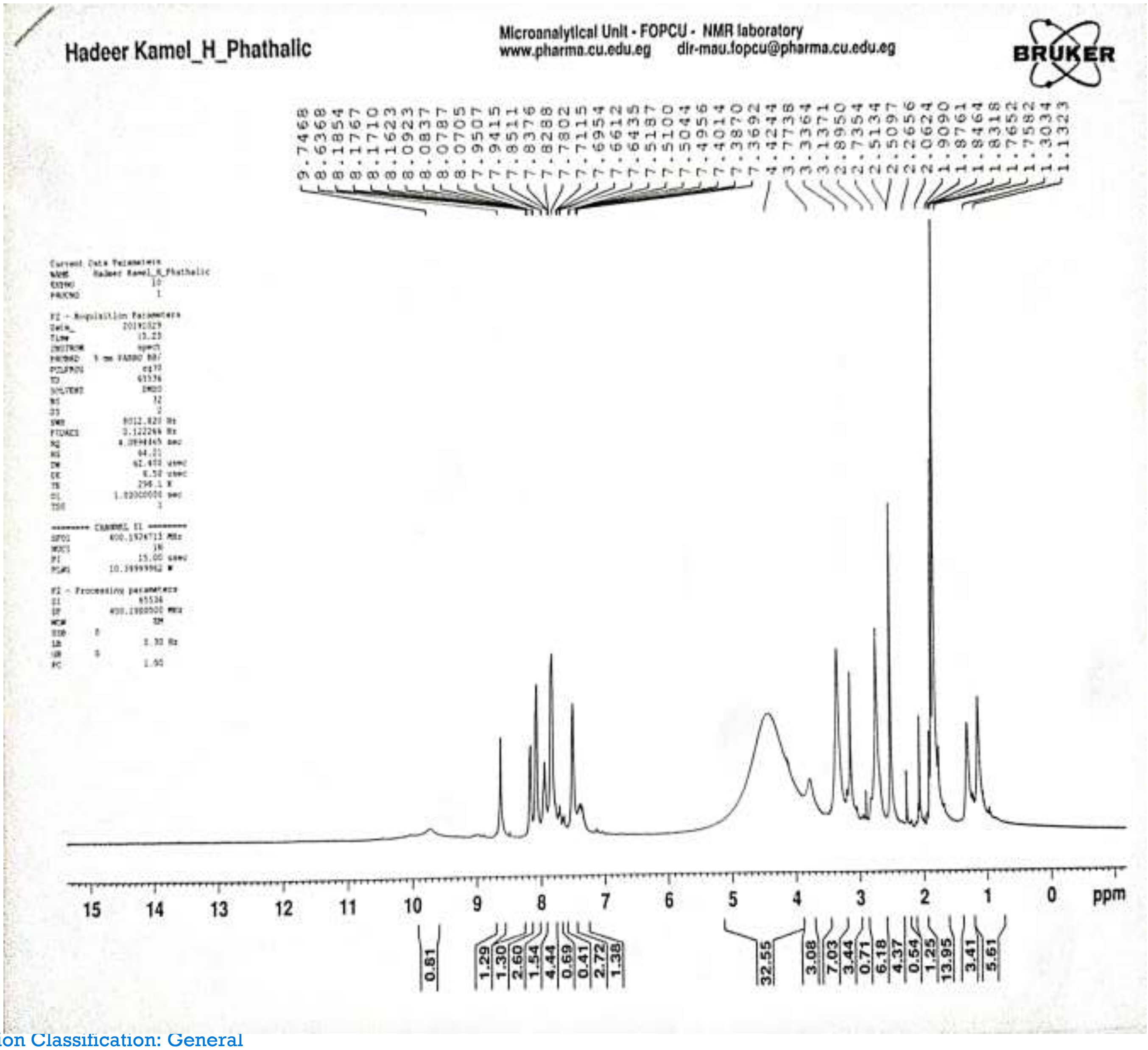

Information Classification: General

Compound 13

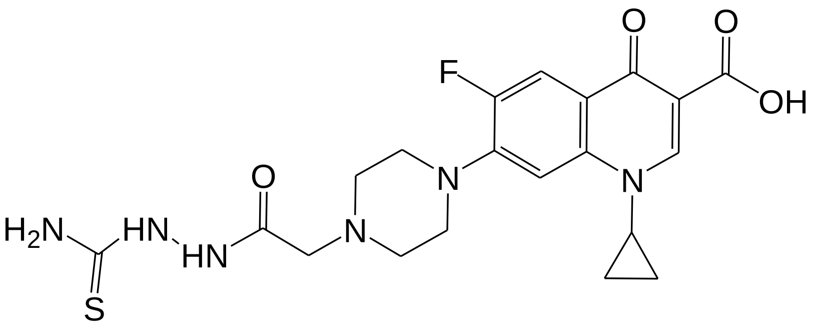

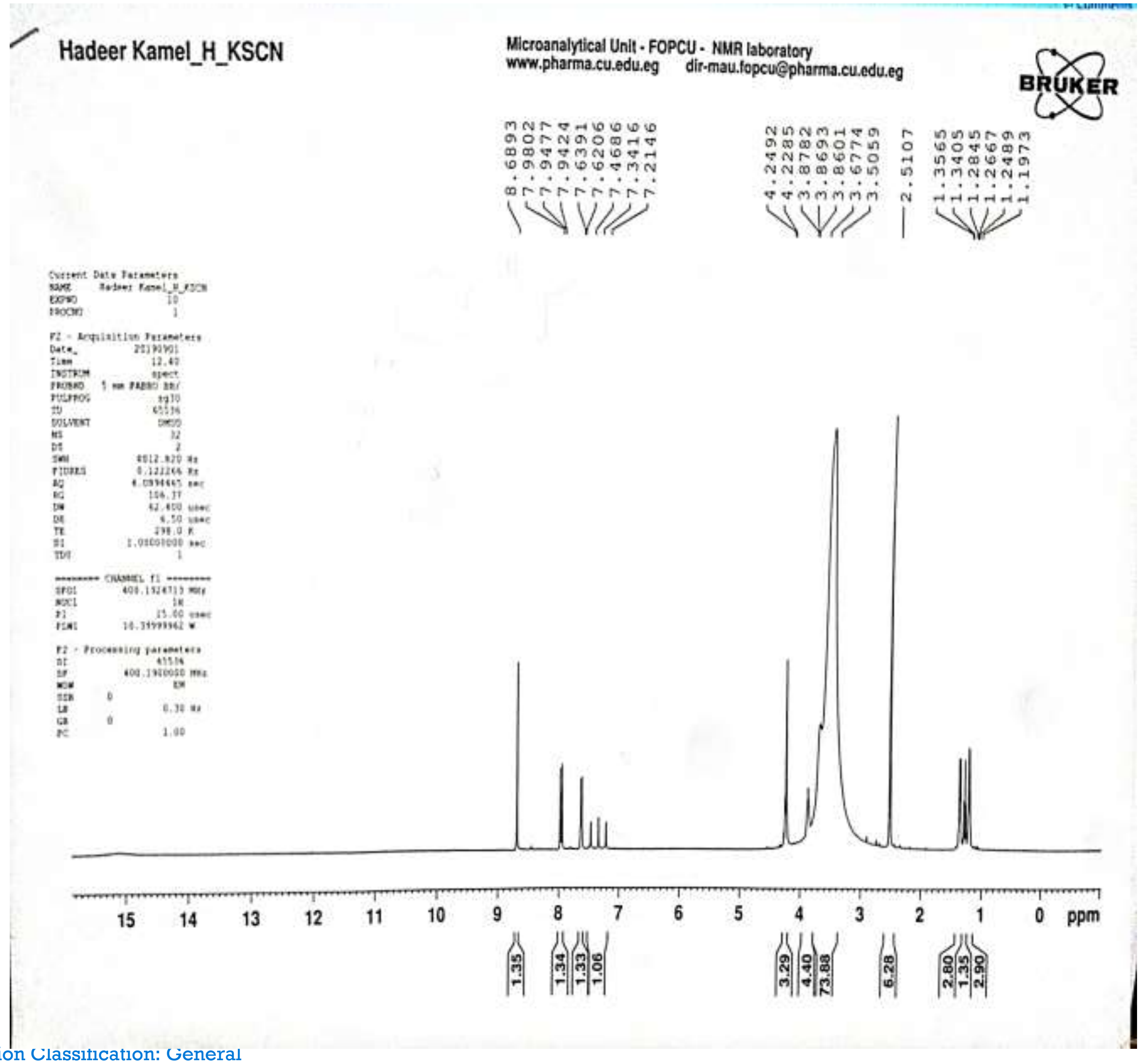

Information Classification: General

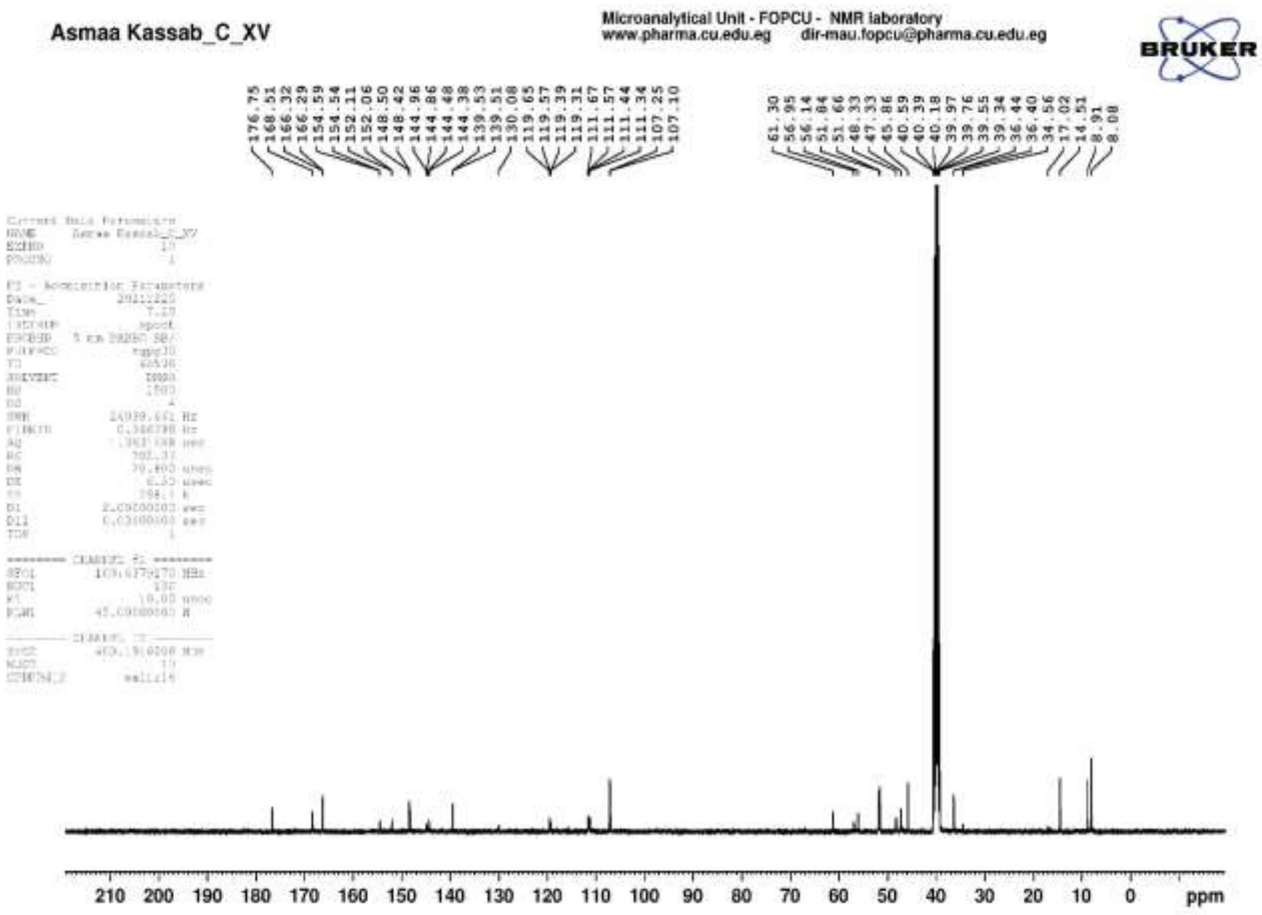

Information Classification: General

Compound 14

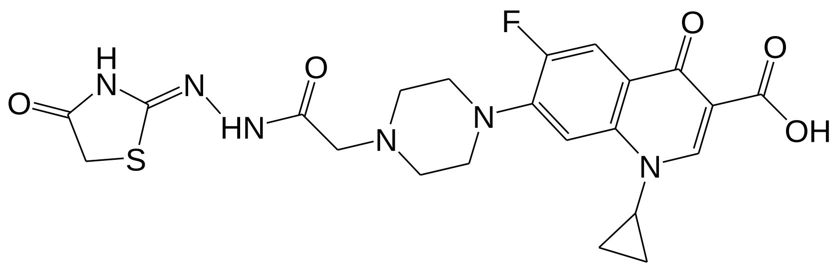

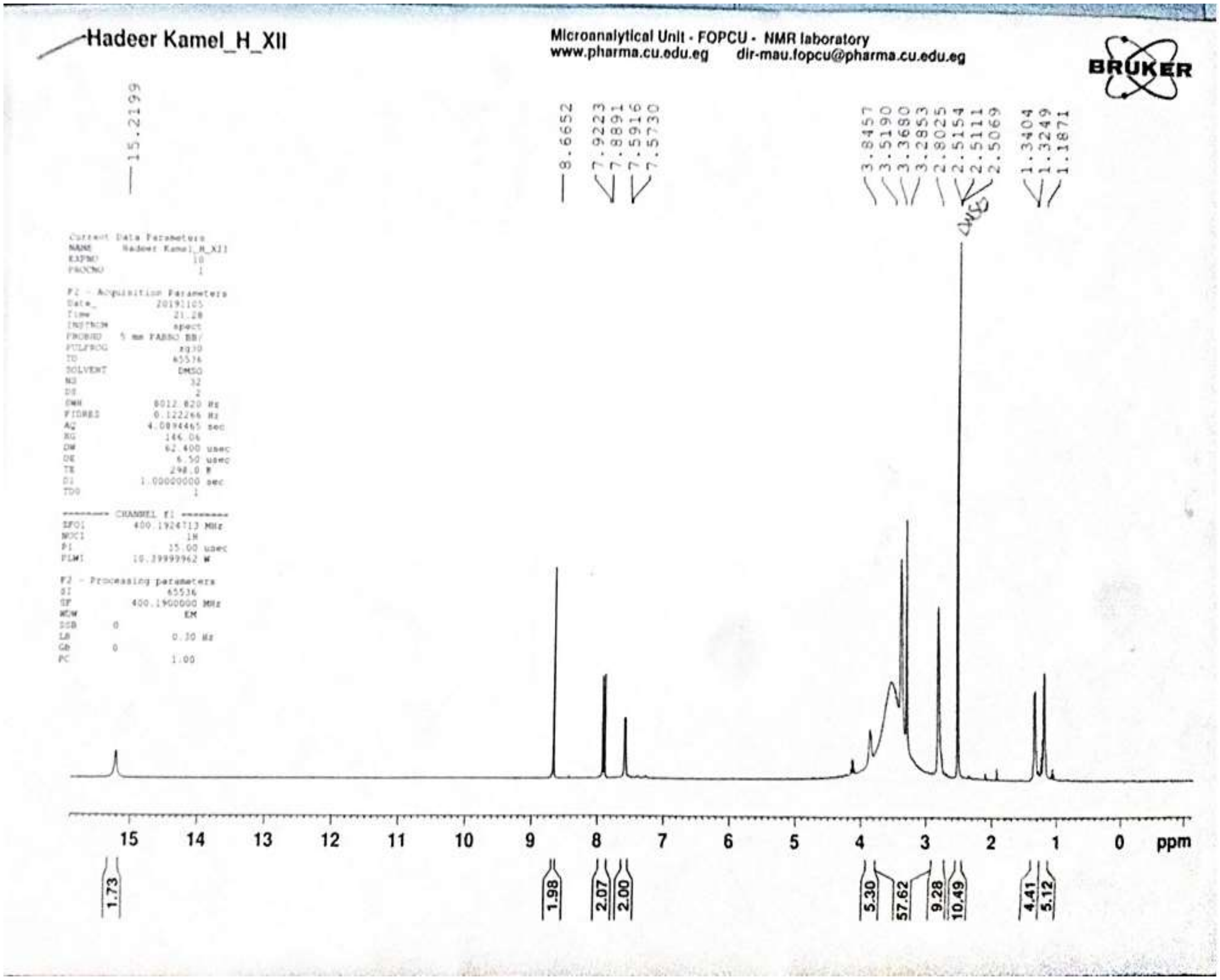

Information Classification: General

Compound 15

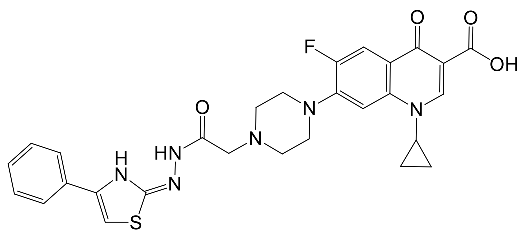

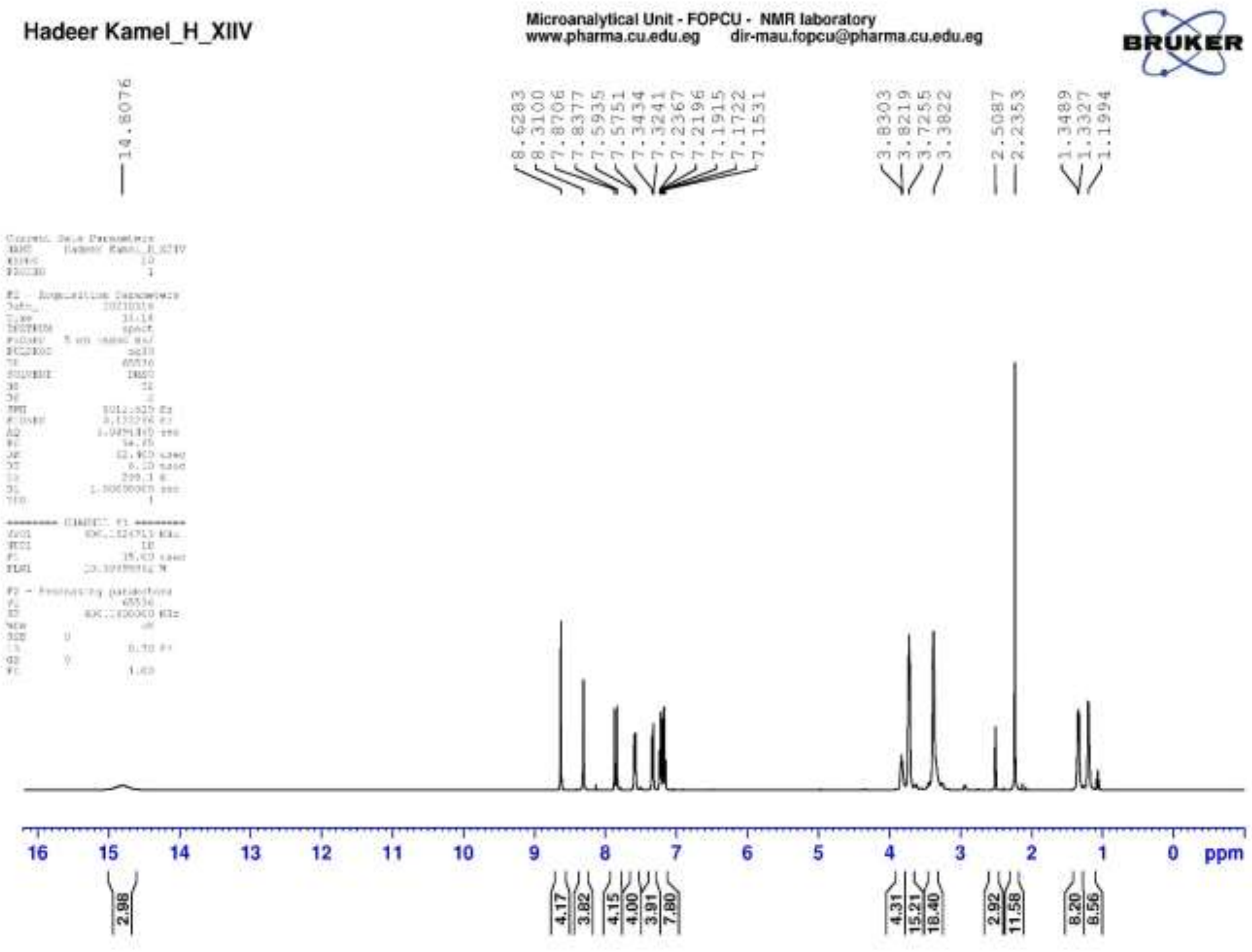

Information Classification: General

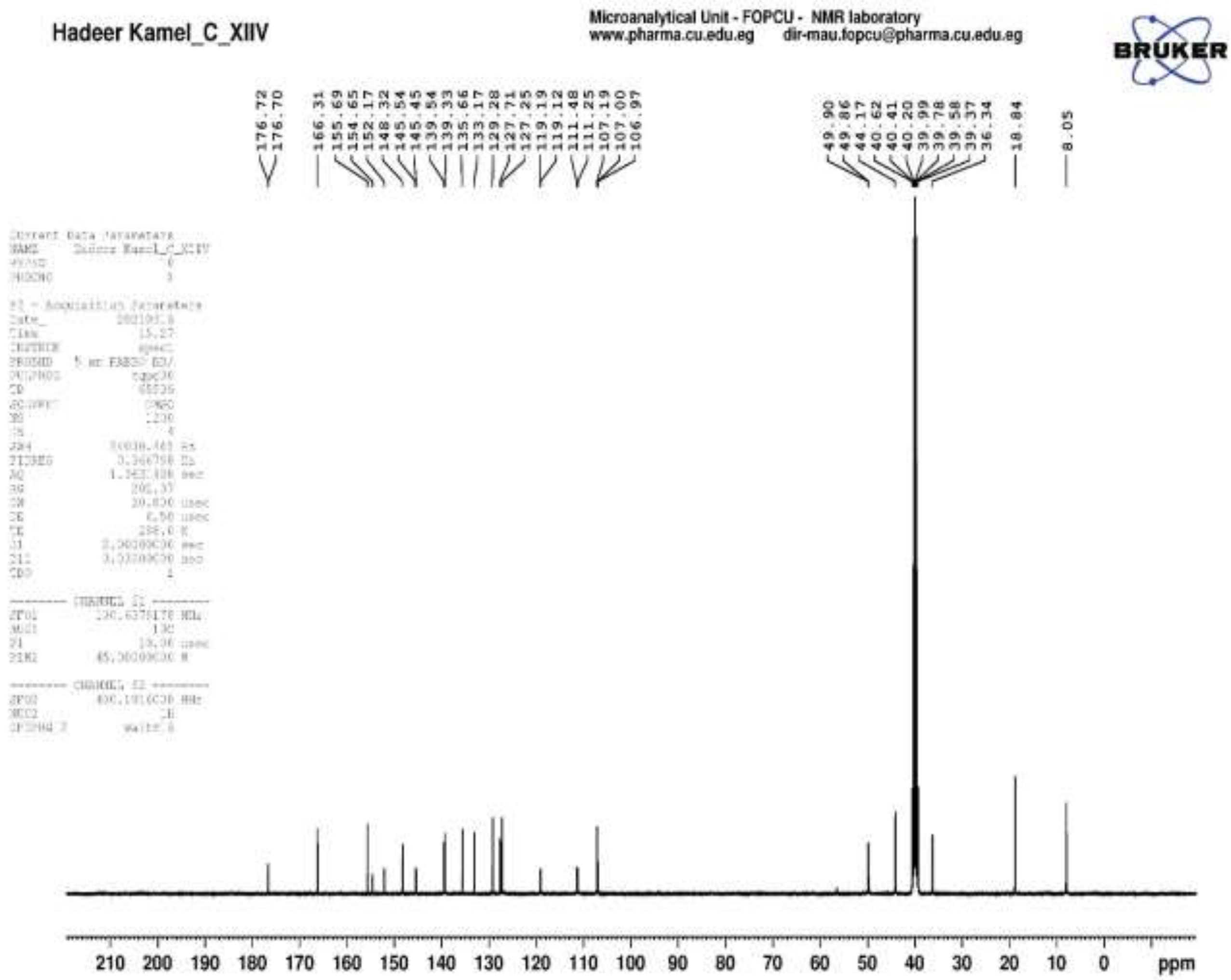

Information Classification: General
